# Supplementary material for: Myosin II Controls Junction Fluctuations to Guide Epithelial Tissue Ordering
Source: Dev Cell. 2017 Nov 20;43(4):480–492.e6. doi: 10.1016/j.devcel.2017.09.018 (PMC5703647; doi:10.1016/j.devcel.2017.09.018)
Supplement: Document S1. Figures S1–S7 and Table S1 [file mmc1.pdf]

Developmental Cell, Volume *43*

Supplemental Information

Myosin II Controls Junction Fluctuations

to Guide Epithelial Tissue Ordering

Scott Curran, Charlotte Strandkvist, Jasper Bathmann, Marc de Gennes, Alexandre Kabla, Guillaume Salbreux, and Buzz Baum

Supplemental Inventory

Supplemental Figures

Figure S1: Related to Figure 1.

Figure S2: Related to Figure 2.

Figure S3: Related to Figure 4.

Figure S4: Related to Figure 5.

Figure S5: Related to Figure 6.

Figure S6: Related to Figure 7.

Figure S7: Related to Figure 7.

Supplemental Tables

Table S1: Related to Figure 2A-D and Figure S2.

Supplemental Movies

Movie S1: Related to Figure 1.

Movie S2: Related to Figure 1.

Movie S3: Related to Figure 5.

Movie S4: Related to Figure 6.

Supplemental Theory

Methods S1: Related to STAR Methods

Supplementary Figure 1

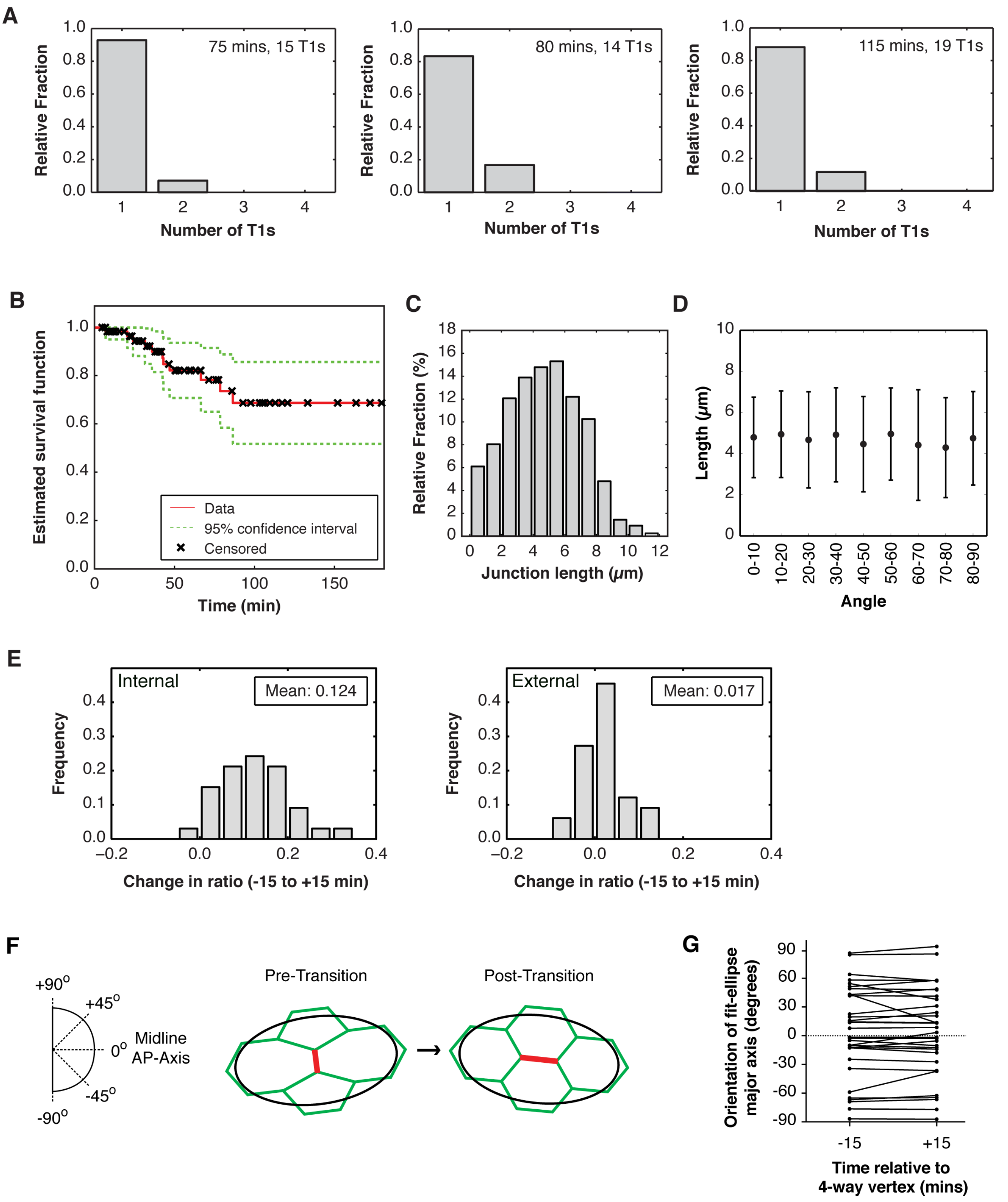

Figure S1. Related to Figure 1. Neighbour exchange events are reversible

(A), Bar charts showing the proportion of junctions that undergo unidirectional (one), bidirectional (two), and multidirectional (three or more) neighbour exchange events. n = 3 nota (not including nota from Figure 1C) over 75, 80, and 115 mins, respectively, labelled with DE-cadherin-GFP and imaged at 30 s intervals. **(B)**, Kaplan-Meier survival curve showing the probability that a neighbour exchange configuration persists for a given length of time. Events are 'censored' if the reverse transition has not occurred by the end of the movie.

The 95% confidence intervals are estimated using Greenwood's Formula. The probability of a configuration persisting for at least 150 min, along with the 95% confidence interval = 0.687 [0.5175, 0.8567]. **(C)**, Histogram of junction lengths in a single frame at 12h AP. n = 771 junctions / 3 nota. Average junction length = 4.69 ± 2.315µm. **(D)**, Junction lengths in a single frame at 12 h AP versus angle, with respect to the AP midline (0°). n = 771 junctions / 3 nota. **(E)**, Related to Figure 1H. Change in aspect ratio from t = -15 mins to t = +15 mins. A paired t-test was used to compare the aspect ratios at t = -15 mins and t= + 15 mins. Internally, the aspect ratio changes during a T1 event ($p < 0.0001$). Externally there is no change in aspect ratio ($p = 0.0502$). n = 33 exchange events from 4 flies. **(F)**, Diagram of a neighbour exchange event. The red junction represents the transition junction that is lost and gained. An ellipse (black) is fit to the four cells involved in the transition, and the feret angle of the ellipse is measured with respect to the midline (0°). **(G)**, The feret angle of fit ellipses are plotted at t=-15, and t=+15 mins, with paired results connected by a line. n = 33 exchange events from 4 flies.

Supplementary Figure 2

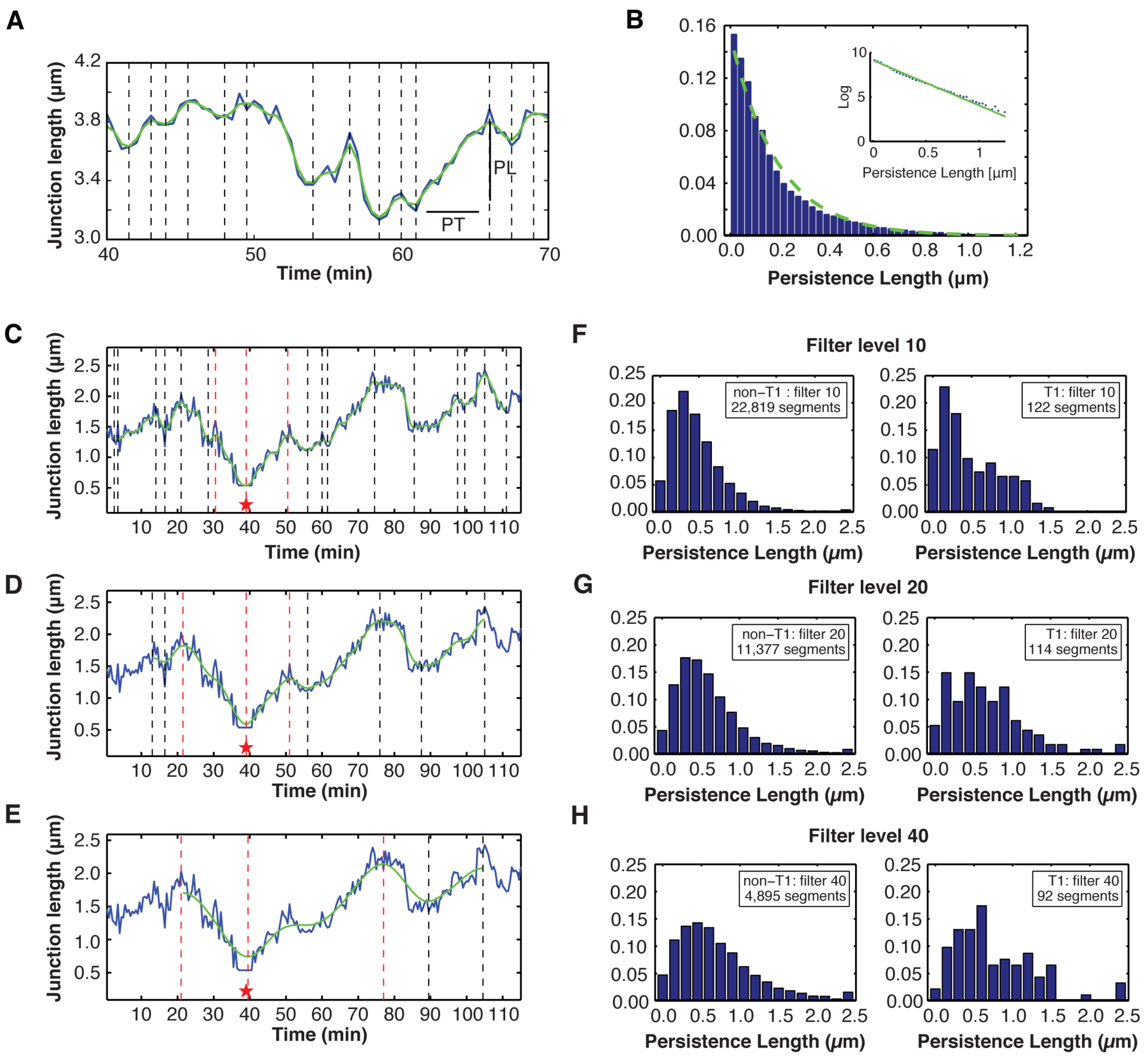

Figure S2. Related to Figure 2. Persistence length analyses results are consistent for a range of filter settings

(A), Time series line plot for a junction fluctuating in length. The data have been filtered using a moving average Hanning window, with a filter setting of 5, and split into segments where the junction is contracting or expanding. The persistence time (PT) and persistence length (PL) are defined as the duration of a segment and the change in junction length, respectively. **(B)**, Histogram of persistence lengths for a filter setting of 5. n = 60737 segments, 4 nota (green line). Best fit for an exponential function of the form $1/\tau\ exp(-t/\tau)$. The parameter is τ = 0.197 mm, with confidence bounds [0.1958 0.1989]. Inset, Number of segments in each bin of the histogram plotted on semi-log along with the exponential fit. **(C-E)**, Line plots of the length of a representative junction (blue) over time, for a junction that undergoes a neighbour exchange event at 40 min (marked by a red star). The time series has been split into segments where the junction length is monotonically increasing or decreasing. This was achieved by filtering the data using a moving average Hanning window

(green) - if the first derivative changes sign or is zero with opposite signs on either side of that point, a segment boundary is placed. Using a higher setting for the filter, results in fewer segments. The filter settings used are (**C**) 10, (**D**) 20 and (**E**) 40. The analysis is done using the raw data - the filter is only used to establish the position of the segment boundaries. The segments on either side of a T1 event (marked with red dotted lines) are defined as T1-segments. **(F-H)**, Persistence length distributions for (left) non-T1 and (right) T1 segments. The filter settings used are (**F**) 10, (**G**) 20, (**H**) 40, as shown in **C-E**. The number of segments included in each histogram are stated in the top right of each graph. Higher levels of filtering result in longer, and therefore fewer segments. The first and last segment of each time series is excluded from the analysis, regardless of whether a T1 event occurs (therefore the number of T1 segments included can vary with filter level). The distributions for T1 and non-T1 segments are compared used a two-sample Kolmogorov-Smirnov test. The p-values are listed in Table S1 and in every case the statistical test, at the 0.05 significance level, supports the null hypothesis that the samples are drawn from the same underlying distribution.

Supplementary Figure 3

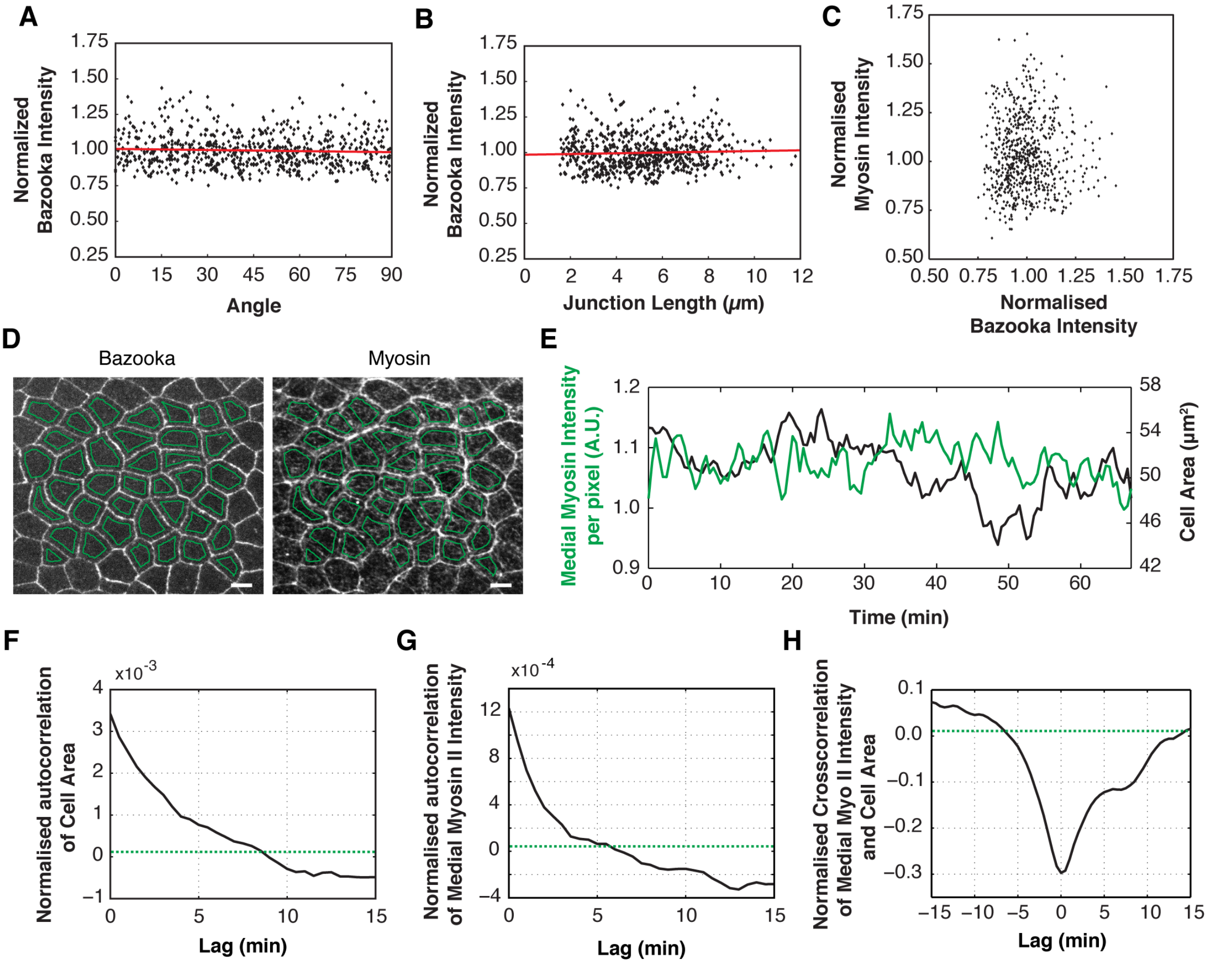

Figure S3. Related to Figure 4. Myosin and Bazooka intensities are uncorrelated and do not exhibit tissue-wide polarity. Cell areas and medial Myosin intensity do not display pulsatile behaviour and changes in medial Myosin intensity do not precede changes in cell area.

(A), Normalized junction intensities of Baz-mCh versus junction angle, as measured with respect to the AP midline (0°). Slope (with 95% confidence bounds) = -0.0003 (-0.00061 0.00011). Spearman's Rank = -0.048. n = 688 junctions / 3 nota. **(B)**, Normalized junction intensities of Baz-mCh versus junction length. Slope (with 95% confidence bounds) = 0.003 (-0.0019 0.0070). Spearman's Rank = 0.058. n = 688 junctions / 3 nota. **(C)**, Normalised (to mean intensity) paired junctional intensities of Baz and Myo-II show no correlation. Spearman's Rank = 0.024. n = 688 junctions / 3 nota. **(D)**, Apical surface projection of a live nota imaged with Bazooka-mCherry (left panel) and Spaghetti-Squash-GFP (right panel). Scale bar = 5μm. The medial region included in the analysis is outlined in green. **(E)**, A line plot showing a representative example of medial Myo-II-GFP (green) and cell area (blue) plotted as a function of time. Myo-II-GFP intensity is normalised to mean tissue intensity and the line plot shows the average Myo-II-GFP intensity per pixel. **(F)**, Mean autocorrelation for cell area variation of individual cells $\overline{\Delta A(t)\Delta A(t+\Delta t)}/(\bar{A})^2)$ as a function of lag time Δt. The autocorrelation is normalised by the time-averaged cell area A for each cell. n = 115 cells / 3 nota. **(G)**, Mean autocorrelation for medial Myo-II-GFP intensity in individual cells $\overline{\Delta I(t)\Delta I(t+\Delta t)}/(\bar{I})^2)$ as a function of lag time Δt. The autocorrelation is normalised by the time-averaged medial intensity for each cell. n = 115 cells / 3 nota. **(H)**,

Mean normalised crosscorrelation for medial Myosin intensity and cell area $\overline{\Delta I(t)\Delta A(t+\Delta t)}/\sigma^I\sigma^A$ as a function of lag time Δt, with σ^I and σ^A the intensity and cell area S.D. The minimum occurs at zero lag. The normalised crosscorrelation is calculated for each cell and then averaged over all cells in the analysis. n = 115 junctions / 3 nota, imaged at 30 s intervals for 60 mins.

Supplementary Figure 4

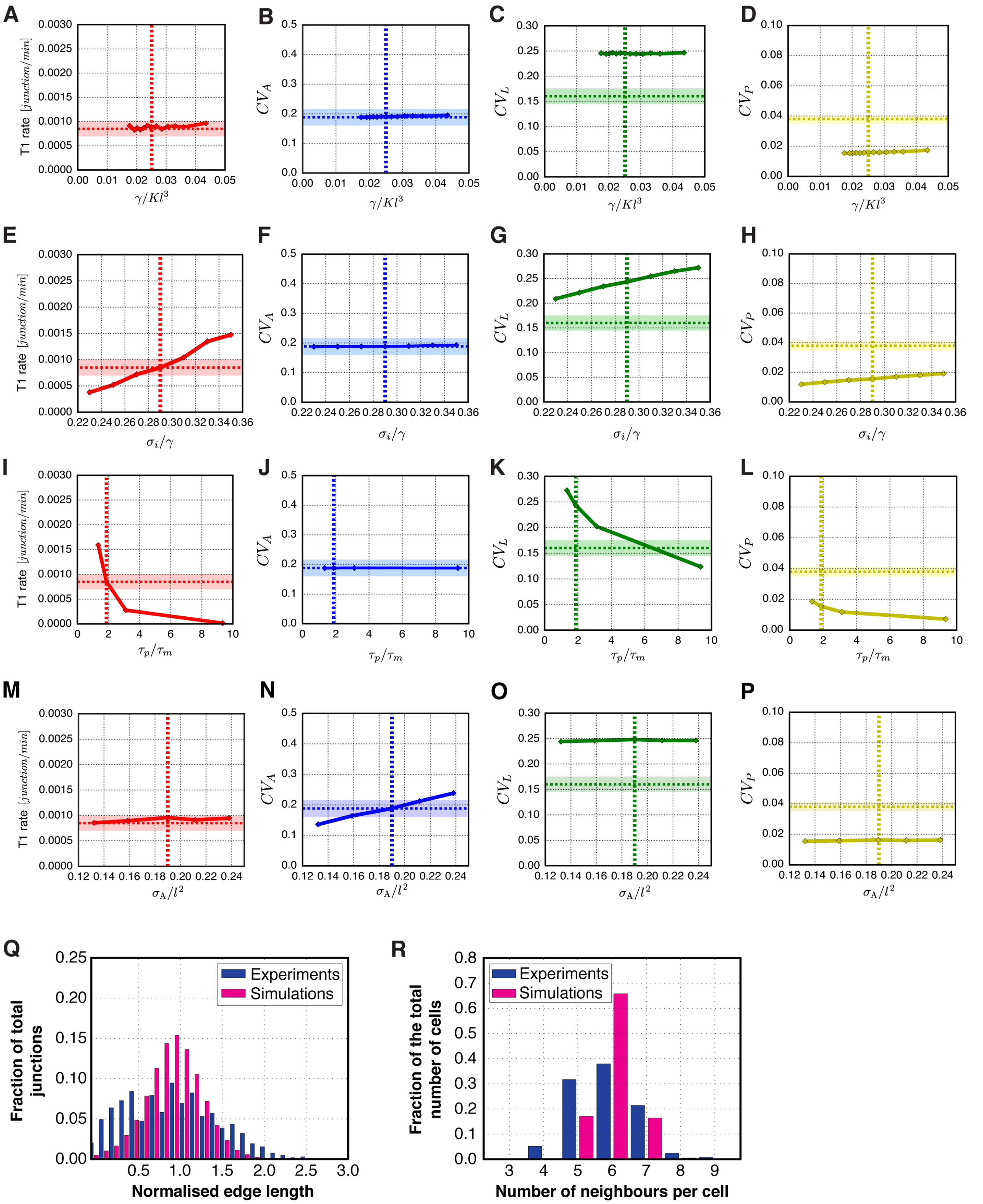

Figure S4. Related to Figure 5. 2D vertex model fitting parameters

(A-P) Plots showing the dependency of tissue properties on the fitting parameters. **(A, E, I, M)** T1 transition rate, **(B, F, J, N)** extrinsic area fluctuations (CV_A), **(C, G, K, O)** junction length intrinsic fluctuations (CV_L), and **(D, H, L, P)** relative perimeter fluctuations (CV_P), (dependency on the dimensionless model parameters: **(A-D)** dimensionless ratio of mean line tension over cell bulk elastic modulus (γ/Kl^3), **(E-H)** normalized intrinsic

noise intensity (σ_i/γ), **(I-L)** characteristic packing time (τ_p/τ_m) and **(M-P)** normalized standard deviation of preferred cell areas (σ_A/l^2). Dotted horizontal lines: experimental measurement, with shaded area indicating standard deviation. Dotted vertical lines: parameters used in wild-type simulations. **(Q)** Junction length distribution of simulations with wild type parameter settings to data obtained from experiments. **(R)** Polygon distribution of simulations with wild type parameter settings and data obtained from experiments.

Supplementary Figure 5

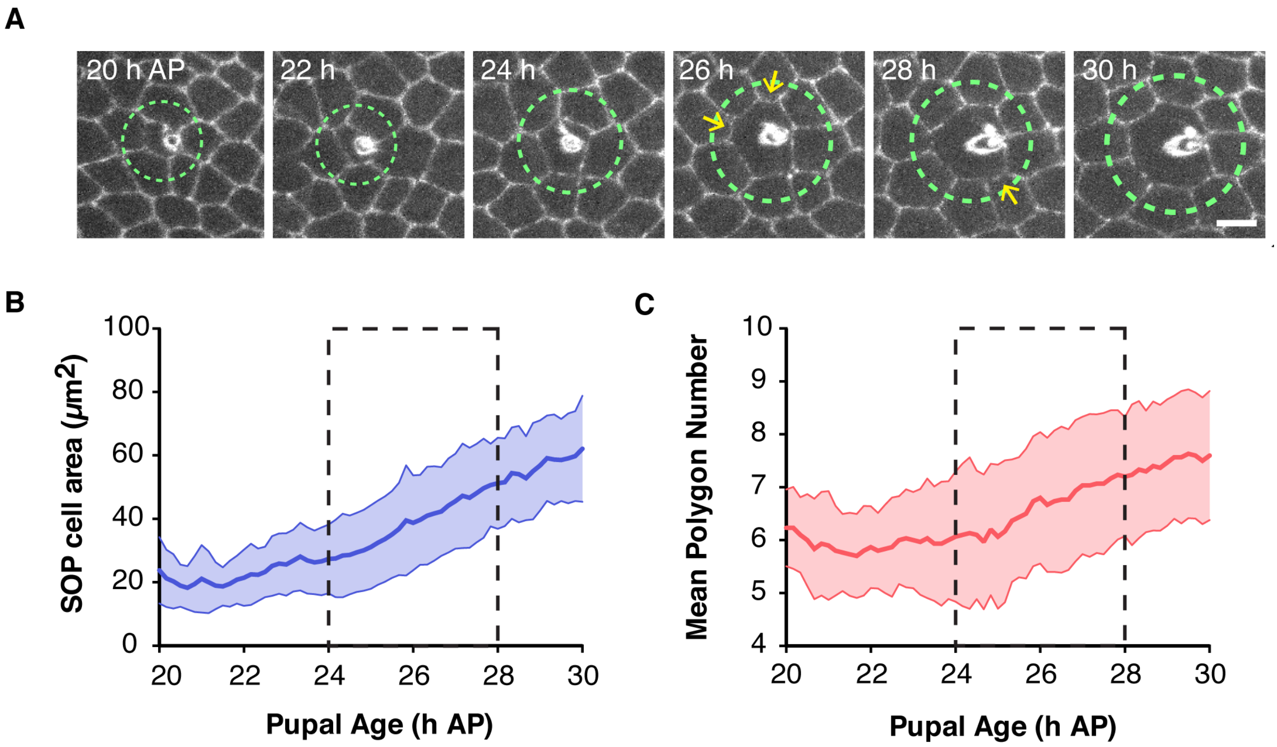

Figure S5. Related to Figure 6. Increasing neighbour exchange rates observed between 24 and 28 h AP are due to sensory organ precursor cell growth.

(A) Time-lapse montage of a representative SOP cell (circled by green dotted line), labeled with DE-cadherin-GFP, imaged from 20 to 30 h AP. Yellow arrows, label junctions that have been gained via neighbour exchange events. Scale bar, 5 μm. **(B-C)** Line plots showing the mean (with S.D) **(B)** SOP cell area and **(C)** the mean number of neighbours (polygon number) for SOP cells, from 20 – 30 h AP. Dotted box from 24-28 h AP indicates the time at which the T1 transition rate, as shown in Fig 6G, increases. n = 30 SOP cells / 3 nota.

Supplementary Figure 6

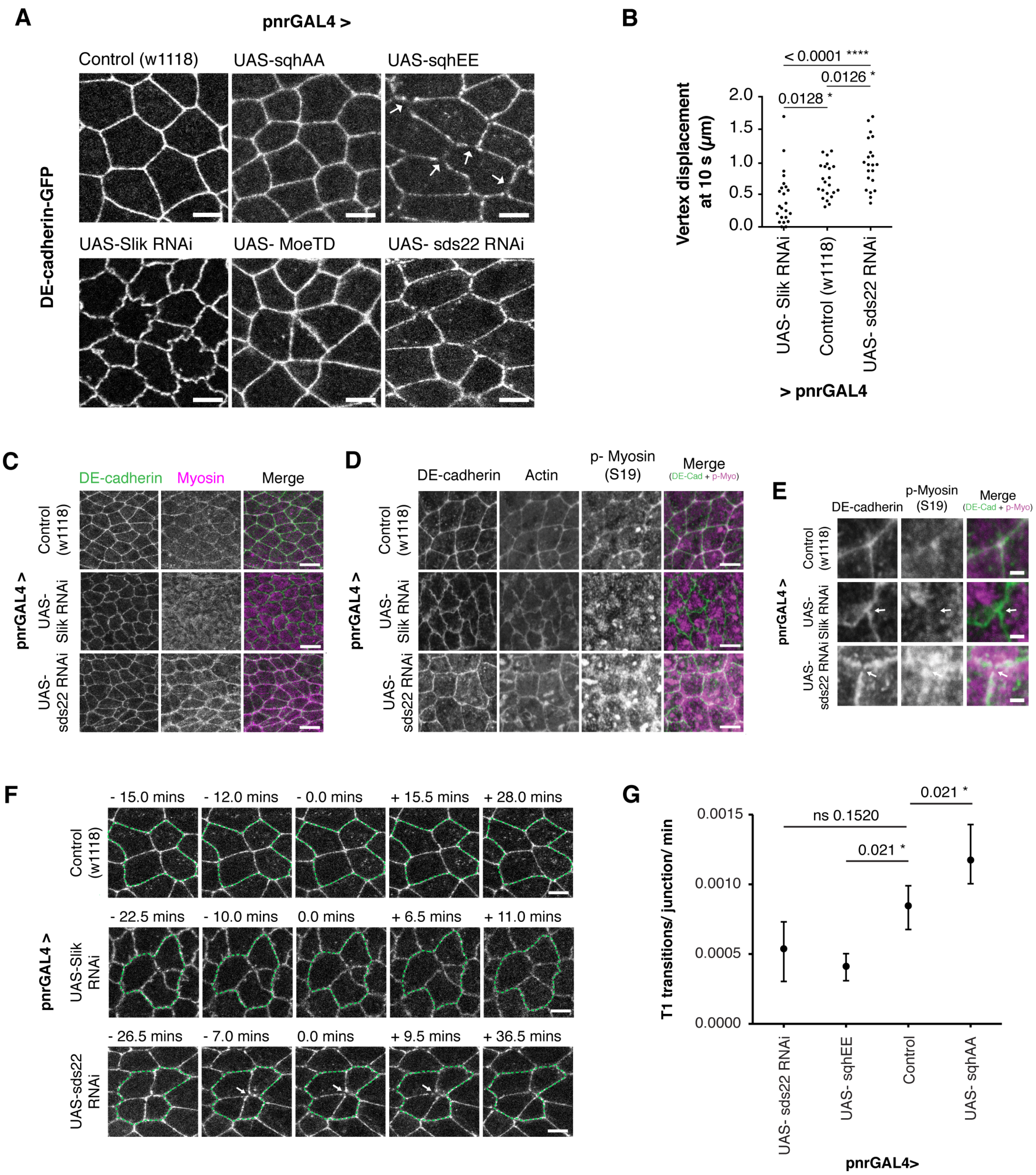

Figure S6. Related to Figure 7. Decreased Moesin activity causes a loss of junctional Myosin II and increases the medial pool. Phospho-null and phospho-active Myosin increase and decrease neighbour exchange rates, respectively.

(A) Apical surface projections of DE-cadherin-GFP labelled nota for control, UAS-sqhAA (Phospho-null Myosin II), UAS-sqhEE (Phospho-active Myosin II), UAS-Slik RNAi (decreased Moesin activity – note wiggly junction phenotype caused by pulling of medial Myosin on the junction), UAS-Moesin-TD (increased Moesin activity) and UAS-sds22 (increased Myosin and Moesin activity) driven by pnr-GAL4. Scale bar = 5μm. **(B)** Quantification of total vertex displacement at 10 s after laser dissection of single junctions in 12-13.5 h AP

pupae expressing Slik RNAi and sds22 RNAi. Dots indicate individual experiments, line represents median. 5-7 flies/condition. P-values calculated from unpaired t-tests. **(C)** Maximum surface projection images of nota live-imaged with ubi-DE-cadherin-GFP and Spaghetti-Squash-mCherry (MRLC) labelling total Myosin levels for control, Slik RNAi (note lack of Myosin on junctions) and sds22 RNAi (increased junctional Myosin). **(D)** Fixed-stain images for DE-cadherin-GFP (anti-GFP), F-actin (Phalloidin) and p-Myosin II (S19) for control, reduced Moesin activity (Slik RNAi) and increased Myosin and Moesin activity (sds22 RNAi). Scale bar = 5μm. **(E)** Increased zoom of vertices in **D**. Arrows for Slik RNAi highlight DE-cadherin junction, and absence of p-Myosin. Arrows for sds22 RNAI label junction break at 3-way vertex. Scale bar = 1μm. **(F)** Apical surface maximum projection montages of DE-cadherin-GFP labelled nota showing representative neighbour exchange events for control, sds22 RNAi and Slik RNAi. White arrows label junction breaks at the vertex during the sds22 RNAi transition. Slik RNAi nota can still undergo neighbour exchange in the absence of junctional Myosin – akin to Rok RNAi. **(G)** Quantification of normalised T1 transition rates for altered levels of Myosin activity. Dot indicates mean, tails show the data range. n = 3-4 flies / condition. P-values calculated from Kolmogorov-Smirnov tests.

Supplementary Figure 7

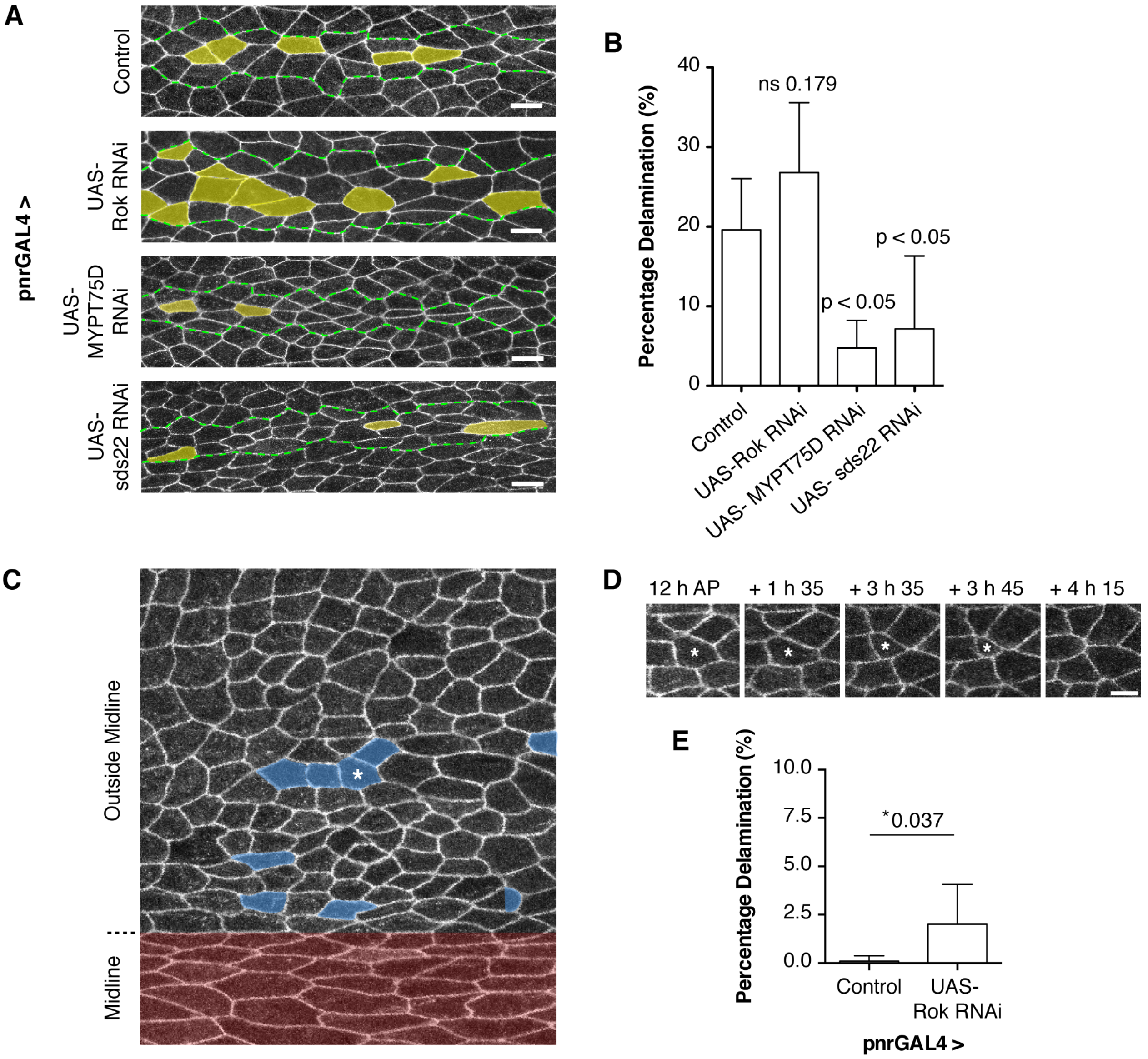

Figure S7. Related to Figure 7. A tissue-wide reduction in Myosin activity increases midline delamination levels and causes cells to delaminate outside of the midline.

(A) Apical surface maximum intensity projections of representative nota, labelled with ubi-E-cadherin-GFP, for altered Myosin II activity, showing the midline (dotted green line) with cells that delaminate (yellow) up to 18 h AP. Scale bar = 5μm. **(B)** Bar chart of percentage midline delamination for decreased (Rok RNAi) and increased (MYPT75-D RNAi and sds22 RNAi) Myosin II activity. Bar values indicate mean. P-values calculated from unpaired t-tests vs control. N = 4-6 nota per condition. **(C)** A representative notum with cells labelled outside of the midline that undergo basal delamination (blue). Midline region shaded red. Half blue cell indicates a daughter cell that delaminates after division. **(D)** Time-lapse montage of a cell (star labelled in **C**) that progressively loses junctions, and area, through time before extrusion. **(E)** Delamination levels were measured outside of the midline up to 18 h AP for control and Rok RNAi. Delamination levels were measured as a percentage of the total number of outer midline cells at 12 h AP. N = 6-7 flies with 110-237 (mean = 156) cells at 12 h AP. p-value calculated from a Mann-Whitney statistical test.

Supplementary Tables

Filter Level	P-value	Hypothesis
10	0.5142	H_0
15	0.2727	H_0
20	0.5294	H_0
25	0.2569	H_0
30	0.1081	H_0
35	0.1881	H_0
40	0.2572	H_0

Table S1. Related to Figure 2A-D and Figure S2.

The persistence length distributions for T1 and non-T1 segments are compared using a two-sample Kolmogorov-Smirnov test. The null hypothesis, H_0, is that the samples are drawn from the same underlying distribution. The analysis was carried out for seven different levels of filtering, between 10 and 40, and in every case the statistical test supports the null hypothesis at the 0.05 significance level. Note that, since we are testing a set of statistical inferences simultaneously, the appropriate significance level for the individual hypothesis tests is lower than the significance level for the set as a whole. Using the Bonferroni correction, we would get $\hat{}a = a/k$ = 0.05/7 = 0.007. P-values are all above 0.05.

Supplementary Movie Legends

Movie S1. Related to Figure 1.

This movie shows a region outside of the midline, of a wild-type developing pupal notum, visualised with DE-cadherin-GFP. The movie covers the period 12-13.5 h AP, prior to the onset of cell division and delamination, during which time cells undergo neighbour exchange (indicated in Figure 1), caused by fluctuations in junction length (indicated in Figure 2). Time interval between frames is 30 s and the video length is 1 h 30 mins. Scale bar, 10 μm.

Movie S2. Related to Figure 1.

This movie shows a four-cell cluster undergoing a uni-directional neighbour exchange event (indicated in Figure 1), visualised with DE-Cadherin-GFP. This is an event that occurs in Movie S1. Successive yellow arrows throughout the movie label the junction that is lost, the four-way vertex generated through junction loss, and the subsequent newly formed junction. Note neighbour exchange events also occur on the edge of the cluster throughout the movie. Time interval between frames is 30 s and the video length is 1 h 30 mins covering 12 - 13.5 h AP. Scale bar, 10 μm.

Movie S3. Related to Figure 5.

This movie shows three simulations of the 2D vertex model at low (0.8), control (1.0) and high (1.2) mean line tensions (γ/γ_0) (indicated in Figure 5). The edge colour, of each interface, corresponds to the level of line tension relative to it's mean (blue = low, red = high). Virtual time interval between frames is 15 s, with the video length 83 min 15 s.

Movie S4. Related to Figure 6.

This movie shows an example of a control laser ablation experiment (indicated in Figure 6B and 7C). Junctions outside of the midline are visualised with DE-cadherin-GFP between 12 and 13.5 h AP. The time interval between frames is 1 s with the laser ablation occurring at t = 0, indicated by a yellow star. The video length runs from 16 s pre-ablation to 120 s post-ablation. Scale bar, 5 μm.
