# Supplementary material for: Myosin II Controls Junction Fluctuations to Guide Epithelial Tissue Ordering
Source: Dev Cell. 2017 Nov 20;43(4):480–492.e6. doi: 10.1016/j.devcel.2017.09.018 (PMC5703647; doi:10.1016/j.devcel.2017.09.018)
Supplement: Methods S1. Supplemental Theory and 2D Vertex Model, Related to Figure S5 and STAR Methods [file mmc2.pdf]

METHODS S1 :
SUPPLEMENTARY THEORY AND 2D VERTEX MODEL.
RELATED TO FIGURE S5 AND STAR METHODS.

1. Fluctuating vertex model

1.1. **Data analysis.** We discuss here data analysis for a quantity $x_{ij}(t)$ measured over time on a cell junction connecting vertices i and j. The spatial and temporal averages are defined as follows:

$$\langle x \rangle (t) = \frac{1}{N_e} \sum_{ij} x_{ij}(t) \quad , \tag{1}$$

$$\overline{x}_{ij} = \frac{1}{N_t} \sum_{t} x_{ij}(t) \quad , \tag{2}$$

where junctions between vertices i and j are denoted by ij and different time points t are indicated by t, N_e is the number of junctions and N_t the number of time points. Additionally the deviation from the temporal mean is defined as:

$$\Delta x_{ij}(t) = x_{ij}(t) - \bar{x}_{ij} \quad . \tag{3}$$

and the standard deviation in time is defined as

$$\text{std}(x_{ij}) = \frac{1}{n} \sqrt{\sum_{t} (\Delta x_{ij}(t))^2} \quad . \tag{4}$$

where n is a normalisation factor that was taken equal to $\sqrt{N_t}$ for simulation analysis and $\sqrt{N_t - 1}$ for experimental data analysis. Those definitions are used to calculate the following quantities:

- **Figure 4E:** the mean normalized crosscorrelation between deviations in myosin intensity $\Delta I_{ij}^{\text{myosin}}(t)$ and junction length $\Delta l_{ij}(t)$

$$f_e(\Delta t) = \left\langle \overline{\frac{\left(\Delta I_{ij}^{\text{myosin}}(t) \Delta l_{ij}(t + \Delta t)\right)}{\sigma_{ij}^{I} \sigma_{ij}^{l}}} \right\rangle \quad , \tag{5}$$

at different lagtimes Δt, with the standard deviations of intensity and length over time on each junction defined as

$$\sigma_{ij}^{I\,2} = \frac{1}{N_t - 1} \sum_t \left(I_{ij} - \overline{I_{ij}}\right)^2 \quad , \tag{6}$$

$$\sigma_{ij}^{l\,2} = \frac{1}{N_t - 1} \sum_t \left(l_{ij} - \overline{l_{ij}}\right)^2 \quad . \tag{7}$$

In Eq. 5, the time average is performed according to the number of time points available for each time lag Δt.

- **Figure 5B left:** histogram showing the distribution of temporal averaged mean myosin intensities $\bar{I}_{ij}^{\text{myosin}}$ at different junctions ij,
- **Figure 5C:** the mean autocorrelation of deviations from the mean myosin intensity at junctions

$$a(\Delta t) = \left\langle \frac{\overline{I_{ij}^{\text{myosin}}(t) I_{ij}^{\text{myosin}}(t + \Delta t))} - \bar{I}_{ij}^{\text{myosin}\,2}}{\bar{I}_{ij}^{\text{myosin}\,2}} \right\rangle \quad , \tag{8}$$

  at different lagtimes Δt,
- **Figure 5F:** the standard deviation of number of neighbours per cell within the tissue at time t

$$\sigma_N(t) = \sqrt{\frac{1}{N_{\text{cells}}} \sum_\beta \left(N^\beta_{\text{neighbours}}(t) - \frac{1}{N_{\text{cells}}} \sum_\delta N^\delta_{\text{neighbours}}(t)\right)^2} \quad , \tag{9}$$

  as a measurement for topological disorder, and where β, δ label cells in the tissue, N_{cells} the total number of cells and $N^\beta_{\text{neighbours}}(t)$ the number of neighbours of cell β at time t,
- **Figure 5H:** the mean normalized crosscorrelation between deviations in line tension $\Delta\gamma_{ij}(t)$ and junction length calculated in simulations

$$f_t(\Delta t) = \left\langle \frac{\overline{\Delta\gamma_{ij}(t)\Delta l_{ij}(t + \Delta t)}}{\sigma^\gamma_{ij}\sigma^l_{ij}} \right\rangle \quad , \tag{10}$$

  at different lagtimes Δt, with the length standard deviation defined as in Eq. 7, and the tension standard deviation defined as

$$\sigma_{ij}^{\gamma\,2} = \frac{1}{N_t - 1} \sum_t \left(\gamma_{ij} - \overline{\gamma_{ij}}\right)^2 \quad . \tag{11}$$

In Eq. 10, the time average is performed according to the number of time points available for each time lag Δt.

- **Figure 5I:** The coefficient of variation of junction lengths $\mathrm{CV_L}$ is calculated according to Eq. 26.
- **Figure 6E:** Coefficient of variation of junction lengths $\mathrm{CV_{Le}}$ across the tissue at different times t defined as

$$\mathrm{CV_{Le}}(t) = \frac{\sqrt{\langle (l_{ij}(t) - \langle l_{ij}\rangle(t))^2\rangle}}{\langle l_{ij}\rangle(t)} \quad . \tag{12}$$

1.2. **General equations.** We describe here the properties of the fluctuating vertex model. The position of vertex i in the packing is denoted $\mathbf{x}_i$. The equation of motion of a vertex is written

$$\alpha \frac{d\mathbf{x}_i}{dt} = \mathbf{f}_i \tag{13}$$

where $\mathbf{f}_i$ is the force acting on vertex i and global friction coefficient α. Forces on vertices are obtained by differentiation of a virtual work δW:

$$\mathbf{f}_i = -\frac{\delta W}{\delta \mathbf{x}_i} \tag{14}$$

where the virtual work is taken to be

$$\delta W = \sum_{\langle i,j\rangle} \gamma_{ij}\delta l_{ij} + \sum_{\beta} K(A_\beta - \bar{A})\delta A_\beta \tag{15}$$

where γ_{ij} and l_{ij} are the line tension and length of the oriented junction joining vertices i to j, A_β is the area of cell β, $\bar{A}$ is a target area, and K is an area elastic modulus. The sum over edges $\langle i,j\rangle$ is taken over oriented edges such that edges are not counted twice. We consider the total area A of the packing fixed. The preferred area of each cell $\bar{A}$ is taken from a random gaussian distribution:

$$P(\bar{A}) = \frac{1}{\sqrt{2\pi}\sigma_A} e^{-\frac{\left(\bar{A}^0 - \bar{A}\right)^2}{2\sigma_\mathrm{A}{}^2}} \tag{16}$$

Because the overall size of the system is fixed, the value of $\bar{A}^0$ does not influence here simulation results. Simulations are started with N cells. We denote $l = \sqrt{A/N}$ a characteristic length of the model. In the model we propose, the line tensions γ_{ij} are fluctuating

in time according to

$$\frac{d\gamma_{ij}}{dt} = -\frac{1}{\tau_m}(\gamma_{ij} - \gamma_{ij}^0) + \xi_{ij}(t) \quad , \tag{17}$$

with $\xi_{ij}(t)$ a white, uncorrelated noise:

$$\langle \xi_{ij}(t) \rangle = 0 \tag{18}$$

$$\langle \xi_{ij}(t)\xi_{kl}(t') \rangle = \frac{2{\sigma_\mathrm{i}}^2}{\tau_m}\delta_{ik}\delta_{jl}\delta(t-t'). \tag{19}$$

In addition, the line tension γ_{ij} are set to 0 when the equation of evolution 17 above yields a negative value. The line tension deviation of newly created edges is chosen from a normal distribution, $\gamma_{ij} - \gamma_{ij}^0 \sim \mathcal{N}(0, \sigma_i^2)$. In Eq. 17, we have introduced a line tension correlation time τ_m, a reference line tension γ_{ij}^0, and an intrinsic tension deviation, σ_i. In the following, we have chosen σ_i to be a constant independent of the junction.

The target line tensions γ_{ij}^0 are chosen for different junctions based on the following rule: taking β_1 and β_2 the cells on each side on the junctions, we take

$$\gamma_{ij}^0 = \frac{1}{2}\left(\gamma_{\beta_1} + \gamma_{\beta_2}\right) \tag{20}$$

where the value of line tension per cell $\gamma_\beta > 0$ is taken from the truncated gaussian distribution $P\left(\gamma_\beta^0\right)$:

$$P\left(\gamma_\beta\right) = \frac{1}{n_\sigma} e^{-\frac{\left(\gamma_\beta - \gamma\right)^2}{2{\sigma_\mathrm{e}}^2}}, \quad \gamma_\beta > 0, \tag{21}$$

with $n_\sigma = \int_0^\infty d\gamma_\beta \exp\left(-\left(\gamma_\beta - \gamma\right)^2/2/\sigma_\mathrm{e}^2\right)$ a normalisation constant. Note that in the main text we refer for simplicity to the parameter γ as the mean line tension, as it is the mean line tension of the gaussian distribution prior to truncation. The actual mean line tension of the packing is higher however due to truncation of negative values in the probability distribution of Eq. 21.

A characteristic mechanical time of the packing can be defined from the friction coefficient α, the reference line tension γ and the length l:

$$\tau_p = \frac{\alpha l}{\gamma} \tag{22}$$

1.3. **Model implementation.** To simulate Eq. 17, we use the following discretisation with time step Δt:

$$\gamma_{ij}(t+\Delta t) = \gamma_{ij}(t) - \frac{\Delta t}{\tau_m}\left(\gamma_{ij}(t) - \gamma_{ij}^0\right) + \sqrt{\frac{2\sigma_{\rm i}^2 \Delta t}{\tau_m}}\bar{\xi}_{ij}(t) \tag{23}$$

with the definition of the discrete noise term $\xi(t)$:

$$\bar{\xi}_{ij}(t) = \sqrt{\frac{\tau_m}{2\Delta t \sigma_{\rm i}^2}} \int_t^{t+\Delta t} {\rm d}s \xi_{ij}(s) \tag{24}$$

One can verify from Eq. 18-19 that $\langle \bar{\xi}_{ij}(t) \rangle = 0$ and $\langle \bar{\xi}_{ij}(t) \bar{\xi}_{kl}(t) \rangle = \delta_{ik}\delta_{jl}$. For each junction and time point, $\bar{\xi}_{ij}(t)$ is therefore taken out of a Gaussian distribution with probability density:

$$P(\bar{\xi}_{ij}) = \frac{1}{\sqrt{2\pi}} e^{-\frac{\bar{\xi}_{ij}^2}{2}} \tag{25}$$

In addition, the line tension γ_{ij} are set to 0 when the equation of evolution above yields a negative value. In simulations, two three-fold vertices are merged in a 4-fold vertex when the junction length l_{ij} falls below a threshold l^*, chosen to be much smaller than the reference length l (see Table 1).

To test if the formation of a new junction joining two cells connected by a 4-fold vertex is favorable, a small junction with length $1.5l^*$ is created in between the two cells, a new tension value is allocated to the new junction, and the new junction with length l_e is maintained if $dl_e/dt > 0$.

Simulations are performed on a periodic square domain with a time step $\Delta t/\tau_m \simeq 8.8 \cdot 10^{-4}$, and resolution of 4-fold vertices is tested every 10 iterations.

1.4. **Comparison to experimental data.** To adjust the model to experimental data, we set the timescale τ_m to be equal to experimental measurement of the myosin turnover time (Table 1). The parameter ratio $\sigma_{\rm e}/\sigma_{\rm i}$ is fixed to 3.7, which we found to yield values of extrinsic to intrinsic deviation of line tension to be close to the experimentally measured ratio of extrinsic and intrinsic deviation of myosin intensities (see Fig. 5B). The remaining adimensional parameters to adjust are τ_p/τ_m, $\gamma/(Kl^3)$, and the noise strengths σ_A/l^2 and $\sigma_{\rm i}/\gamma$ (Table 1). $\gamma/(Kl^3)$ was chosen small enough to ensure a minimal amount of

cell delamination in simulations. In order to perform additional adjustment, for each parameter set, the following measurements were obtained:

- the relative junction length fluctuations $\mathrm{CV_L} = f\left(\frac{\tau_p}{\tau_m}, \frac{\gamma}{Kl^3}, \frac{\sigma_A}{l^2}, \frac{\sigma_\mathrm{i}}{\gamma}\right)$, calculated as

$$\mathrm{CV_L} = \frac{1}{\sum_{i,j} T_{ij}} \sum_{i,j} \frac{\mathrm{std}(l_{ij})}{\overline{l}_{ij}} T_{ij} \tag{26}$$

  where T_{ij} denotes the time interval during which the length of the junction ij is measured,

- the relative perimeter fluctuations $\mathrm{CV_P} = g\left(\frac{\tau_p}{\tau_m}, \frac{\gamma}{Kl^3}, \frac{\sigma_A}{l^2}, \frac{\sigma_\mathrm{i}}{\gamma}\right)$, calculated as

$$\mathrm{CV_P} = \frac{1}{N_\mathrm{cells}} \sum_{\beta} \frac{\mathrm{std}(P_\beta)}{\overline{P}_\beta} \tag{27}$$

  with P_β the perimeter of cell β, and N_cells denotes the total number of cells.

- the coefficient of variation of average areas $\mathrm{CV_A} = h\left(\frac{\tau_p}{\tau_m}, \frac{\gamma}{Kl^3}, \frac{\sigma_A}{l^2}, \frac{\sigma_\mathrm{i}}{\gamma}\right)$, calculated as

$$\mathrm{CV_A} = \frac{1}{\sqrt{N_\mathrm{cells}}} \frac{\sqrt{\sum_\beta (\overline{A}_\beta - \langle \overline{A} \rangle)^2}}{\langle \overline{A} \rangle} \tag{28}$$

  where the sum is performed over cells β, $\overline{A}_\beta$ is the time-averaged area of cell β, $\langle \overline{A} \rangle = \frac{1}{N_\mathrm{cells}} \sum_\beta \overline{A}_\beta$ the average cell area, and N_cells the total number of cells.

- the T_1 rate $r = k\left(\frac{\tau_p}{\tau_m}, \frac{\gamma}{Kl^3}, \frac{\sigma_A}{l^2}, \frac{\sigma_\mathrm{i}}{\gamma}\right)$. To calculate the rate of transition, we note that experimental resolution limits the length below which vertices and junctions can be distinguished. In order to define T_1 events, we therefore use a definition for simulated packings involving a threshold junction length l_t. When a junction length shrinks below the threshold length l_t, its end vertices are seen as part of a single vertex. We define the following sequence of events as one T_1 event:

  (1) The length of a junction i, j l_{ij} decreases below a threshold l_t, $l_{ij} < l_t$.

  (2) a new junction k, l with length $l_{kl} > l_t$ appears between two cells which were formerly not connected and were nearest neighbours of i and j at the time of the disappearance of the junction i, j.

  Note that this definition excludes some rare 5-fold vertex formation event from the counting. Based on estimates of experimental resolution, we chose here $l_t/\langle l \rangle \simeq 0.05$ with $\langle l \rangle$ the mean junction length, and chose l^* such that $l^* < l_t$.

- the lagtime of the minimum position of the crosscorrelation of junction lengths and line tensions $m = h\left(\frac{\tau_p}{\tau_m}, \frac{\gamma}{Kl^3}, \frac{\sigma_A}{l^2}, \frac{\sigma_{\rm i}}{\gamma}\right)$.

We initiate the simulation by performing a Voronoi tessellation with 793 cells on a quadratic periodic box with side lengths $L/l = \sqrt{793} \simeq 28.16$. The tissue is then relaxed during a simulation time corresponding to $\Delta t \simeq 469\tau_p$. Topological transitions are then quantified during a simulation time $\Delta t \simeq 19\tau_p$.

In order to find fitting parameters, we define an objective function as:

$$S = \sqrt{(\mathrm{Div}(\mathrm{CV_L}))^2 + (\mathrm{Div}(\mathrm{CV_P}))^2 + (\mathrm{Div}(\mathrm{r}))^2 + (\mathrm{Div}(\mathrm{m}))^2 + (\mathrm{Div}(\mathrm{CV_A}))^2} \tag{29}$$

with

$$\mathrm{Div}(\mathrm{X}) = \frac{\mathrm{X}^{\mathrm{exp}} - \mathrm{X}^{\mathrm{sim}}}{\mathrm{X}^{\mathrm{exp}}} \tag{30}$$

To obtain the best fit between model and experiments, we find the minimum value of the objective function over a range of tested values of the dimensionless model parameters τ_p/τ_m, $\gamma/(Kl^3)$, σ_A/l^2 and $\sigma_{\rm i}/\gamma$. The best fit parameters are listed in Table 1 and the corresponding fitted values are listed in Table 2.

Model Parameter	
τ_m	2 min 22 sec
$\sigma_{\rm e}/\sigma_{\rm i}$	3.7
τ_p/τ_m	1.87
$\gamma/(Kl^3)$	0.025
$\sigma_{\rm i}/\gamma$	0.29
σ_A/l^2	0.19
l^*/l	0.0094

Table 1. Dimensionless model parameters

Measurement	Model	Experiment
r [10^{-3}/junction/min]	0.89 ± 0.15	0.85 ± 0.15
$\mathrm{CV_L}$	0.246 ± 0.004	0.1602 ± 0.0147
$\mathrm{CV_P}$	0.0158 ± 0.0005	0.0381 ± 0.0029
$\mathrm{CV_A}$	0.189 ± 0.001	0.1882 ± 0.0275
m	$\sim$ 1min	$\sim$ 1min

Table 2. Measurements used to adjust model to experiments. The accuracy in the determination of m is limited by time resolution of experiments.

2. Simplified description of junction fluctuation

We now discuss an approximate estimate of the variance of junction lengths in the packing as a function of the model parameters. In this approximate description, we consider a junction joining vertices i and j with length l_{ij}. We consider an equilibrium state of the tissue where $\gamma_{ij} = \gamma_{ij}^0$ and $l_{ij} = l_{ij}^0$, and define deviations of the junction length from equilibrium by $\Delta l_{ij} = l_{ij} - l_{ij}^0$ and junction tension by $\Delta\gamma_{ij} = \gamma_{ij} - \gamma_{ij}^0$. We consider here that deviations of the junction length from equilibrium are resisted by an effective spring modulus k_{eff}, such that the equation for the junction length deviation reads

$$\alpha \frac{d\Delta l_{ij}}{dt} = -k_{\mathrm{eff}} \Delta l_{ij} - 2\Delta\gamma_{ij}, \tag{31}$$

We denote $\tau_e = \alpha/k_{\mathrm{eff}}$ the characteristic mechanical time, playing a similar role than the time τ_p introduced above. Following Eq. 17, we write that the junction line tension varies according to

$$\frac{d\Delta\gamma_{ij}}{dt} = -\frac{1}{\tau_m}\Delta\gamma_{ij} + \xi_{ij}(t). \tag{32}$$

with

$$\langle \xi_{ij}(t) \rangle = 0 \tag{33}$$

$$\langle \xi_{ij}(t)\xi_{ij}(t') \rangle = \frac{2{\sigma_{\mathrm{i}}}^2}{\tau_m}\delta(t-t') \tag{34}$$

Defining the vector $\mathbf{x} = (\Delta l_{ij}, \Delta\gamma_{ij})$, the combined equation for the junction length and tension can be written

$$\frac{\mathrm{d}}{\mathrm{d}t}\mathbf{x} = \mathbf{M}\mathbf{x} + \boldsymbol{\xi} \tag{35}$$

with the definitions

$$\mathbf{M} = \begin{pmatrix} -\frac{1}{\tau_e} & -\frac{2}{k_{\text{eff}}\tau_e} \\ 0 & -\frac{1}{\tau_m} \end{pmatrix}, \quad \boldsymbol{\xi} = \begin{pmatrix} 0 \\ \xi_{ij}(t) \end{pmatrix} \tag{36}$$

To solve for Eq. 35, we note that it can be rewritten

$$\frac{\mathrm{d}}{\mathrm{d}t}\mathbf{x}^* = \mathbf{D}\mathbf{x}^* + \mathbf{T}^{-1}\boldsymbol{\xi} \tag{37}$$

where we have introduced

$$\mathbf{T} = \begin{pmatrix} \frac{2\tau_m}{k_{\text{eff}}(\tau_e-\tau_m)} & 1 \\ 1 & 0 \end{pmatrix}, \quad \mathbf{x} = \mathbf{T}\mathbf{x}^*, \quad \mathbf{D} = \mathbf{T}^{-1}\mathbf{M}\mathbf{T} = \begin{pmatrix} -\frac{1}{\tau_m} & 0 \\ 0 & -\frac{1}{\tau_e} \end{pmatrix}. \tag{38}$$

Solving equation (37) leads to the following expression for the correlation functions of junction lengths and tensions at steady-state ($t \to \infty$):

$$\langle \Delta\gamma_{ij}(t)\Delta\gamma_{ij}(t+\Delta t)\rangle = {\sigma_{\text{i}}}^2 \exp\left(-\frac{|\Delta t|}{\tau_m}\right) \tag{39}$$

$$\langle \Delta l_{ij}(t)\Delta l_{ij}(t+\Delta t)\rangle = \frac{4{\sigma_{\text{i}}}^2\tau_m}{k_{\text{eff}}^2\left(\tau_e^2-\tau_m^2\right)}\left[-\tau_m \exp\left(-\frac{|\Delta t|}{\tau_m}\right) + \tau_e \exp\left(-\frac{|\Delta t|}{\tau_e}\right)\right] \tag{40}$$

$$\langle \Delta\gamma_{ij}(t)\Delta l_{ij}(t+\Delta t)\rangle = \begin{cases} -\frac{2\tau_m{\sigma_{\text{i}}}^2}{k_{\text{eff}}(\tau_e+\tau_m)}\exp\left(\frac{\Delta t}{\tau_m}\right) & ;\ \text{for } \Delta t < 0 \\ \frac{4{\sigma_{\text{i}}}^2}{k_{\text{eff}}(\tau_e-\tau_m)}\left[\frac{\tau_m}{2}\exp\left(-\frac{\Delta t}{\tau_m}\right) - \frac{\tau_m\tau_e}{\tau_m+\tau_e}\exp\left(-\frac{\Delta t}{\tau_e}\right)\right] & ;\ \text{for } \Delta t > 0 \end{cases} \tag{41}$$

Using this last relation, we find that the minimum of the cross correlation function $\langle \Delta\gamma_{ij}(t)\Delta l_{ij}(t+\Delta t)\rangle$ reads

$$m = \frac{\tau_e\tau_m}{\tau_e-\tau_m}\log\left(\frac{\tau_m+\tau_e}{2\tau_m}\right), \tag{42}$$

and the variance of junction lengths fluctuations reads:

$$\langle \Delta {l_{ij}}^2\rangle = \frac{4{\sigma_{\text{i}}}^2\tau_m}{k_{\text{eff}}^2(\tau_e+\tau_m)}. \tag{43}$$

From the expressions above, we draw the following conclusions:

- the autocorrelation of junction tension decays exponentially with lag time with a time scale τ_m (Eq. 39).
- The minimum of the cross-correlation m/τ_m only depends on the ratio τ_e/τ_m.
- The strength of junction length fluctuations $\langle \Delta l_{ij}{}^2 \rangle$ depends on the normalised strength of tension fluctuations $\sigma_i/k_{\mathrm{eff}}$.

Based on this analysis, we constrained these parameters in our simulations to reproduce experimental results (Eq. 29), in addition to the rate of topological transitions and the variation of cell perimeter and area.
