# Supplementary material for: Myosin II Controls Junction Fluctuations to Guide Epithelial Tissue Ordering
Source: Dev Cell. 2017 Nov 20;43(4):480–492.e6. doi: 10.1016/j.devcel.2017.09.018 (PMC5703647; doi:10.1016/j.devcel.2017.09.018)
Supplement: Document S2. Article plus Supplemental Information [file mmc7.pdf]

Article

Developmental Cell

Myosin II Controls Junction Fluctuations to Guide Epithelial Tissue Ordering

Graphical Abstract

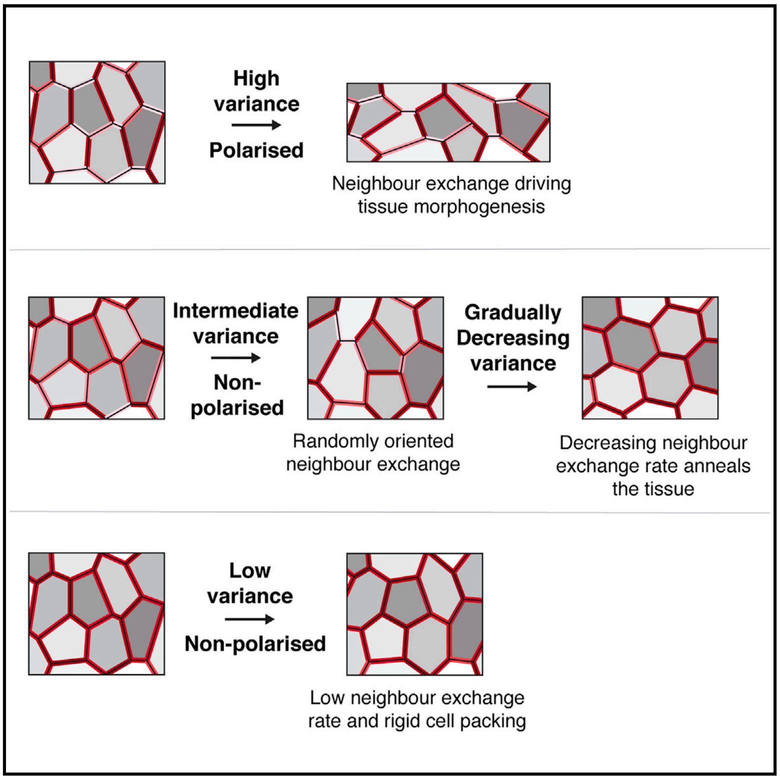

Highlights

- Fluctuations in junction length cause neighbor exchange events without morphogenesis
- The variance in junction tension determines how actomyosin influences T1 rates
- Globally increasing junctional actomyosin levels inhibits neighbor exchange
- A developmental increase in isotropic junction tension refines cellular packing

Authors

Scott Curran, Charlotte Strandkvist, Jasper Bathmann, Marc de Gennes, Alexandre Kabla, Guillaume Salbreux, Buzz Baum

Correspondence

b.baum@ucl.ac.uk (B.B.),
guillaume.salbreux@crick.ac.uk (G.S.)

In Brief

Curran et al. investigate adherens junction remodeling in the fly notum, where differences in actomyosin levels drive fluctuations in junction length and neighbor exchange, but not morphogenesis. A developmental increase in global junctional tension reduces, rather than drives, neighbor exchange to promote tissue ordering.

Curran et al., 2017, Developmental Cell 43, 480–492
November 20, 2017 © 2017 Published by Elsevier Inc.
https://doi.org/10.1016/j.devcel.2017.09.018

CellPress

Developmental Cell
Article

Myosin II Controls Junction Fluctuations to Guide Epithelial Tissue Ordering

Scott Curran,[1,5] Charlotte Strandkvist,[1,5] Jasper Bathmann,[2,6] Marc de Gennes,[2,6] Alexandre Kabla,[3] Guillaume Salbreux,[2,4,7,*] and Buzz Baum[1,4,7,8,*]
[1]Medical Research Council - Laboratory of Molecular Cell Biology, University College London, Gower Street, London WC1E 6BT, UK
[2]The Francis Crick Institute, 1 Midland Road, London NW1 1AT, UK
[3]Department of Engineering, University of Cambridge, Cambridge CB2 OQH, UK
[4]Institute for the Physics of Living Systems, University College London, Gower Street, London WC1E 6BT, UK
[5]These authors contributed equally
[6]These authors contributed equally
[7]Senior author
[8]Lead Contact
*Correspondence: b.baum@ucl.ac.uk (B.B.), guillaume.salbreux@crick.ac.uk (G.S.)
https://doi.org/10.1016/j.devcel.2017.09.018

SUMMARY

Under conditions of homeostasis, dynamic changes in the length of individual adherens junctions (AJs) provide epithelia with the fluidity required to maintain tissue integrity in the face of intrinsic and extrinsic forces. While the contribution of AJ remodeling to developmental morphogenesis has been intensively studied, less is known about AJ dynamics in other circumstances. Here, we study AJ dynamics in an epithelium that undergoes a gradual increase in packing order, without concomitant large-scale changes in tissue size or shape. We find that neighbor exchange events are driven by stochastic fluctuations in junction length, regulated in part by junctional actomyosin. In this context, the developmental increase of isotropic junctional actomyosin reduces the rate of neighbor exchange, contributing to tissue order. We propose a model in which the local variance in tension between junctions determines whether actomyosin-based forces will inhibit or drive the topological transitions that either refine or deform a tissue.

INTRODUCTION

Epithelia play an important role as selective barriers that separate animal tissues from the external environment. This depends upon the presence of linear adhesive contacts, called adherens junctions (AJs), which bind neighboring cells to one another (Harris and Tepass, 2010; van Roy and Berx, 2008; Tepass et al., 2001). Since epithelia must tolerate changes in cell packing, even during periods of homeostasis, it is important that the loss and gain of AJs occurs without compromising tissue integrity. This requires that AJs be dynamic structures.

In a monolayer epithelium, the loss and birth of AJs follows a characteristic trajectory as cells change neighbors. First, an adhesive contact connecting two neighboring epithelial cells is lost, leading to the formation of a four-way vertex. This is then resolved by the birth and elongation of a new AJ interface at $\sim 90^\circ$ to the first. This simple process, often called a T1 transition, connects the two cells in a quartet that were previously separate from one another (Guillot and Lecuit, 2013; Nishimura and Takeichi, 2009; Takeichi, 2014). Such topological transitions provide epithelial monolayers with the fluidity necessary to preserve tissue integrity in the face of the disruptive influence of epithelial cell division (Gibson et al., 2006) and cell delamination (Marinari et al., 2012); while also allowing packing irregularities and defects in the tissue to be resolved (Classen et al., 2005; Farhadifar et al., 2007). Moreover, when accompanied by a redistribution of cell mass, directed neighbor exchange events can be used to drive large-scale morphogenetic movements (Fristrom, 1988; Heisenberg and Bellaiche, 2013).

In many systems, the forces required to drive T1 transitions are generated by the molecular motor, non-muscle Myosin II, as it acts on AJ-associated actin filaments (Desai et al., 2013; Lecuit and Lenne, 2007). Through the action of Myosin II, the sliding of anti-parallel filaments, coupled to the AJ, generates localized mechanical tension that causes AJs to shorten, thereby triggering neighbor exchange. This has been especially well studied in the developing *Drosophila* germband, where polarized junctional actomyosin (Simoes Sde et al., 2010), actomyosin flows (Bertet et al., 2004; Rauzi et al., 2010), together with the destabilization of E-cadherin at dorsal-ventral AJs (Tamada et al., 2012) drives tissue elongation (Blankenship et al., 2006; Irvine and Wieschaus, 1994; Simoes Sde et al., 2010). Nevertheless, the impact of actomyosin-based forces on individual AJs and on the tissue as a whole critically depends on the precise localization and polarity of the actomyosin network. Thus, while a pulsed polarized actomyosin network drives neighbor exchange (Zallen and Blankenship, 2008; Zallen and Wieschaus, 2004), medial actomyosin pulses tend to induce apical cell constriction, as seen during ventral furrow invagination (Martin et al., 2009; Mason et al., 2013; Vasquez et al., 2014) and dorsal closure (Solon et al., 2009).

Neighbor exchange events have also been suggested to play a much more general role in maintaining the balance between order and disorder in epithelia (Farhadifar et al., 2007; Marinari et al., 2012). However, under conditions of balanced growth or stasis, it is not yet known whether or not actomyosin plays a

 © 2017 Published by Elsevier Inc.
This is an open access article under the CC BY IGO license (http://creativecommons.org/licenses/by/3.0/igo/).

direct role in the process of neighbor exchange. To address this question, here we utilize the *Drosophila* pupal notum to explore the regulation and function of junction dynamics in an epithelium during a period in which it remains relatively stable in size and shape (Bosveld et al., 2012; Guirao et al., 2015). Strikingly, in this context, the impact of Myosin-dependent tension on neighbor exchange is different from that described previously. Instead of driving topological rearrangements, junctional actomyosin limits the number of neighbor exchanges in this tissue. This is explained, at least in part, by a computational model, which shows how the variance in actomyosin-based tension across cell-cell junctions over time can determine the impact of junctional actomyosin on tissue topology. Thus, as the levels of junctional Myosin II rise across the tissue over the course of development, the rate of neighbor exchange events declines, aiding the gradual refinement of tissue packing as metamorphosis reaches an end.

RESULTS

For this analysis, we began by examining apical junction dynamics in flies expressing endogenous levels of E-cadherin-GFP (Huang et al., 2009) in cells outside of the midline (Figure 1A) (Marinari et al., 2012). To facilitate the analysis of cellular dynamics, images were taken at a high frame rate (30 s intervals), between 12 and 13.5 hr after puparium formation (APF), prior to the onset of cell division, thereby avoiding the impact of cell rounding upon fluctuations in length of apical cell-cell contacts (Bosveld et al., 2012; Guirao et al., 2015) (Movie S1). We observed large numbers of neighbor exchange events throughout this period (Figures 1B–1D, 1F, and 1G and Movie S2), at a rate of 8.5 ± 1.5 × 10^{-4} T1 events per minute per junction (Figure 5J).

One of the initial goals of our analysis was to compare the junction dynamics of this system with those described for germband extension (GBE) in the fly embryo (Irvine and Wieschaus, 1994), where cell intercalation has been proposed to proceed via relatively discrete steps that occur in sequence to make the process irreversible (Bertet et al., 2004). During GBE, dorsal-ventral oriented junctions are lost, leading to the formation of four-way vertices and rosettes (Blankenship et al., 2006). New junctions are then formed and expand perpendicularly to elongate the embryo along the anterior-posterior axis (Irvine and Wieschaus, 1994). In the notum, by contrast, neighbor exchange was found to be a reversible process (Figure 1B). In many instances, quartets of cells underwent multiple rounds of neighbor exchange within the imaging period (75–180 min) (Figures 1B, 1C, S1A, and S1B). At the same time, many cells reaching a four-way vertex failed to undergo a neighbor exchange event, so that the ‘‘lost’’ junction was later reformed, restoring the original local tissue topology (not shown).

There was no apparent pattern to the timing or position of these neighbor exchange events (Figure 1D). Further, neighbor exchange in the notum was not accompanied by a significant global change in tissue area (Figure 1E) or aspect (width/length) ratio, which remained nearly constant over the 90-min imaging period (Figure 1F). This was also true at the local level. There was no correlation between the length of AJs and their orientation (Figure S1D), and junctions lost or gained during neighbor exchange did not have an orientation bias, as observed in GBE (Figure 1D). Most significantly, perhaps, while neighbor exchange in the notum involved a 90° change in the orientation of AJs, as is characteristic of T1s in other tissues, this local change in junction topology was not accompanied by a change in the shape, or area, of the associated four-cell cluster (Figures 1H–1J, S1F, and S1G). In other words, at the level of AJs, the exchange of neighbors at the center of a four-cell cluster had no effect on the overall shape of the cluster's outer edge and therefore did not induce local deformations in the tissue. Thus, neighbor exchange events in this tissue are reversible, do not lead to tissue deformation, and occur in the complete absence of cell division, delamination, and morphogenesis.

These observations led us to explore whether stochastic fluctuations in junction length, which are a ubiquitous feature of the system, might help to drive T1 transitions. To address this question, we began by asking whether the length fluctuations associated with neighbor exchange were distinct from those measured in the junction population as a whole (Figures 2A–2D and S2 and Table S1). Using persistence length (defined here as the change in length associated with periods of persistent contraction or expansion) as a measure, we found that fluctuations around neighbor exchange events are statistically indistinguishable from fluctuations in AJ length more generally (Figures 2A–2D and S2). In addition, during neighbor exchange, the dynamics of junctional loss and gain were kinetically indistinguishable and were no different from those measured during the shortening and lengthening of longer fluctuating junctions in the tissue (Figure 2E). These data suggest that AJs are subject to length fluctuations, a proportion of which fluctuate through zero to trigger neighbor exchange.

This view of the process contrasts with previous work on GBE, which has suggested that neighbor exchange involves discrete steps: shrinking junctions are first lost, generating a four-way vertex, before a new junction can be formed (Figure 2F). Under this model, during a T1 transition, one expects to observe a transient reduction in total junction length, as a junction is lost to generate a four-way vertex, followed by an increase in total junction length during the following expansion (Figure 2F, top) (Bertet et al., 2004). This was not what we observed in the notum. On average, total junction length remained constant over time (Figure 2G), so that the extent of average junctional loss/gain was offset by a corresponding increase/decrease in the length of its four first neighbors, respectively.

Roles for Non-muscle Myosin II in Driving Changes in Junction Length and Neighbor Exchange in the Notum

These data strongly suggest that the process of neighbor exchange in the notum is different to that observed during GBE, where neighbor exchange is driven by polarized pulses of actomyosin (Bertet et al., 2004; Rauzi et al., 2010), and where junction contraction is mechanistically distinct from expansion (Collinet et al., 2015). This does not, however, rule out a role for actomyosin in the process. To explore the potential role of actomyosin-based forces in driving the neighbor exchange events we observed in the notum, we labeled and imaged non-muscle Myosin II in the tissue using live imaging and fixed staining. This revealed a pool of Myosin II at apical cell-cell junctions (Figures 3A and 4A). Importantly, this localization is dependent upon the presence of AJs and was lost following RNAi-mediated

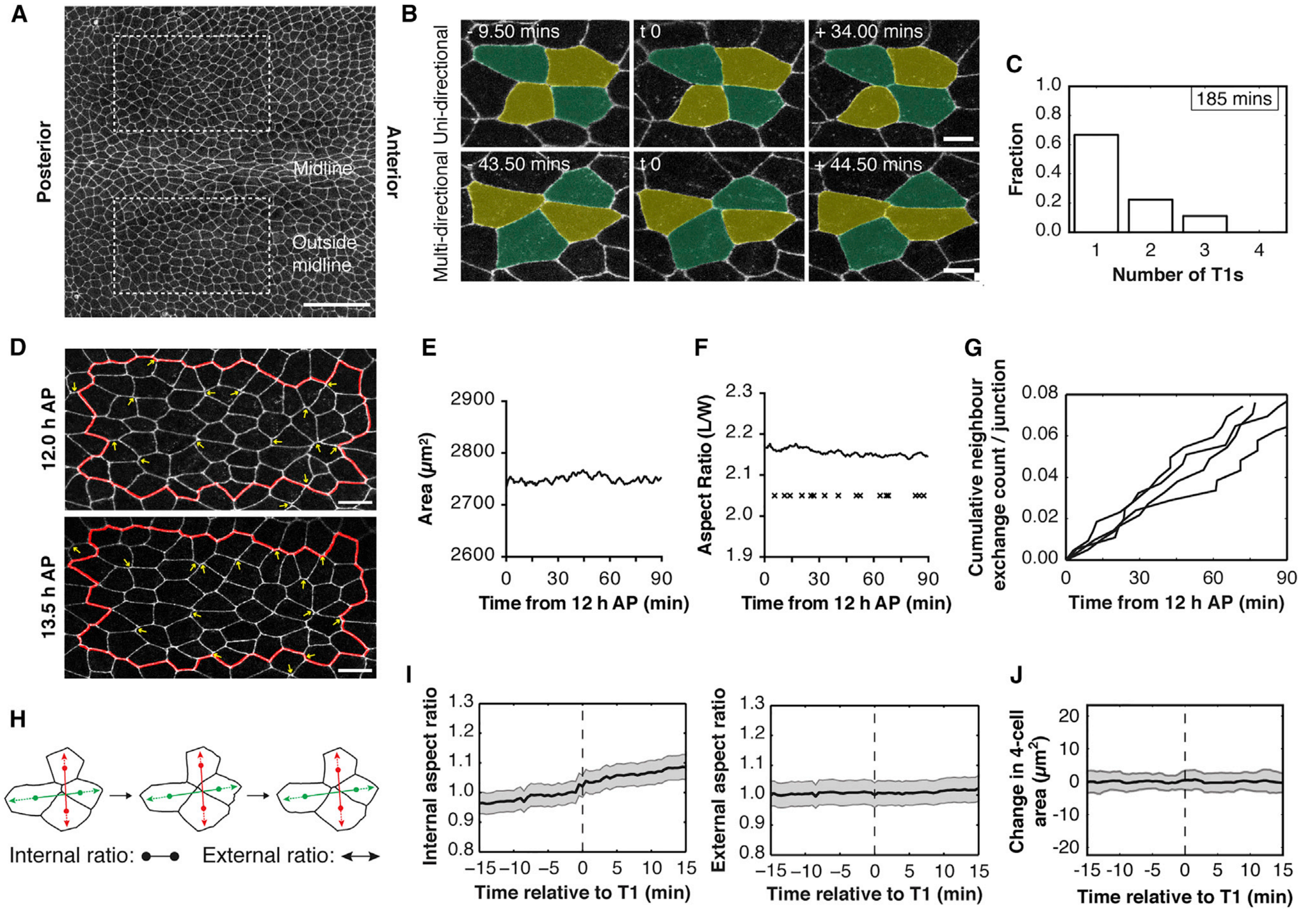

Figure 1. Neighbor Exchange Events Do Not Contribute to Tissue Morphogenesis

(A) Apical surface projection of a live *Drosophila* notum labeled with DE-cadherin-GFP at 12 hr APF. Image regions are indicated by dashed boxes. Scale bar, 50 μm.

(B) Neighbor exchange events are reversible. Top: Example of a uni-directional neighbor exchange event. Bottom: A multi-directional neighbor exchange event. Scale bar, 5 μm.

(C) Bar graph showing the fraction of multi-directional transition events for a representative fly. See Figure S1A for further n.

(D) Representative region of the notum, outside the midline, at 12.0 and 13.5 hr APF. Yellow arrows at 12 hr label junctions that are lost, and at 13.5 hr label junctions that have been gained through neighbor exchange events. Scale bar, 10 μm.

(E and F) Line plots showing the area (E) and aspect ratio (F) of the virtual clone of cells shown within the red line in (D), measured over 90 min at 30 s intervals. Crosses in (F) mark the temporal position of neighbor exchange events during this time (each cross represents the time at which a four-way vertex is formed).

(G) Cumulative frequency, for four individuals, of neighbor events over a 90-min period, normalized to the number of junctions within the frame at 12 hr APF.

(H) Diagram of a neighbor exchange event at the center of a four-cell cluster. The center of area of each cell (CoA, marked with a dot) is calculated. The internal aspect ratio is the distance between the CoAs of the cells losing a junction (red) divided by the distance between the CoAs of cells gaining a junction (green). For the external aspect ratio, the axis between the CoAs is extended out and the distance between the perimeter intersections is calculated.

(I) Mean aspect ratio (left, internal; right, external) for the four-cell cluster neighbor exchange event. Error bars represent SEM.

(J) Mean change in cell area for the four-cell cluster involved in neighbor exchange. The cell area was measured from 15 min before to 15 min after the transition and, for each four-cell cluster, the mean was subtracted from the time series. Mean area for four-cell cluster = 222.3 μm^2. Error bars represent SD n for (I) and (J), 33 exchange events from four flies.

silencing of β-catenin or DE-cadherin (Figures 3A and 3B), implying that actomyosin is physically associated with the adherens junction itself.

The overall distribution of this pool of junctional Myosin was not polarized, nor was it associated with junctions with particular orientations (Figure 4B). Similarly, Bazooka was not polarized (Figures S3A and S3B), nor was it preferentially associated with junctions that had low levels of Myosin II (Figure S3C), as has been reported for other tissues (Nakayama et al., 2008; Simoes Sde et al., 2010). In this, both Myosin II and Bazooka reinforce the view suggested above, that this region of the notum lacks a strong axis of junctional polarity. Nevertheless, it is still possible for junctionally associated actomyosin to drive changes in junctional length in this system. To address whether or not this might be case, we compared changes in junctional length (Figure 4D) with the time evolution of Myosin II-GFP intensity at cell-cell junctions, which fluctuate by ~8% around the mean (Figure 5B, middle). Strikingly, a cross-correlation analysis showed that Myosin II levels are negatively correlated with junction length. Moreover, the accumulation of non-muscle Myosin II

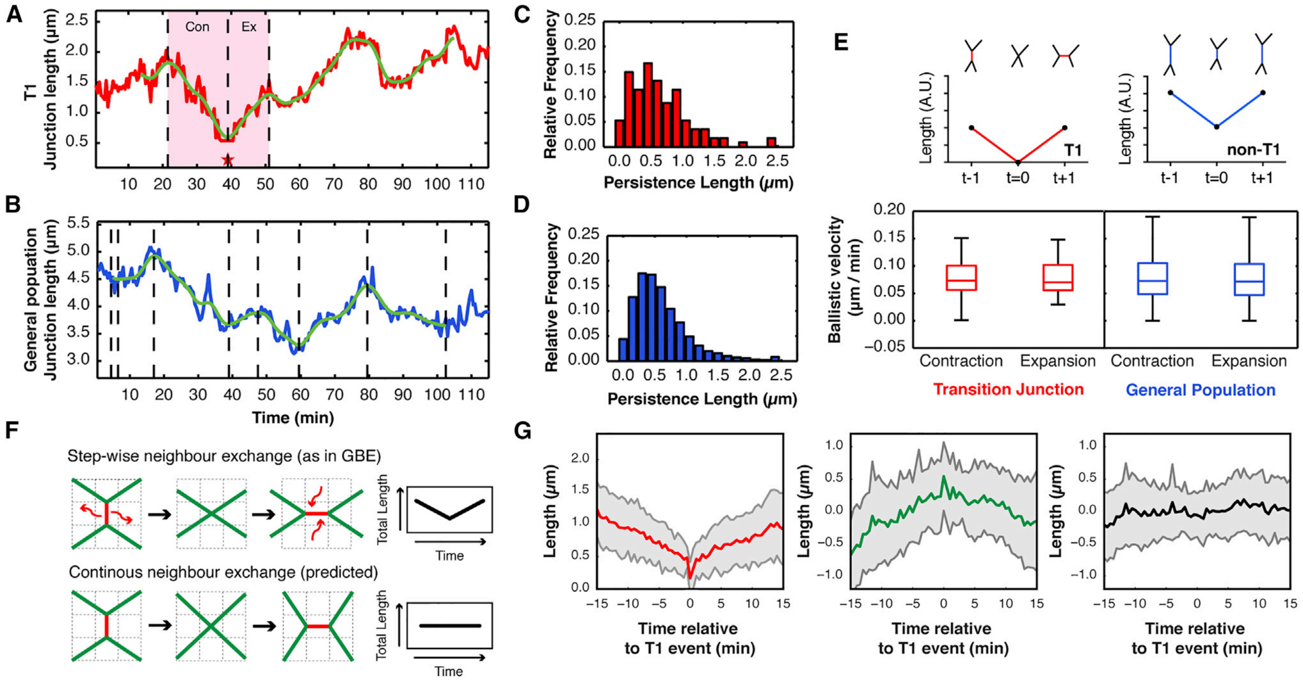

Figure 2. Neighbor Exchange Events Resemble Stochastic Fluctuations in Junction Length

(A and B) Time series line plot for (A) a junction undergoing a T1 transition and (B) a junction fluctuating in length but not undergoing a T1.

(C and D) Persistence length distributions for (C) T1 and (D) non-T1 junctions. The two distributions were compared using a two-sample Kolmogorov-Smirnov test, p value = 0.5294 (not significant).

(E) Top: Sketch of two junctions undergoing the same changes in junction length, at the same rate. While the red junction undergoes a neighbor exchange, the longer junction, in blue, does not. Bottom: Boxplots for the ballistic velocity (persistence length/persistence time) for non-T1 and T1 segments measured *in vivo*. The p values for a Kolmogorov-Smirnov test comparing the distributions are: contracting non-T1 versus expanding non-T1, p = 0.1613 (not significant); contracting T1 versus expanding T1, p = 0.9594 (not significant); all non-T1 versus all T1, p = 0.3243 (not significant). The filter setting is 20. N = 11,377 non-T1 segments and 114 T1 segments from four nota.

(F) Alternative models of neighbor exchange. Top: Stepwise neighbor exchange, as developed from germband extension. A junction is actively removed during loss and added during expansion. This causes total junction length to decrease and then increase. Bottom: Continuous neighbor exchange, whereby junctional material and total junction length is maintained throughout a transition. This requires slight movement of first neighbor vertices (middle).

(G) Average junction length changes during T1 events for wild-type tissue. Plots are color-coded according to the junction colors in (F). Left, red: Line plot of the T1 transition junction. Middle, green: First neighbors to T1 junction. Right, black: Cumulation of all five junctions involved in the transition. Gray, indicates SD. N = 27 events from four nota. For each event, the junction length time series has been aligned to the four-way vertex configuration occurring at t = 0.

at AJs preceded junction shortening by ~30–60 s (Figure 4E). In line with the data suggesting that Myosin II actively shortens junctions, Myosin II levels tended to be higher at shorter junctions, as well as at three- and four-way vertices (Figure 4C). These data suggest that the junctional pool of Myosin II generates tension that reduces the length of AJs in the notum. It should be noted that we did not observe pulsatile changes in cell areas or medial Myosin intensity, and changes in the intensity of the medial Myosin pool did not precede changes in cell area (Figures S3D–S3G). Moreover, Myosin II was present at the junctions lost as well as the junctions gained following a neighbor exchange event (Figure 4F).

Using Computational Modeling to Determine How Myosin-Dependent Junction Tension Likely Influences Neighbor Exchange

In order to better understand how stochastic fluctuations in junctional Myosin II might influence neighbor exchange events in this system, we developed a stochastic vertex model. In this model, forces acting on vertices arise from line tensions acting on cell-interfaces and from a cell area elasticity term constraining the apical cell area to a target area, as in previous studies (Farhadifar et al., 2007; Marinari et al., 2012). The model also assumes that vertices are subjected to a dynamic friction force, such that the equation of motion of the position of a vertex x_i is given by

$$\alpha \frac{dx_i}{dt} = f_i, \quad \text{(Equation 1)}$$

with α a friction coefficient, and the force f_i dependent in particular on the line tensions along cellular junctions, γ_{ij} (Figure 5A). To account for the fluctuations in junction length observed *in vivo*, we introduced stochastic fluctuations in line tensions into the model (Equation 2). Under this simple assumption, the dynamic evolution of each vertex now depends both on the fluctuating forces to which they are subjected and on an effective friction coefficient that determines how quickly they respond to external forces.

Fluctuations in force in the system were implemented so as to mirror observed changes in Myosin II levels at individual junctions (measured using Spaghetti Squash-GFP; Figure 4A). In

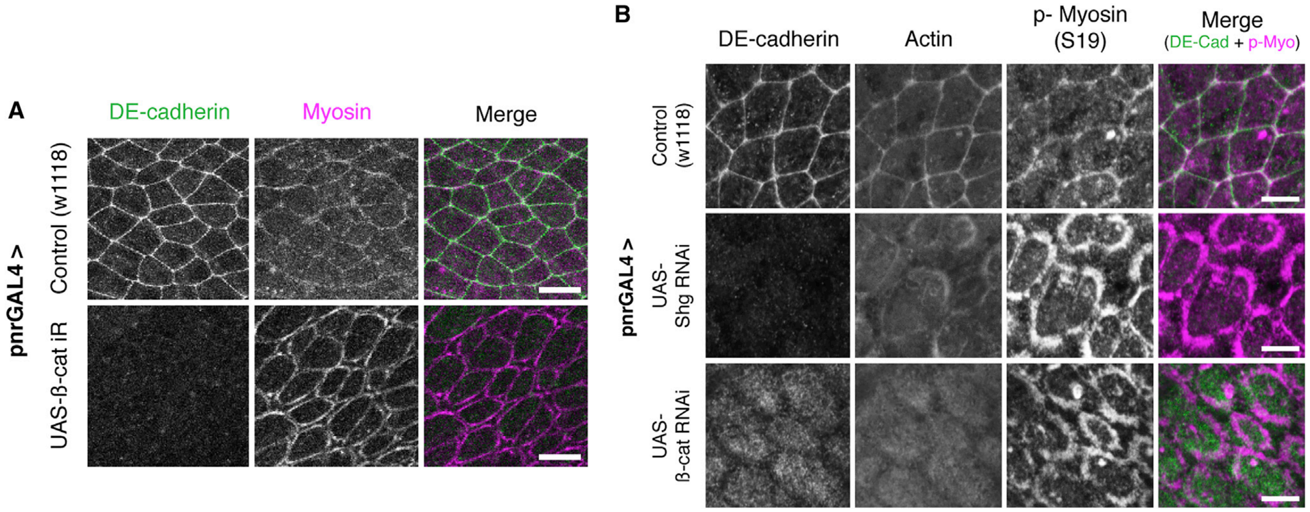

Figure 3. E-Cadherin Couples the Actomyosin Cytoskeleton to the Apical Adherens Junction

(A) Apical surface projection from a live pupa expressing ubi-E-cadherin-GFP (green) and MRLC-mCherry (magenta) for control and UAS-β-*catenin* (*armadillo*) RNAi. Scale bar, 25 μm.

(B) Representative nota of control, UAS-*Shotgun* (*DE-cadherin*) RNAi and UAS-β-*catenin* RNAi, driven by pnr-GAL4. Tissues were fixed and stained for E-cadherin (anti-GFP against DE-cadherin-GFP), F-actin (phalloidin), and phospho-Myosin II (S19). Scale bar, 5 μm.

introducing these terms, we were careful to distinguish between different sources of variation in levels of active Myosin II. As a measure of extrinsic junction-to-junction variation, we characterized the spatial variation in junctional Myosin II density across different junctions in the tissue by averaging the Myosin II intensity for each bond over the course of the 1.5–2 hr of observation. The resulting averages followed a near Gaussian distribution with a coefficient of variation $CV_e \simeq 0.13$ (Figure 5B, left), showing that average Myosin II intensities along separate cellular interfaces are different from each other, even in the absence of tissue-wide polarized distribution. To quantify the dynamic fluctuations that are intrinsic to each junction, we then quantified how the intensity of Myosin II varies at each cell-cell contact over time (Figure 5B, middle). Intrinsic Myosin II intensity fluctuations were correlated over a period of 2.2 min (Figure 5C), and the average coefficient of variation was $CV_i \simeq 0.08$; similar to the measured extrinsic variation (Figure 5B, left). We find that the variation of Myosin II intensity across junctions in the tissue at a given time, which results from both intrinsic and extrinsic fluctuations, is $CV = 0.15$ (Figure 5B, right).

With this information in hand, we formulated a vertex model in which line tensions fluctuate over time as a result of fluctuations in Myosin II, like those observed at individual cell-cell contacts (Figures 5D and S4). We implemented the following time-varying line tension γ_{ij} on the edge joining vertices *i* and *j*:

$$\frac{d\gamma_{ij}}{dt}(t) = -\frac{1}{\tau_m}\left(\gamma_{ij}(t) - \gamma_{ij}^0\right) + \xi_{ij}(t),$$

$$\left\langle \xi_{ij}(t)\xi_{kl}(t')\right\rangle = \frac{2\sigma_i^2}{\tau_m}\delta(t-t')\delta_{ik}\delta_{jl}, \qquad \text{(Equation 2)}$$

where ξ_{ij} is a white, uncorrelated noise characterizing intrinsic fluctuations, τ_m is a persistence time, and γ_{ij}^0 is a reference tension. In order to reflect extrinsic fluctuations, a line tension is obtained for every cell from a truncated normal distribution with mean γ and SD σ_e, and the tension γ_{ij}^0 is taken as the average of the line tensions of its two contributing neighbor cells. We also enforced line tensions to stay positive, and introduced a normal distribution of preferred cell areas with SD σ_A (Methods S1). We then simulated realizations of the stochastic vertex model described by Equations 1 and 2 (Figure 5E). A topological T1 transition is induced in simulations when, as a result of fluctuations in line tensions, the length of cellular interfaces falls below a defined threshold length.

Using these simulations, we first asked how cell packing is affected by initial conditions. To test this, we ran simulations starting either from a Voronoi tessellation of randomly distributed points in the plane or from a regular honeycomb packing of hexagons (Figure 5F, left). Relaxing both configurations without line tension fluctuations led to different packing states (Figure 5F, t = 0). When introducing tension fluctuations, however, the topological disorder of the tissue eventually reaches a fluctuating steady-state that is independent of the initial packing geometry (Figure 5F, right).

The rate of neighbor exchange over time was relatively constant under this model, as observed in the notum (Figure 5G, related to Figure 1G). In order to ensure that the parameters used in this fluctuating vertex model are close to those observed in experiments, we used the measured variations in Myosin II intensity in the tissue to set the ratio of extrinsic and intrinsic fluctuations in line tensions σ_i/σ_e and persistence time τ_m. We then adjusted the magnitude of fluctuations, the area elasticity and the characteristic packing time, $\tau_p = \alpha l/\gamma$, with l a characteristic cell length. For $\tau_p \simeq 4.4$ min, line tension to area elasticity ratio $\gamma/Kl^3 \simeq 0.025$, preferred area SD $\sigma_A = 0.19l^2$, and $\sigma_e \simeq 1.07\gamma$, the fluctuating vertex model accounted for the observed rate of T1 transitions and exhibited junction length and cell area fluctuations with a strength close to experimental values (S4A-P, parameters are reported in Table 1 in Methods S1). Edge length and

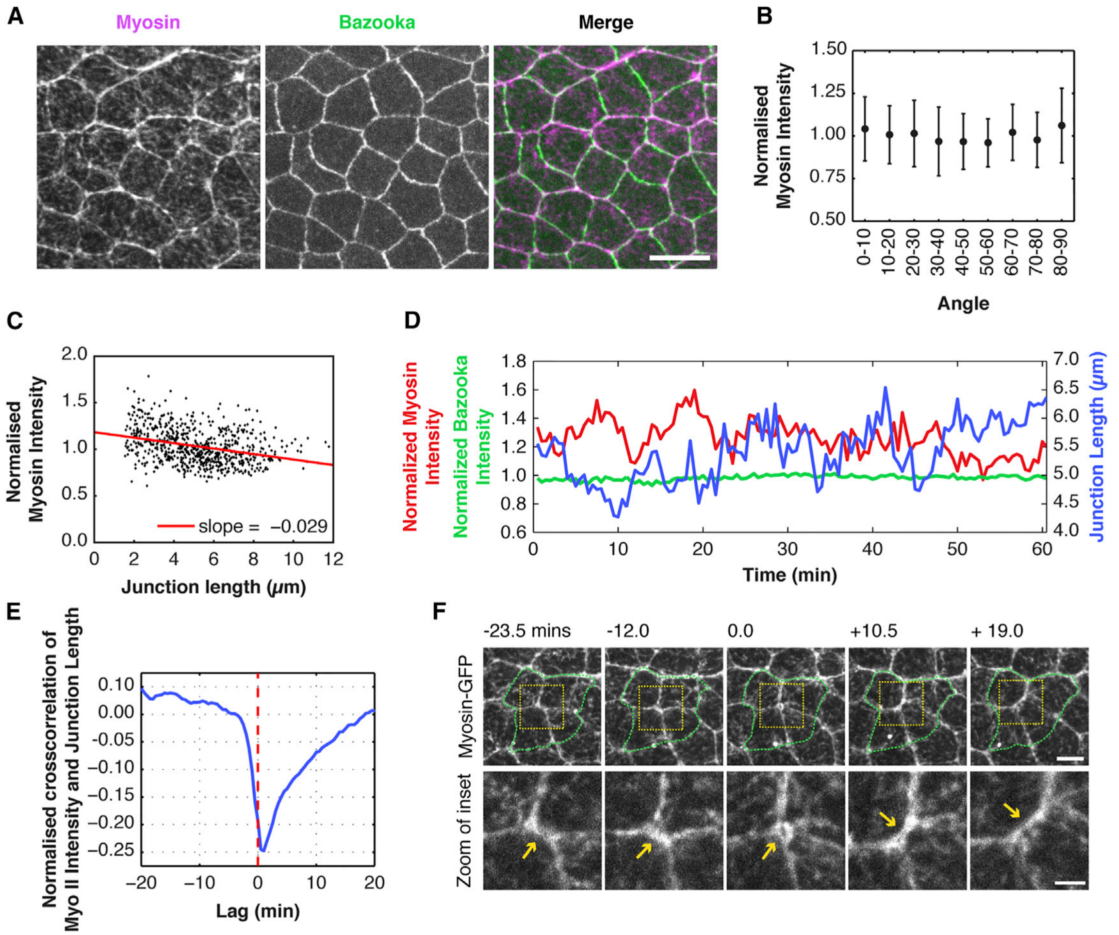

Figure 4. Myosin II Drives Changes in Junctional Length

(A) Apical surface projection of a live nota imaged with Spaghetti Squash-GFP (Myosin regulatory light chain) (magenta) and Bazooka-mCherry (green). Scale bar, 5 μm.

(B) Myosin II intensity (normalized to mean intensity in a single frame at 12 hr APF) versus angle, with respect to the AP midline (0°). Spearman's rank = −0.0155. There is no significant correlation between Myosin II intensity and angle, p = 0.6875 (not significant).

(C) Normalized Myosin-GFP intensity of individual junctions versus junction length. (slope = −0.029, R^2 = 0.10. Spearman's rank = −0.34. n for (B) and (C) = 688 junctions/3 nota).

(D) A line plot showing a representative example of junction length (blue), Myo-II-GFP intensity (red), Baz-mCh intensity (green) plotted as a function of time. Both Baz-mCh and Myo-GFP intensities are normalized to mean tissue intensity.

(E) Mean normalized cross-correlation for Myosin intensity and junction length $\overline{\Delta I(t)\Delta l(t+\Delta t)}/(\sigma^I\sigma^l)$ as a function of lag time Δt, with σ^I and σ^l the intensity and length SD. The normalized cross-correlation is calculated for each junction and then averaged over different junctions. n = 315 junctions/3 nota, imaged at 30 s intervals for 60 min. The minimum occurs at 1 min with changes in Myosin intensity preceding changes in junction length.

(F) Apical surface projection montage of a representative four-cell cluster (outlined in green, top), labeled with Sqh-GFP, undergoing a neighbor exchange event. Bottom: A zoom of the transitioning junction (yellow inset, top; highlighted by yellow arrows, bottom) showing Myo-II intensity as the junction contracts and expands. Scale bars: top, 5 μm; bottom; 2 μm.

line tension fluctuations were negatively correlated, with a lag time of ∼1 min, consistent with experiments (Figure 5H). When comparing the simulated and experimental distributions of edge lengths, we found that the distribution of junctions in the fly notum was skewed toward short junctions (Figure S4Q), while simulated tissues were more ordered than their experimental counterparts (Figure S4R). Overall, this analysis supports the idea that T1 transitions in the tissue are driven, at least in part, by the stochastic changes in the length of cell-cell contacts that result from intrinsic and extrinsic fluctuations in Myosin-dependent tension across AJs.

Having established a model based on the wild-type tissue at 12–13.5 hr APF, we wanted to determine the influence of the mean line tension on tissue dynamics. To do so, we altered the mean line tension γ, while keeping all other parameters constant. Under these conditions, in which the strength of fluctuations does not vary with Myosin II intensity, we observed a steady decrease in length fluctuations with increasing active Myosin II (Figure 5I and Movie S3). We then used simulations to determine how neighbor exchange frequencies and topological order changed with average line tension. An increasing line tension led both to a decrease in the number of T1 transitions (Figure 5J)

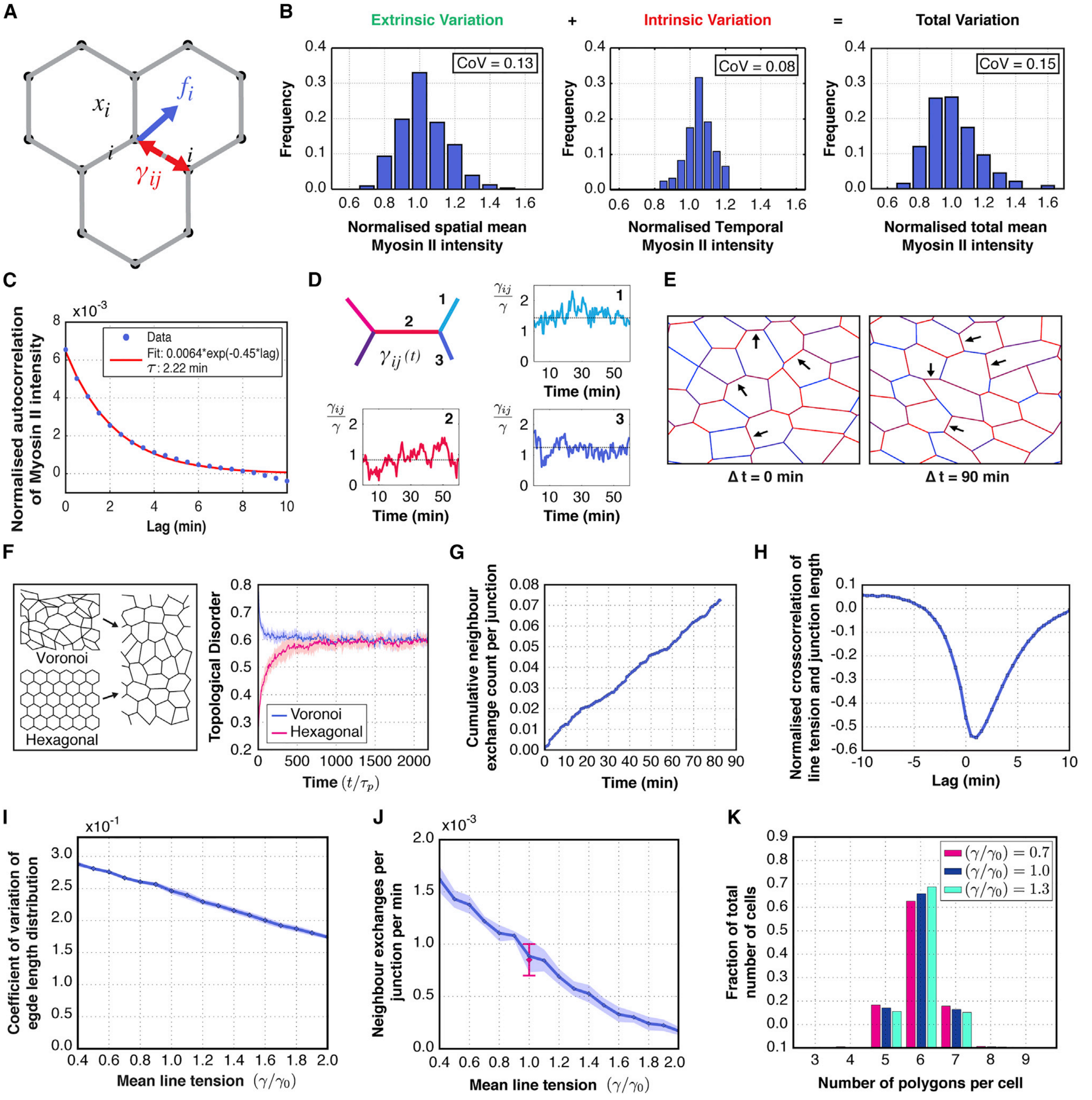

Figure 5. A 2D Vertex Model, with Fluctuating Line Tensions, Recapitulates Experimental Rates of Topological Transitions

(A) Schematic of the vertex model: each vertex i is subjected to a mechanical force f_i, that depends on line tensions across cellular interfaces γ_{ij}.

(B) Left: Histogram for the distribution of time-averaged junctional Myosin II intensities for different junctions in the notum (extrinsic variation), n = 333 junctions/3 nota, coefficient of variation [CoV] = 0.13, 60 min = 120 time points. Middle: Distribution of the Myosin II intensity over time (intrinsic variation) for a single junction with CoV = 0.08. The average CoV for Myosin II intensity over time is 0.08. n = 333 junctions/3 nota, 60 min = 120 time points. Right: Histogram showing the spatial distribution of Myosin II intensities for a single time frame at 12.5 hr APF (total variation), n = 333 junctions/3 nota, CoV = 0.15.

(C) Autocorrelation for Myosin II intensity variation on single junctions $(\overline{I(t)I(t+\Delta t)} - \bar{I}^2)/\bar{I}^2$, with $\bar{I}$ the mean intensity of the junction, as a function of lag time Δt (blue). Red curve: best fit for an exponential of the form $y = a\ exp(-b\ x)$. The coefficients are: $a = 0.0064 \pm 0.00012$ $b = -0.451 \pm 0.0130$. n = 315 junctions/3 nota.

(D) In the stochastic vertex model, line tensions fluctuate over time according to an Ornstein-Uhlenbeck process, with mean tension varying over different junctions. 1–3 are realizations of line tensions over time. The mean of each junction's fluctuations γ_{ij}^0 (dotted lines) is taken from a probability distribution parameterized by the line tension γ. Preferred cell areas are taken from a normal distribution.

(legend continued on next page)

and to a more ordered tissue, as measured by a larger fraction of cells with six sides (Figure 5K). Thus, the 2D vertex model suggests that under a model in which local Myosin II can drive a neighbor exchange event, an increase in global junctional Myosin II and line tension across the tissue will tend to drive the tissue toward an ordered, hexagonally packed state.

Consequences of Developmental Changes in Myosin II Organization

Since levels of junction tension change with developmental time in this tissue (Bosveld et al., 2012; Marinari et al., 2012), we wanted to determine if the relationship seen in the model between increasing line tension and increasing rate of topological transitions was born out during the course of notum development. To explore this question in detail, we imaged the notum over a much longer period from 20 hr APF. This starting point was chosen to exclude the period in which a global wave of cell division disorders the tissue (14–20 hr APF). Moreover, from 20 hr APF, development in the tissue is accompanied by a gradual shift in Myosin II localization, as apico-medial Myosin is lost and prominent, relatively uniform actomyosin cables are formed around apical cell-cell junctions (Figure 6A). Using the junctional recoil induced by laser ablation as a measure of tension across a junction, we were able to confirm that this visible rise in the level of junctional Myosin II is associated with a significant increase in line tension (Figure 6B and Movie S4); as suggested by previous studies (Guirao et al., 2015; Marinari et al., 2012).

To test whether this increase in tension is accompanied by changes in tissue order, we examined tissue packing in flies imaged from 20 hr through to 30 hr APF. As seen in the model, the observed increase in the level of junctional Myosin II over time was accompanied by a significant ($\sim$15%) increase in the proportion of hexagonally packed cells (Figures 6C and 6D) and by a reduction in the variance of junction lengths across the tissue (Figure 6E). Furthermore, increasing line tensions were associated with an overall reduction in the rate of neighbor exchange from 20 to 30 hr APF (Figures 6F and 6G). Although we observed a temporary rise in the neighbor exchange rate between 24 and 28 hr, when examined closely this appeared to be the result of a change in the relative growth rate of sensory organ precursor cells at this time, which was associated with an increase in the neighbor exchange rate of cells in their vicinity (Figure S5). Thus, overall, while the increase in junctional Myosin II is associated with a decrease in the rate of neighbor exchange events, the T1 transitions that do occur aid the approach to optimal cell packing.

Finally, it was important to test whether the observed increase in the level of junctional non-muscle Myosin II across the tissue is sufficient to explain observed changes in the level of neighbor exchange events that were associated with developmental progression. To do so, building upon previously published work, we perturbed the function of the Myosin activator Rho kinase (Rok) in early development (12 hr APF) (Simoes Sde et al., 2010; Verdier et al., 2006). Manipulating levels of Rok seemed a good choice for this perturbation analysis since Rok is present at AJs and at four-way vertices (Figure 7A), is required for Myosin II phosphorylation and activation (tested using Rok RNAi, Figure 7B), is sufficient to increase levels of junctional Myosin and p-Myosin when expressed as a constitutively active kinase (Rok-CAT expressed under the pnr-GAL4 driver, Figure 7B), and can increase the tension across AJs in the tissue (Figure 7C). Having established this, we were in a position to use Rok-CAT to artificially elevate junctional Myosin II in early development. In line with the model, the increase in junctional Myosin induced by the overexpression of Rok-CAT led to a decrease in the frequency of neighbor exchange events, mirroring the late notum. It also reduced the numbers of reversible T1 events (Figure 7E). Conversely, Rok RNAi led to a corresponding increase in neighbor exchange events (Figure 7D) and to an increase in the chances of a T1 being reversed (Figure 7E). Since the small pool of medial Myosin is also lost following Rok RNAi (Figure 7B), one can rule out an essential role for medial Myosin II in driving neighbor exchanges. Moreover, treatments that used perturbations in Moesin function to change the relevant levels of junctional and medial Myosin suggest that it is the junctional pool of Myosin that functions to damp neighbor exchange (Figure S6). Importantly, these results were confirmed using phospho-null (sqh-AA) and phospho-active (sqh-EE) forms of Myosin regulatory light chain to decrease or increase Myosin II activity, respectively (Figures S6A and S6G).

Previously, we proposed a role for neighbor exchange in midline cell delamination, an event that begins at $\sim$14 hr APF.

(E) Representative example of one simulation at two time points. The edge color, for each interface, corresponds to the magnitude of line tension (blue = low, red = high). Neighbor exchange events occur when a junction length falls below a threshold. Black arrows at $\Delta t = 0$ min label example junctions that are lost at later time points, and at $\Delta t = 90$ min label junctions that have been gained through neighbor exchange.

(F) Topological disorder as a function of time for two different initial packing configurations, regular honeycomb packing and packing obtained by relaxation without noise of a Voronoi tessellation, with identical parameters. Topological disorder is defined as the SD of the number of edges per cell within the tissue at one time point. The convergence of simulations to the same average disorder indicates that the steady state of the fluctuating vertex model is independent of the initial packing geometries. For (F), (I), and (J), the shaded area represents the SD over ten simulations.

(G) Cumulative count of neighbor exchange events for a representative simulation, with wild-type parameter fitting, over a period of 80 min, normalized to the number of junctions in the frame at t = 0 min.

(H) Cross-correlation of simulated line tensions and junction lengths $\overline{\Delta\gamma(t)\Delta l(t+\Delta t)}/\sigma_\gamma\sigma_l$ as a function of lag time Δt, with σ_γ and σ_l the intrinsic line tension and length SD (compare with Figure 4E). The normalized cross-correlation is calculated for each junction and then averaged over different junctions. A change in line tension precedes changes in junction length by approximately 1 min.

(I) Coefficient of variation of junction length distribution over space CV_L (Methods S1), for tissues simulated with varying average line tension. The model suggests that the variation of junction lengths over different junctions decreases with increasing mean line tension (γ). γ/γ_0=1.0 represents the line tension set for wild-type parameter fitting. Pink: wild-type rate of neighbour exchange with SD, as measured between 12 and 13.5 hr APF.

(J) Simulated neighbor exchange rate as a function of mean line tension. Simulations reproduce the measured rate of T1 transitions for the wild-type tissue (γ/γ_0 = 1.0). The rate of neighbor exchange events is dependent on mean line tension. Higher line tensions lead to a decrease in the T1 transition rate.

(K) Fraction of cells according to the number of neighbors. An increase in mean line tension (γ/γ_0) (above 1.0), keeping other parameters constant, leads to an increase in the proportion of hexagonal cells.

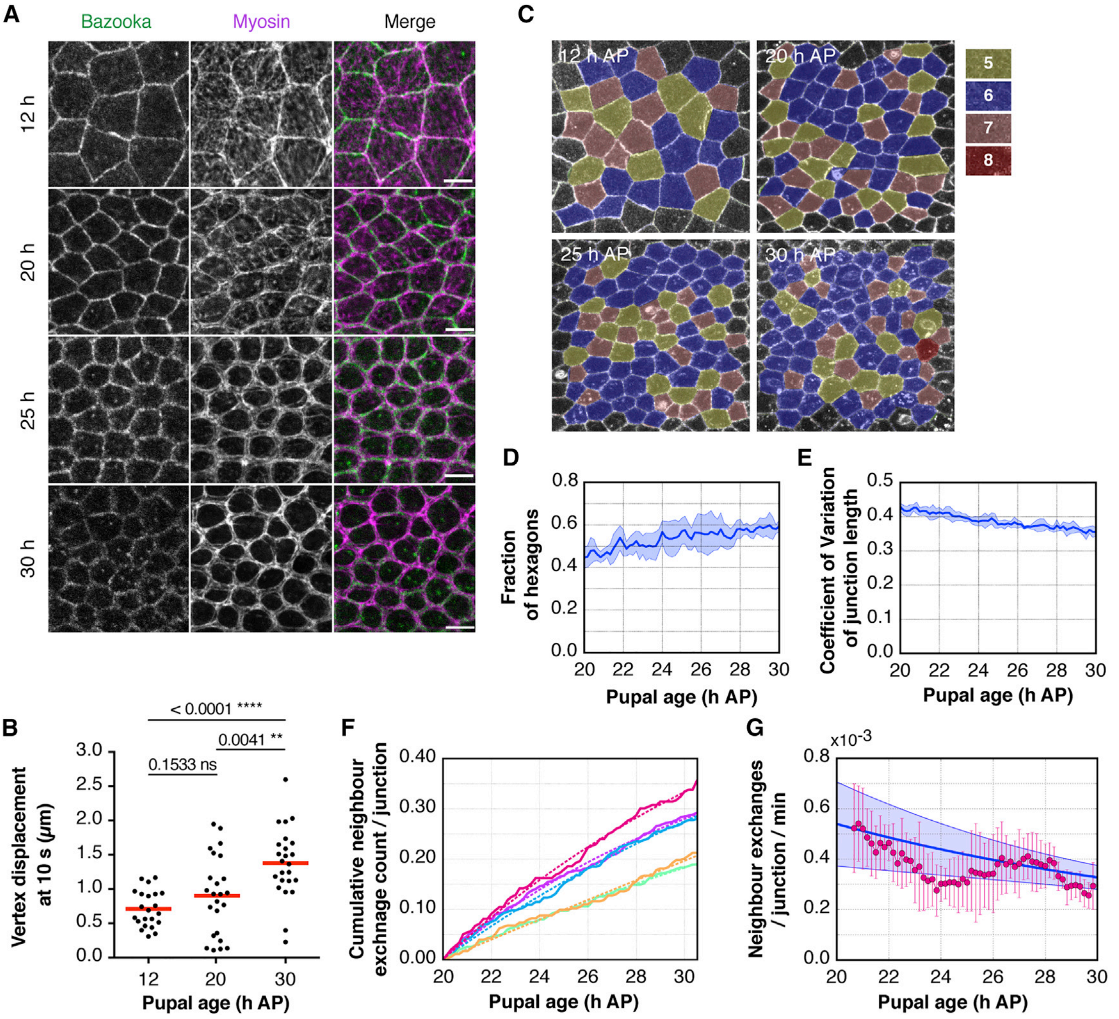

Figure 6. An Increase in Junctional Myosin II over Developmental Time Improves Tissue Packing

(A) Apical surface projection from a live pupa expressing MRLC-GFP (magenta) and Baz-mCh (green). Scale bar, 10 μm.

(B) Scatter boxplot quantification of the total vertex displacement at 10 s of single junctions after laser dissection at 12, 20, and 30 hr APF. Line indicates the median. n = 22–24 cuts from 5 to 7 flies.

(C) Representative regions of nota at increasing pupal development ages. Cells are colored by number of sides.

(D) Line graph showing the average fraction (with SD) of hexagons within regions of nota, over time from 20 hr APF. n = 86–176 cells/5 nota.

(E) Average (and SD) junction length coefficient of variation over time from 20 hr APF, calculated across junctions in the tissue. n = 296–576 junctions/5 nota.

(F) Individual cumulative neighbor exchange events for each tissue, normalized to the number of junctions at 20 hr APF. Dotted lines indicate a fit of the exponential ($a(1-\exp(-b \times t))$) to each individual curve.

(G) Neighbor exchange rates from 20 to 30 hr APF, inferred from (F). Pink, T1 transition rates, with SD, for 90 min intervals calculated from the form $T1(t) = (C(t+\Delta t/2) - C(t-\Delta t/2))/\Delta t$. Blue, mean of the derivatives (with SD) of the exponential fits from (F).

This led us to explore how perturbations in the levels of junctional Myosin affect delamination in the tissue. In line with the data above, increased Myosin levels, induced by Rok-CAT expression, were found to block cell delamination in the midline. Conversely, Rok RNAi increased rates of midline delamination and caused cells to delaminate outside of the midline, something never observed in the wild-type (Figure S7). These observations confirm a role for junctional Myosin in the inhibition of neighbor exchange and suggest an important role for neighbor exchange in crowding-induced epithelial cell delamination (Marinari et al., 2012).

In sum, these data suggest that increasing the average levels of Myosin does not promote neighbor exchange in the notum, as might have been expected based upon work observed in other tissues and systems. Instead, in this relatively isotropic tissue, increasing levels of junctionally associated Myosin II actively limits neighbor exchange.

DISCUSSION

In the context of a planar polarized tissue, the localization of Myosin II along cell-cell contacts, with a specific orientation,

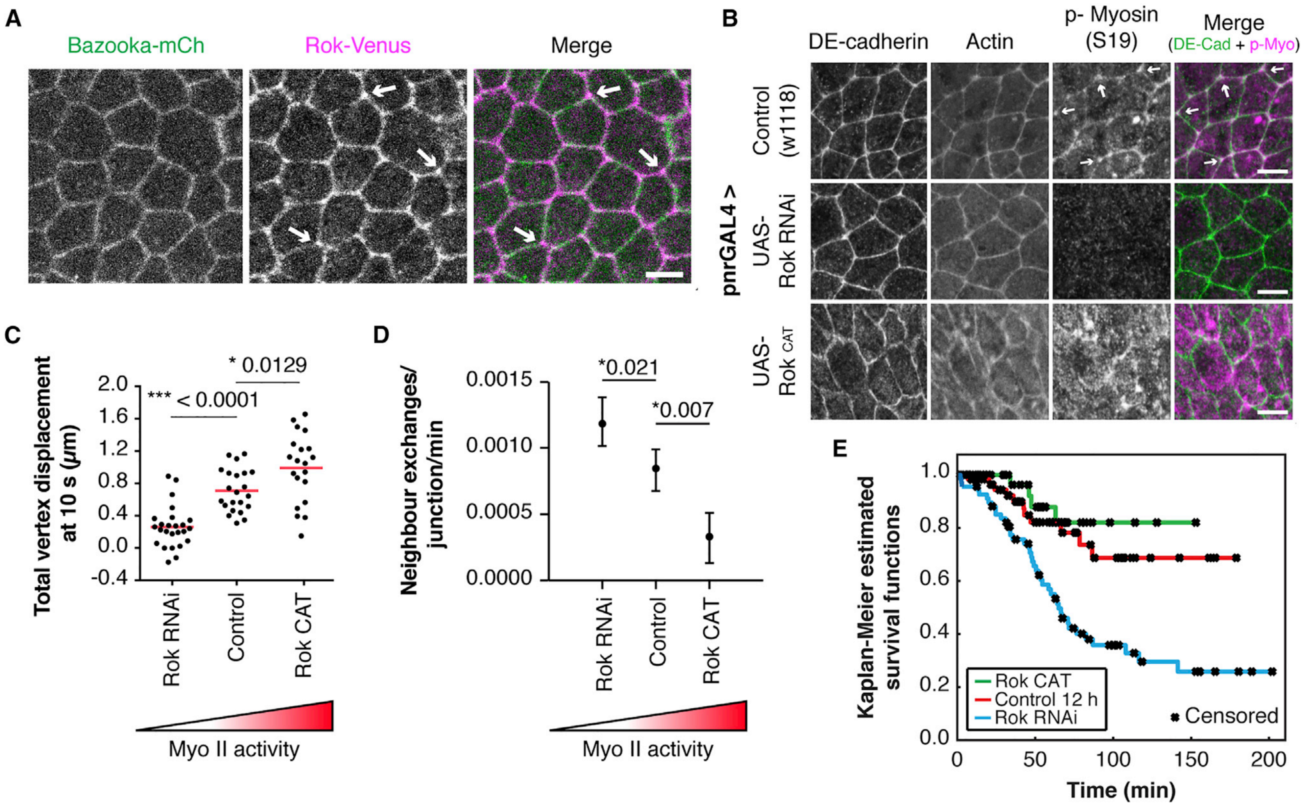

Figure 7. Changes in Myosin II Activity Tune Neighbor Exchange Rates and Reversibility

(A) Apical surface projection of a live nota expressing Venus:Rok (K116A) (magenta) and Baz-mCh (green). Scale bar, 5 μm. Arrows indicate enrichment of Rok at three-way vertices.

(B) Changing levels of Myo-II with changing Rok activity are confirmed through immunohistochemistry. Scale bar, 5 μm. Arrows, in control, indicate p-Myosin II enrichment at three-way vertices.

(C) Quantification of total vertex displacement at 10 s after laser dissection of single junctions in 12–13.5 hr APF pupae expressing Rok RNAi and Rok-CAT. Dots indicate individual experiments, line represents median. 4–7 flies/condition. p values calculated from unpaired t tests.

(D) Quantification of normalized T1 transition rates for altered levels of Rok and subsequent Myo-II activity. Dot indicates mean, tails show the data range. n = 3–5 flies/condition, 184–360 (mean of 227) junctions per fly and 98–236 (mean of 157) min/experiment. p values calculated from Kolmogorov-Smirnov tests.

(E) Kaplan-Meier survival curves showing the probability that a neighbor exchange event is uni-directional for a given length of time. From the survival curves, the probability of a configuration persisting for at least 150 min, along with the 95% confidence interval, is: control 0.687 (0.5175, 0.8567), UAS-Rok RNAi 0.258 (0.1250, 0.3915), UAS-Rok-CAT, 0.821 (0.6572 0.9841). A log rank test is used to determine if differences between the survival curves are statistically significant. Control versus UAS-Rok RNAi, *p = 0.00041; control versus UAS-Rok-CAT, p = 0.27399 (not significant).

can generate contractile forces that contribute to tissue remodeling on a macroscopic scale, as individual cell-cell contacts are lost and new contacts form. This has been studied in detail by many groups in the context of the developing embryo. How though does Myosin-dependent tension contribute to neighbor exchange in epithelia at steady state—like the adult gut or skin?

In this paper, we explore this question by investigating the role for Myosin II in a stable epithelium, the fly pupal notum, during a period of developmental time in which there are no cell divisions, no cell delamination, and no overt changes in tissue shape or size. Strikingly, in this tissue, Myosin II limits fluctuations in AJ length and, as a consequence, neighbor exchange. Therefore, with developmental time, as levels of junctional Myosin II increase in a relatively isotropic manner, there is a corresponding decrease in the rates of neighbor exchange and an increase in tissue order, as seen by a decrease in junction length variance and by an increased proportion of hexagonal cells, both measures of improved cell packing. In this way, changes in Myosin II activity levels and localization contribute to the refinement of the tissue observed at the end of development.

Although these observations might appear to conflict with studies of neighbor exchange in other tissues where Myosin II has been shown to drive neighbor exchange, the function of the molecules involved seems to be identical. Thus, in the notum, DE-cadherin and β-catenin couple Myosin II to cell-cell interfaces enabling Myosin to influence tissue packing. In addition, increases in the level of Myosin II at junctions are associated with increased junction tension. As a result of this, junctions with high levels of Myosin II tend to be shorter than those with low Myosin II levels. Moreover, a cross-correlation analysis shows that changes in Myosin II levels precede, and therefore likely drive, changes in junction length, as one would

expect if the recruitment of Myosin II to a junction led to its contraction.

Why then is the impact of Myosin action at the level of the tissue so different in different systems? Our model suggests that the answer may lie in the spatial organization and temporal control of Myosin II. The ability of junctional Myosin II to drive the loss of a specific junction will depend on the tension acting at neighboring cell-cell junctions that resist its contraction. Thus, neighbor exchange will be favored in tissues where there is a high variance in Myosin II levels between neighboring junctions (see Figure 7A), as exemplified by the early fly embryo, where planar polarization in the developing germband generates extreme differences in the levels of Myosin II at perpendicular junctions (Pare et al., 2014; Simoes Sde et al., 2010), driving efficient and directed neighbor exchange. Conversely, in a tissue like the notum, where the distribution of Myosin II is relatively isotropic but fluctuates in time and space (see Figure 7C), the impact of Myosin on the rate of topological transitions will depend on the balance between the average force generated on cell edges and the spatial and temporal fluctuations in these forces. Thus, the impact of an increase in the average levels of Myosin across a tissue on tissue order will depend on the change in variance. If, as observed in the notum, the increase in junctional Myosin and line tension is accompanied by a visible decrease in the spatial variation of Myosin II (Figure 6A), neighbor exchange rates will slow and the tissue will tend to become more ordered with time. Our analysis therefore suggests that epithelia can finely tune their behavior by controlling the average levels of junctional Myosin II and the temporal and spatial variation in its localization at individual junctions.

We note here that while the impact of Myosin II on junctional length fluctuations can explain the observed changes in tissue organization, Myosin II is also likely to play additional roles in the process of neighbor exchange in the tissue. This is suggested by the observed accumulations of Myosin II and Rok at tri-cellular junctions (Figures 7A and 7B). Thus, in limiting neighbor exchange, Myosin II may also limit the ability of fluctuations in junction length to induce smooth passage through a four-way vertex. This may help explain the reduction in junctional reversals seen with increasing levels of Myosin II (Figure 7E), and may explain why a subset of junctions pause at four-way vertices (not shown).

Finally, this study shows how local fluctuations in the activity, localization, and levels of a molecule, in this case Myosin II, can drive local changes in cell shape, to produce larger changes in tissue order. In this way, our analysis of length fluctuations bridges the molecular, cellular, and tissue scales. While this type of analysis remains in its infancy, it is likely to be important for coming to a mechanistic understanding of a wide range of biological processes. Moreover, our analysis shows how the emergence of tissue order can be driven by apparently stochastic fluctuations (Cohen et al., 2011) that are the inevitable consequence of the action of small numbers of molecules, rather than by a directed developmental program. While it may not be possible to use stochastic processes to aid rapid morphogenetic events, like those that accompany early embryonic development, organizing a tissue in this way has its advantages. The use of this type of system can help to ensure that an epithelium is robust to both intrinsic (e.g., cell division and delamination) and extrinsic (e.g., forced deformation) perturbations. Thus, fluctuation-induced changes in cell packing, of the type we see here, would seem to be a good way of maintaining integrity in a dynamic, living epithelium. We therefore expect to see similar processes throughout the animal world.

STAR★METHODS

Detailed methods are provided in the online version of this paper and include the following:

- KEY RESOURCES TABLE
- CONTACT FOR REAGENT AND RESOURCE SHARING
- EXPERIMENTAL MODEL AND SUBJECT DETAILS
  - *Drosophila* Genetics
  - Experimental Genotypes
- METHOD DETAILS
  - Notum Dissection for Live Imaging
  - Microscopes Used for Live Imaging
  - Laser Ablation
  - Notum Dissection for Immunohistochemistry
  - Theoretical and Computational Models
- QUANTIFICATION AND STATISTICAL ANALYSIS
  - Image Processing and Statistics
  - Laser Ablation Measurements
  - Aspect Ratio Quantification
  - Image Segmentation
  - Analyzing Junction Fluctuation Behavior
  - Detecting Neighbor Exchange Events
  - Fluorescence Intensity Measurements

SUPPLEMENTAL INFORMATION

Supplemental Information includes seven figures, one table, supplemental text, and four movies and can be found with this article online at https://doi.org/10.1016/j.devcel.2017.09.018.

AUTHOR CONTRIBUTIONS

S.C., C.S., and B.B. initially conceived the project. S.C., G.S., and B.B. wrote the manuscript. S.C. designed, performed, and analyzed fly experiments under the guidance of B.B. C.S. designed the data analysis pipeline and wrote the analysis software package, under the guidance of A.K. and B.B., performed data analysis (along with S.C.), and helped J.B. with simulation analysis. J.B. and M.d.G. developed, designed, and ran simulations of the 2D vertex model, under the guidance of G.S. S.C., C.S., J.B., M.d.G., G.S., and B.B. jointly developed the ideas proposed in this manuscript.

ACKNOWLEDGMENTS

We are grateful to the Bloomington *Drosophila* Stock Center (NIH P40OD018537), the VDRC Stock Center, the National Institute of Genetics Fly Stock Center, Yohanns Bellaiche, Jennifer Zallen, and Eric Wieschaus for fly stocks used in this study. We thank Paromita Majumber and Jonathan Gale (UCL) for help with the laser ablation experiments, Andrea Dimitracopoulos (MRC-LMCB, UCL) for laser ablation analysis assistance, Yanlan Mao (MRC-LMCB, UCL) for imaging assistance, Natalia Bulgakova (University of Sheffield) for GBE imaging assistance, the Oliferenko lab (KCL/Crick) for spinning disk access and assistance, and Tom Wyatt (LCN, UCL), Julia Duque (CBMSO), and Guillaume Charras (LCN, UCL) for cell culture contributions. This work was supported by the Wellcome Trust (092840/Z/10/Z to S.C.);

EPSRC - CoMPLEX PhD program UCL (C.S.); Cancer Research UK (C1529/A9786; C1529/A17343 to B.B.); the MRC-LMCB, University College London (MC_U12266B to S.C., C.S., and B.B.); the Francis Crick Institute, which receives its core funding from Cancer Research UK (FC001317), the UK Medical Research Council (FC001317), and the Wellcome Trust (FC001317) (G.S., J.B., and M.d.G); and ERASMUS+ (J.B.).

Received: September 13, 2016
Revised: July 24, 2017
Accepted: September 22, 2017
Published: October 26, 2017

REFERENCES

Aigouy, B., Farhadifar, R., Staple, D.B., Sagner, A., Roper, J.C., Julicher, F., and Eaton, S. (2010). Cell flow reorients the axis of planar polarity in the wing epithelium of *Drosophila*. Cell *142*, 773–786.

Bertet, C., Sulak, L., and Lecuit, T. (2004). Myosin-dependent junction remodelling controls planar cell intercalation and axis elongation. Nature *429*, 667–671.

Blankenship, J.T., Backovic, S.T., Sanny, J.S., Weitz, O., and Zallen, J.A. (2006). Multicellular rosette formation links planar cell polarity to tissue morphogenesis. Dev. Cell *11*, 459–470.

Bosveld, F., Bonnet, I., Guirao, B., Tlili, S., Wang, Z., Petitalot, A., Marchand, R., Bardet, P.L., Marcq, P., Graner, F., et al. (2012). Mechanical control of morphogenesis by Fat/Dachsous/Four-jointed planar cell polarity pathway. Science *336*, 724–727.

Brand, A.H., and Perrimon, N. (1993). Targeted gene expression as a means of altering cell fates and generating dominant phenotypes. Development *118*, 401–415.

Busson, D., and Pret, A.M. (2007). GAL4/UAS targeted gene expression for studying *Drosophila* Hedgehog signaling. Methods Mol. Biol. *397*, 161–201.

Classen, A.K., Anderson, K.I., Marois, E., and Eaton, S. (2005). Hexagonal packing of *Drosophila* wing epithelial cells by the planar cell polarity pathway. Dev. Cell *9*, 805–817.

Cohen, M., Baum, B., and Miodownik, M. (2011). The importance of structured noise in the generation of self-organizing tissue patterns through contact-mediated cell-cell signalling. J. R. Soc. Interface *8*, 787–798.

Collinet, C., Rauzi, M., Lenne, P., and Lecuit, T. (2015). Local and tissue-scale forces drive oriented junction growth during tissue extension. Nat. Cell Biol. *17*, 1247–1258.

Crocker, J., Crocker, J., and Grier, D. (1996). Methods of digital video microscopy for colloidal studies. J. Colloid Interface Sci. *179*, 298–310.

Desai, R., Sarpal, R., Ishiyama, N., Pellikka, M., Ikura, M., and Tepass, U. (2013). Monomeric alpha-catenin links cadherin to the actin cytoskeleton. Nat. Cell Biol. *15*, 261–273.

Farhadifar, R., Roper, J.C., Aigouy, B., Eaton, S., and Julicher, F. (2007). The influence of cell mechanics, cell-cell interactions, and proliferation on epithelial packing. Curr. Biol. *17*, 2095–2104.

Fristrom, D. (1988). The cellular basis of epithelial morphogenesis. A review. Tissue Cell *20*, 645–690.

Georgiou, M., Marinari, E., Burden, J., and Baum, B. (2008). Cdc42, Par6, and aPKC regulate Arp2/3-mediated endocytosis to control local adherens junction stability. Curr. Biol. *18*, 1631–1638.

Gibson, M.C., Patel, A.B., Nagpal, R., and Perrimon, N. (2006). The emergence of geometric order in proliferating metazoan epithelia. Nature *442*, 1038–1041.

Guillot, C., and Lecuit, T. (2013). Mechanics of epithelial tissue homeostasis and morphogenesis. Science *340*, 1185–1189.

Guirao, B., Rigaud, S.U., Bosveld, F., Bailles, A., Lopez-Gay, J., Ishihara, S., Sugimura, K., Graner, F., and Bellaïche, Y. (2015). Unified quantitative characterization of epithelial tissue development. Elife *4*.

Harris, T.J., and Tepass, U. (2010). Adherens junctions: from molecules to morphogenesis. Nat. Rev. Mol. Cell Biol. *11*, 502–514.

Heisenberg, C.P., and Bellaiche, Y. (2013). Forces in tissue morphogenesis and patterning. Cell *153*, 948–962.

Huang, J., Zhou, W., Dong, W., Watson, A.M., and Hong, Y. (2009). From the Cover: directed, efficient, and versatile modifications of the *Drosophila* genome by genomic engineering. Proc. Natl. Acad. Sci. USA *106*, 8284–8289.

Irvine, K.D., and Wieschaus, E. (1994). Cell intercalation during *Drosophila* germband extension and its regulation by pair-rule segmentation genes. Development *120*, 827–841.

Lecuit, T., and Lenne, P.-F.F. (2007). Cell surface mechanics and the control of cell shape, tissue patterns and morphogenesis. Nat. Rev. Mol. Cell Biol. *8*, 633–644.

Mao, Y., Tournier, A.L., Hoppe, A., Kester, L., Thompson, B.J., and Tapon, N. (2013). Differential proliferation rates generate patterns of mechanical tension that orient tissue growth. EMBO J. *32*, 2790–2803.

Marinari, E., Mehonic, A., Curran, S., Gale, J., Duke, T., and Baum, B. (2012). Live-cell delamination counterbalances epithelial growth to limit tissue overcrowding. Nature *484*, 542–545.

Martin, A.C., Kaschube, M., and Wieschaus, E.F. (2009). Pulsed contractions of an actin-myosin network drive apical constriction. Nature *457*, 495–499.

Mason, F.M., Tworoger, M., and Martin, A.C. (2013). Apical domain polarization localizes actin-myosin activity to drive ratchet-like apical constriction. Nat. Cell Biol. *15*, 926–936.

Nakayama, M., Goto, T.M., Sugimoto, M., Nishimura, T., Shinagawa, T., Ohno, S., Amano, M., and Kaibuchi, K. (2008). Rho-kinase phosphorylates PAR-3 and disrupts PAR complex formation. Dev. Cell *14*, 205–215.

Nishimura, T., and Takeichi, M. (2009). Remodeling of the adherens junctions during morphogenesis. Curr. Top. Dev. Biol. *89*, 33–54.

Oda, H., and Tsukita, S. (2001). Real-time imaging of cell-cell adherens junctions reveals that *Drosophila* mesoderm invagination begins with two phases of apical constriction of cells. J. Cell Sci. *114*, 493–501.

Pare, A.C., Vichas, A., Fincher, C.T., Mirman, Z., Farrell, D.L., Mainieri, A., and Zallen, J.A. (2014). A positional Toll receptor code directs convergent extension in *Drosophila*. Nature *515*, 523–527.

Rauzi, M., Lenne, P.F., and Lecuit, T. (2010). Planar polarized actomyosin contractile flows control epithelial junction remodelling. Nature *468*, 1110–1114.

van Roy, F., and Berx, G. (2008). The cell-cell adhesion molecule E-cadherin. Cell. Mol. Life Sci. *65*, 3756–3788.

Royou, A., Field, C., Sisson, J.C., Sullivan, W., and Karess, R. (2004). Reassessing the role and dynamics of nonmuscle myosin II during furrow formation in early *Drosophila* embryos. Mol. Biol. Cell *15*, 838–850.

Schindelin, J., Arganda-Carreras, I., Frise, E., Kaynig, V., Longair, M., Pietzsch, T., Preibisch, S., Rueden, C., Saalfeld, S., Schmid, B., et al. (2012). Fiji: an open-source platform for biological-image analysis. Nat. Methods *9*, 676–682.

Simoes Sde, M., Blankenship, J.T., Weitz, O., Farrell, D.L., Tamada, M., Fernandez-Gonzalez, R., and Zallen, J.A. (2010). Rho-kinase directs Bazooka/Par-3 planar polarity during *Drosophila* axis elongation. Dev. Cell *19*, 377–388.

Simoes Sde, M., Mainieri, A., and Zallen, J.A. (2014). Rho GTPase and Shroom direct planar polarized actomyosin contractility during convergent extension. J. Cell Biol. *204*, 575–589.

Solon, J., Kaya-Çopur, A., Colombelli, J., Brunner, D., Kaya-Copur, A., Colombelli, J., and Brunner, D. (2009). Pulsed forces timed by a ratchet-like mechanism drive directed tissue movement during dorsal closure. Cell *137*, 1331–1342.

Sun, D., Roth, S., and Black, M.J. (2010). Secrets of Optical Flow Estimation and Their Principles. In Proceedings of the IEEE Computer Society Conference on Computer Vision and Pattern Recognition (IEEE), pp. 2432–2439.

Takeichi, M. (2014). Dynamic contacts: rearranging adherens junctions to drive epithelial remodelling. Nat. Rev. Mol. Cell Biol. *15*, 397–410.

Tamada, M., Farrell, D.L., and Zallen, J.A. (2012). Abl regulates planar polarized junctional dynamics through beta-catenin tyrosine phosphorylation. Dev. Cell *22*, 309–319.

Tepass, U., Tanentzapf, G., Ward, R., and Fehon, R. (2001). Epithelial cell polarity and cell junctions in *Drosophila*. Annu. Rev. Genet. *35*, 747–784.

Vasquez, C.G., Tworoger, M., and Martin, A.C. (2014). Dynamic myosin phosphorylation regulates contractile pulses and tissue integrity during epithelial morphogenesis. J. Cell Biol. *206*, 435–450.

Verdier, V., Guang Chao, C., and Settleman, J. (2006). Rho-kinase regulates tissue morphogenesis via non-muscle myosin and LIM-kinase during *Drosophila* development. BMC Dev. Biol. *6*, 38.

Zallen, J.A., and Blankenship, J.T. (2008). Multicellular dynamics during epithelial elongation. Semin. Cell Dev. Biol. *19*, 263–270.

Zallen, J.A., and Wieschaus, E. (2004). Patterned gene expression directs bipolar planar polarity in *Drosophila*. Dev. Cell *6*, 343–355.

Zitserman, D., and Roegiers, F. (2011). Live-cell imaging of sensory organ precursor cells in intact *Drosophila* pupae. J. Vis. Exp. https://doi.org/10.3791/2706.

STAR★METHODS

KEY RESOURCES TABLE

REAGENT or RESOURCE	SOURCE	IDENTIFIER
Antibodies		
Rabbit Phospho-Myosin Light Chain 2 (Ser19)	Cell Signalling Technology	Cat: 3671; RRID: AB_10859887.
Chicken α- GFP	Abcam	Cat: ab13970; RRID: AB_300798
Goat α-Chicken, Alexa Fluor 488	Molecular Probes – ThermoFisher Scientific	Cat: a11039; RRID: AB_142924.
Goat α-Rabbit, Alexa Fluor 647	Molecular Probes – ThermoFisher Scientific	Cat: a21245; RRID: AB_2535813.
Chemicals, Peptides, and Recombinant Proteins		
Phalloidin-Tetramethylrhodamine B isothiocyanate (TRITC)	Sigma-Aldrich	Cat: P1951; RRID: AB_2315148.
DAPI (4' 6-Diamidino-2-Phenylindole, Dihydrochloride)	Molecular Probes – ThermoFisher Scientific	Cat: D1306; RRID: AB_2629482.
Experimental Models: Organisms/Strains		
D. melanogaster: w^{1118}	Bloomington Drosophila Stock Center	BDSC: 3605
D. melanogaster: sqh::mCherry	(Martin et al., 2009)	N/A
D. melanogaster: sqh^{Ax3}; sqhGFP42	(Royou et al., 2004)	N/A
D. melanogaster: DE-cadherin-GFP	(Huang et al., 2009)	N/A
D. melanogaster: ubi-E-cadherin-GFP	(Oda and Tsukita, 2001)	N/A
D. melanogaster: ubi-Bazooka-mCherry	(Bosveld et al., 2012)	N/A
D. melanogaster: sqh-Rok^{K116A}-Venus	(Simoes Sde et al., 2014)	N/A
D. melanogaster; tubP-GAL80[ts]	Bloomington Drosophila Stock Center	BDSC: 7018
D. melanogaster: pannier-GAL4	Bloomington Drosophila Stock Center	BDSC: 3039
D. melanogaster: UAS-Rok^{CAT}	(Verdier et al., 2006)	N/A
D. melanogaster: UAS-armadillo RNAi	Vienna Drosophila Resource Center	VDRC: KK107344
D. melanogaster: UAS-Shotgun RNAi	National Institute of Genetics Fly Stock Center	NIG: 3722R-1
D. melanogaster: UAS-Rok RNAi	Vienna Drosophila Resource Center	VDRC: KK104675
D. melanogaster: UAS-Slik RNAi	Vienna Drosophila Resource Center	VDRC: GD43783
D. melanogaster: UAS-sds22 RNAi	Vienna Drosophila Resource Center	VDRC: GD42051
D. melanogaster: UAS-MYPT75-D RNAi	National Institute of Genetics Fly Stock Center	NIG: 68976R-1
Software and Algorithms		
Prism 7 for Mac OS X	GraphPad Software, Inc.	N/A
MATLAB R2016a	The Mathworks, Inc.	N/A
Packing Analyzer V2.0	(Aigouy et al., 2010)	N/A
FIJI	(Schindelin et al., 2012)	N/A
Optical Flow Analysis	(Sun et al., 2010)	N/A

CONTACT FOR REAGENT AND RESOURCE SHARING

Further information and requests for reagents should be directed to and will be fulfilled by the Lead Contact, Buzz Baum (b.baum@ucl.ac.uk).

EXPERIMENTAL MODEL AND SUBJECT DETAILS

Drosophila Genetics

The following stocks were used: w^{1118} (Bloomington: 3605), *sqh::mCherry* (Martin et al., 2009), *sqh^{Ax3}; sqh*-GFP42 (Royou et al., 2004), DE-*cadherin-GFP* (Huang et al., 2009), *ubi-E-cadherin*-GFP (Oda and Tsukita, 2001), *ubi-Bazooka-mCherry* (Bosveld et al., 2012), *sqh-Rok^{K116A}-Venus* (Simoes Sde et al., 2014), *tubP-GAL80*[ts] (BL7018) . dsRNA interference (RNAi) and overexpression constructs were expressed using the UAS/GAL4 system (Brand and Perrimon, 1993; Busson and Pret, 2007). Notum-specific promoter: *pannier*-GAL4 (Bloomington: 3039). Rok overexpression: *UAS-Rok^{CAT}* (Verdier et al., 2006). The following RNAi lines were used to

silence gene expression: *armadillo* (VDRC: KK107344), *Shotgun* (NIG: 3722R-1), *Rok* (VDRC: KK104675), *Slik* (VDRC:GD43783), *sds22* (VDRC: GD42051), *MYPT75-D* (NIG: 68976R-1).

Adult fly crosses were kept at room temperature (21°C). Fly food recipe: 39 l dH2O, 675 g yeast, 390 g soy flour, 2.85 kg yellow cornmeal, 224g agar, 3 l light corn syrup, 188 ml propionic acid. After 2-3 days of egg laying, stocks were flipped and the tube, containing the eggs, was transferred to 18, 25, or 29°C for larval development. The incubation temperature used is stated with the experimental genotypes. AP age develops in real-time at 25°C, and at approximately half pace at 18°C. For the majority of imaging, pupae were staged at 12 h AP (at 25°C) for dissection and imaging, so at 0 h they were transferred to 18°C overnight for imaging the next day. For later developmental stages pupae were moved between 18 and 25°C accordingly.

Experimental Genotypes

FIGURE	EXPERIMENTAL GENOTYPE	INCUBATION (°C)	MICROSCOPE
1 (All) & S1	*; DE-cadherin-GFP ; pnr-GAL4 / + ;;*	25	Leica SP2
2 (All) & S2	*; DE-cadherin-GFP ; pnr-GAL4 / + ;;*	25	Leica SP2
3A	*w^{1118}; ; ubi-DE-cadherin-GFP / + ; pnr-GAL4 / + ;;*	25	Leica SP2
3A	*w^{1118}; ; ubi-DE-cadherin-GFP/ UAS-armadillo RNAi ; pnr-GAL4 / + ;;*	18	Leica SP2
3B	*w^{1118} ; DE-cadherin-GFP / + ; pnr-GAL4 / + ;;*	29	Leica SP2
3B	*; DE-cadherin-GFP / + ; pnr-GAL4 / UAS-shotgun RNAi ;;*	18	Leica SP2
3B	*; DE-cadherin-GFP / + ; pnr-GAL4 / UAS-armadillo RNAi ;;*	18	Leica SP2
4 (All) & S3 (All)	*sqh^{Ax3} / + or Y ; Sqh-GFP / If or Cyo ; ubi-Baz-mCh / + ;;*	25	Nikon Eclipse Ti-E
5B	*sqh^{Ax3} / + or Y ; Sqh-GFP / If or Cyo ; ubi-Baz-mCh / + ;;*	25	Nikon Eclipse Ti-E
6A	*sqh^{Ax3} / + or Y ; Sqh-GFP / If or Cyo ; ubi-Baz-mCh / + ;;*	25	Carl Zeiss Axiovert 200 M
6B	*w^{1118} ; DE-cadherin-GFP / + ; pnr-GAL4 / + ;;*	25	Carl Zeiss LSM510
6C-G & S5 (All)	*; DE-cadherin-GFP ; pnr-GAL4 / + ;;*	25	Leica SP2
7A	*; sqh-Rok^{K116A}-Venus / If or Cyo ; ubi-Baz-mCherry / + ;;*	25	Leica SP2
7B, D-E	*w^{1118} ; DE-cadherin-GFP / + ; pnr-GAL4 / + ;;*	29	Leica SP2
7B, D-E	*; DE-cadherin-GFP / UAS-Rok ^{RNAi} ; pnr-GAL4 / + ;;*	29	Leica SP2
7B, D-E	*; DE-cadherin-GFP / + ; pnr-GAL4 / UAS-Rok ^{CAT} ;;*	29	Leica SP2
7C	*w^{1118} ; DE-cadherin-GFP / + ; pnr-GAL4 / + ;;*	29	Carl Zeiss LSM510
7C	*; DE-cadherin-GFP / UAS-Rok ^{RNAi} ; pnr-GAL4 / + ;;*	29	Carl Zeiss LSM510
7C	*; DE-cadherin-GFP / + ; pnr-GAL4 / UAS-Rok^{CAT} ;;*	29	Carl Zeiss LSM510
S6A & F-G	*w^{1118} ; DE-cadherin-GFP / + ; pnr-GAL4 / + ;;*	29	Leica SP2
S6A & G	*; DE-cadherin-GFP / + ; pnr-GAL4 / UAS-sqh^{AA} ;;*	29	Leica SP2
S6A & G	*; DE-cadherin-GFP / + ; pnr-GAL4 / UAS-sqh^{EE} ;;*	29	Leica SP2
S6A & F	*; DE-cadherin-GFP / UAS-Slik ^{RNAi} ; pnr-GAL4 / + ;;*	29	Leica SP2
S6A & F-G	*; DE-cadherin-GFP / UAS-sds22 ^{RNAi} ; pnr-GAL4 / + ;;*	29	Leica SP2
S6A	*; DE-cadherin-GFP / UAS-moe^{TD559} ; pnr-GAL4 / $tubGal80^{ts}$;;*	18 transferred to 29°C 6 h prior to imaging	Leica SP2
S6B	*w^{1118} ; DE-cadherin-GFP / + ; pnr-GAL4 / + ;;*	29	Carl Zeiss LSM510
S6B	*; DE-cadherin-GFP / UAS-Slik ^{RNAi} ; pnr-GAL4 / + ;;*	29	Carl Zeiss LSM510
S6B	*; DE-cadherin-GFP / UAS-sds22 ^{RNAi} ; pnr-GAL4 / + ;;*	29	Carl Zeiss LSM510
S6C	*w1118 ; ubi-E-cadherin-GFP / + ; pnr-GAL4 / + ;;*	29	Leica SP2
S6C	*; ubi-E-cadherin-GFP / UAS-Slik ^{RNAi} ; pnr-GAL4 / + ;;*	29	Leica SP2
S6C	*; ubi-E-cadherin-GFP / UAS-sds22 ^{RNAi} ; pnr-GAL4 / + ;;*	29	Leica SP2
S6D & E	*w^{1118} ; DE-cadherin-GFP / + ; pnr-GAL4 / + ;;*	29	Leica SP2
S6D & E	*; DE-cadherin-GFP / UAS-Slik ^{RNAi} ; pnr-GAL4 / + ;;*	29	Leica SP2
S6D & E	*; DE-cadherin-GFP / UAS-sds22 ^{RNAi} ; pnr-GAL4 / + ;;*	29	Leica SP2
S7A-D	*w1118 ; ubi-E-cadherin-GFP / + ; pnr-GAL4 / + ;;*	29	Leica SP2
S7A-D	*; ubi-E-cadherin-GFP / UAS-Rok ^{RNAi} ; pnr-GAL4 / + ;;*	29	Leica SP2
S7A-B	*; ubi-E-cadherin-GFP / + ; pnr-GAL4 / MYPT75-D ^{RNAi} ;;*	29	Leica SP2
S7A-B	*; ubi-E-cadherin-GFP / UAS-sds22 ^{RNAi} ; pnr-GAL4 / + ;;*	29	Leica SP2

METHOD DETAILS

Notum Dissection for Live Imaging

For live imaging, flies were raised to 11.5 to 12 h AP (at 18, 25, or 29°C) and fixed to a microscope slide with double-sided Sellotape, as (Georgiou et al., 2008). The pupal case was removed to the abdomen to expose the notum. This is achieved by removing the operculum with forceps, cutting down the mediolateral side of the fly with dissection scissors and pealing the case off (Zitserman and Roegiers, 2011). A stack of 18 x 18mm slides was glued to the slide, with clear nail varnish, anteriorly (5 slides) and posteriorly (4 slides). A coverslip was spread with halocarbon oil 700 (Sigma – H8898) and rested onto the stacks so that it is in contact, but not crushing, the notum. The coverslip was glued in place with clear nail varnish at the posterior end. Flies were allowed to settle for 10 mins prior to imaging.

Microscopes Used for Live Imaging

The microscope used for each experiment is stated with the experimental genotypes:

Leica SPE2 scanning confocal with a 40x 1.3 NA oil objective.

For 30 s junction dynamics imaging. z-slices were 1μm, spanning 5μm and for long-term time-lapse at 5 min intervals (1μm x 20 z-slices).

Leica SP5 inverted confocal with a 40x 1.3 NA oil objective and 63x 1.3 NA oil objective.

For imaging of fixed samples.

Nikon Eclipse TI-E inverted system with a Yokogawa CSU-X confocal spinning disk unit fitted with an Andor EMCCD camera using a 100x 1.4 NA objective.

For two-color time-lapse (30 s interval) imaging.
Z-slices = 0.5 μm covering 4 μm. Maximum intensity projections were made of three slices in the focal plane for each time-point.
488 laser for GFP imaging at 5% power for 50 ms exposure. 561 laser for mCherry imaging at 20% power for 150 ms exposure.

Carl Zeiss LSM 200 M with a Yokogawa CSU-X confocal spinning disk unit and Andor Zyla cSMOS 5.5 camera with a 63x 1.3 NA water objective.

For two-color single time-point imaging at different stages of pupal development.
Z slices = 0.5μm covering 4μm. Maximum intensity projections were made of three slices in the focal plane.

Carl Zeiss LSM510 Meta upright confocal microscope with a Plan-Neofluor 40x/1.3 Oil DIC objective.

For laser ablation of adherens junctions.

Laser Ablation

Laser ablations were undertaken as (Marinari et al., 2012). Flies were prepared on a microscope slide for live imaging as described above. DE-cadherin-GFP was visualised using 488 nm light from an Ar-Kr laser with a Plan-Neofluor 40x/1.3 Oil DIC objective, coupled to a Zeiss LSM510 Meta upright confocal microscope. Image acquisition prior to, and after, ablation was at 1 s intervals. Junctions were ablated with 720 nm multiphoton excitation from a Chameleon-XR Ti-Sapphire laser (AIM, Zeiss). Junctions were ablated by scanning over a 3x3 pixel region of interest (0.009μm^2) at 25% laser power, for 1 iteration, with a dwell time of 2.56 μs / pixel.

Notum Dissection for Immunohistochemistry

For p-Myosin II staining 12-13 h AP nota were dissected in PBS before fixation in 4% paraformaldehyde in PBS for 20 min at RT. Samples were washed and permeabilized for 3x 10 min in PBT (PBS with 0.1% Triton X-100) at room temperature (RT). Nota were incubated with 1:1 PBT: blocking buffer (5% BSA, 3% FBS in PBS). Incubation with primary antibodies was undertaken at 4°C overnight (O/N) prior to 4x PBT 10 min washes. Incubation with secondary antibodies was carried out for 1-2 h at RT in PBT, with gentle shaking. Nota were 3x washed with PBS for 10 mins and kept in 50% glycerol in PBS at 4°C O/N before mounting. TritC-Phalloidin (1:500) and DAPI (1:2000) staining was undertaken during the second wash. Nota were mounted with 50% glycerol in PBS and imaged within days. The following primary and secondary antibodies were used: Phospho-Myosin Light Chain Ser19 (rabbit, 1:30 dilution, Cell Signalling 3671), anti-GFP (chicken, 1:500), goat anti-chicken Alexa 488 (1:250), goat anti-rabbit Alexa 647 (1:250).

Theoretical and Computational Models

Theoretical and computational models used in this study are described in Methods S1.

QUANTIFICATION AND STATISTICAL ANALYSIS

Image Processing and Statistics

All images presented were processed with FIJI software (http://fiji.sc/Fiji) (Schindelin et al., 2012) and Adobe Illustrator CS5.1 (Adobe Systems, Inc.). Graphs were produced using GraphPad Prism 6 (GraphPad Software, Inc). Statistical analyses were performed in Prism. D'Agostino and Pearson omnibus normality tests were used to determine if data were Gaussian. Nonparametric datasets were compared using a Kolmogorov-Smirnov test, or a Mann-Whitney test, where appropriate and stated in legend. Data from a Gaussian distribution were compared using unpaired Student's t-tests.

Laser Ablation Measurements

Kymographs were used in order to calculate vertex displacement velocities. In FIJI, straight lines (1 pixel thick) were drawn across the pre-ablated junction and the 'Dynamic Reslice' function was used to produce a kymograph (X = length, Y = time) of each ablation event. The 'segmented line' tool was used to plot the progression of each of the two junction vertices over time. In Matlab, the distance between the two X,Y vertex coordinates at each time point was calculated (function supplied below). Total displacement between vertices was then plotted in Prism at 10 sec after ablation. The first time frame after ablation was not used for this study, as the initial vertex displacement measure, because unlike in other tissues, such as the wing disc (Mao et al., 2013), measurable and significant displacement was not observed in the notum in the first frame after ablation.

In Excel, the laser ablation data should be saved as: Column A: Vertex 1 coordinate; Column B: Time Frame of V1; Column C: Vertex 2 coordinate; Column D: Time Frame of V2; Column E: Ablation Frame; Column F: Distance Scale; Column G: Time interval.

MATLAB function for quantifying distance between XY coordinates:

```
function magic1
clear all
close all
clc
[filename, url] = uigetfile('*.xlsx' , 'select data file' );
data = importdata([url, filename]);
vertex1 = data.data(:,1);
vertex2 = data.data(:,3);
time1 = data.data(:,2);
time2 = data.data(:,4);
vertex1(isnan(vertex1)) = [];
vertex2(isnan(vertex2)) = [];
time1(isnan(time1)) = [];
time2(isnan(time2)) = [];
startTime = max(min(time1),min(time2));
endTime = min(max(time1),max(time2));
timeInterval = 1; %seconds
time = (startTime:timeInterval:endTime)';
V1 = interp1(time1,vertex1,time);
V2 = interp1(time2,vertex2,time);
length = abs(V2-V1);
ablationTime = 0; %CHANGE
output = [time-ablationTime,V1,V2,length];
xlswrite('result.xls' ,output);
end
```

Aspect Ratio Quantification

For Figure 1F, the aspect ratio measure was made using FIJI, from an ellipse fit to a selection. Fit ellipse is found within FIJI at Analyze – Measure – Fit Ellipse. The macro used to fit an ellipse can be found at: http://imagej.nih.gov/ij/macros/DrawEllipse.txt. The area, centroid and orientation of the original selection are retained. The aspect ratio is calculated by dividing the major axis of the ellipse with the minor axis.

For Figures 1H–1J, the aspect-ratio for the four-cell clusters involved in a neighbor exchange was measured. The center of area (CoA) for each cell was calculated, and the internal aspect ratio defined as the distance between the CoA of cells losing an edge divided by that the cells gaining an edge. For the external aspect ratio, the axis between the CoAs was extended to the point it intersected with the perimeter. The external ratio was then calculated as the distance between perimeters of cell losing an edge, divided by the distance between perimeters of cells gaining an edge. This definition of the aspect ratio, would mean that an elongation of the four-cell cluster along the same axis as the expansion of the T1 junction, would give an increase in aspect ratio.

Image Segmentation

Images were segmented using Packing Analyzer V2.0. Tutorials are available at: https://idisk-srv1.mpi-cbg.de/∼eaton/ (Aigouy et al., 2010).

Protocol:

- Save movie in FIJI as an 8-bit Image Sequence TIFF.
- Open Packing Analyzer, drag and drop the Image Sequence into the List.
- Under Init, change the parameters to 3.8 (top) and 1.9 (bottom), or change these parameters as you see fit.
- Click Detect Bonds. A notum image with the above parameters is likely to be over segmented. Save.
- Under Correction, correct the mistakes. Right click to delete junctions. Left click and hold to draw new junctions. Change the pencil size, on the left above the tabs. Use a large pencil size to delete lots of junctions at once. Click second green tick to apply changes. Save.
- To apply changes to the next image in the sequence click Seed Next.
- Correct errors. Save. Seed next. Continue to the end of the Image Sequence.
- Once finished, Post Process, change 4 way vertices cutoff to 3. Click Finish All. (large datasets will take 10-15 mins).
- Recenter tab, click autocenter based on 2D correlation (10-15 mins).
- Tracking, click Track cells (10 mins).
- Tracked bonds, click track bonds (10 mins).
- Plots, can plot all bonds or plot all cells. To plot individual cells/junctions draw lines through cells/junction of interest in Current Image then click plot selected cells / plot selected bonds. Always exclude border bonds or cells.
- Virtual cloning, draw a line through cells of interest in Current Image, click track clones. Can go back to Plots and plot clone info.
- Under the Viewer tab you can see a range of segmentations for each image.
- In the folder where the original Image Sequence is saved, each image will have a folder containing all the files produced.
- Number the bd_fate files and create an Image stack in FIJI.
- Plots will be saved as .csv files in first image folder ….000.
- Open in Excel, highlight first column. Go to Data, and click Text to columns. Highlight the whole dataset and go to Data, Sort. Sort by ID then Frame Number. Sizes (junction lengths and areas) are in pixels.

Analyzing Junction Fluctuation Behavior

In order to extract data for the behavior of junctions and cells in a format amenable to the type of analysis we wanted to do, we developed a custom software package. The code is written in Matlab using class-based object-oriented programming. It detects junctions and cells from segmented images, corrects for drift, tracks junctions and cells between frames, and calculates connectivity within the tissue. This makes it possible to extract time series data for various properties, analyze spatial correlations, and detect when cells change neighbors.

The flow of data within the code is as follows:

- Input segmented time-lapse images.
- In case of microscope drift, generate set of stabilized images by subtracting net translation.
- Identify individual junctions in each image and store these as objects.
- Track junctions between frames and assign a unique ID to each junction.
- For each junction, find the IDs of neighboring junctions.
- Detect individual cells in each image and store these as objects.
- Track cells between frames and assign a unique ID to each cell.

The input for the code are segmented 8-bit image sequences of the *Drosophila* notum produced with Packing Analyzer V2.0 (Aigouy et al., 2010), tutorials are available at: https://idisk srv1.mpi-cbg.de/∼eaton/. Segmentation results in skeletonized images where the width of a cell-cell interface (the junction) is 1 pixel.

In some cases, the tissue drifts relative to the microscope during imaging. The algorithm for tracking junctions and cells cannot deal with large-scale deformation or significant displacement of the tissue between frames. To deal with this, we used Optical Flow Analysis (modified from the OFA algorithm available at http://cs.brown.edu/people/black/code.html and described in Sun et al., 2010) to calculate the flow field for each consecutive pair of frames. Taking the average of the flow field gives the direction and magnitude of the net translation of the tissue. Mapping the images into a larger space, by subtracting the cumulative net translation for each time point, yields a set of stabilized images that can be input into the code. The first part of the code identifies individual junctions in each image. Specifically, the code initiates a junction and 'walks' along the bright pixels in the image, storing the coordinates along the way, then terminating the junction when a vertex is reached. This is then repeated until all pixels in the image have been visited. Within the code, each junction is an object with associated properties. At this stage, only the fields for the vertex coordinates and junction coordinates are filled.

The code then calculates various properties of junctions, including: vertex 1 and 2 positions, junction coordinates, length, midpoint, angle and neighbor IDs. Since junctions can be curved, the vertex-vertex distance is not an accurate measure of junction length. In

addition, discretisation has the consequence that calculating the length by summing the distance between pixels along the segmented junction would slightly overestimate the length. Instead, junction length is calculated as the diagonal distance connecting consecutive blocks of pixels.

The next step involves tracking junctions between frames and assigning a unique ID to each junction, to make it possible to extract time series for various properties and detect changes in connectivity. The tracking of junctions is done by finding the midpoints of junctions and using these coordinates as the input for a particle tracking algorithm (based on code available from the Mathworks repository, written by John C. Crocker (Crocker et al., 1996)). The tracking algorithm takes the coordinates of the midpoints at time t and considers all possible matches with the midpoints at time t + 1 to choose the pairings that minimize the total squared displacement. This is then used to assign each junction a unique ID that identifies it across frames. The results were validated by visual inspection of the assigned IDs – specifically by creating a movie of junctions colored according to their ID, making errors in continuity easy to spot. Having assigned a unique ID to each junction, the connectivity of the tissue is found and the IDs of neighboring junctions are stored. In addition to junctions, the code also detects and tracks cells in the time-lapse images and stores each as an object. The properties for this class include: Cell ID, junction IDs, midpoint, area, perimeter, vertices and angles. Cells are detected using the junction objects. Specifically, the code starts at one junction and moves to neighboring junctions in a clockwise fashion until getting back to the first one, thereby identifying the junctions that make up a cell. This is repeated in a counterclockwise fashion for the same junction. To avoid storing the same cell multiple times, the code loops over junctions starting from j = 1 and requires that only neighboring junctions with larger values of j can be traversed - if that is not available, the code breaks and starts from a different junction instead. Next, the area, perimeter length and midpoint of each cell is calculated. The midpoints are used to track cells between frames and assign unique IDs in the same way as described for junctions.

Detecting Neighbor Exchange Events

Neighbor exchange events are difficult to quantify manually. Therefore, we wrote an algorithm to detect transitions and make it possible to extract quantitative data related to the junctions and cells involved. The code detects all junctions that contract to a four-way vertex and expand back out, and determines whether they change neighbors in the process. For the DE-cadherin-GFP imaging at 30 s intervals on the Leica SP2 scanning confocal, the diameter of four-way vertices is $\sim$6 pixels, corresponding to 538 nm. Segmentation of very short junctions, and especially four-way vertices, is difficult and error-prone. In particular, segmented short junctions tend to 'flip', changing orientation and neighbors. If not corrected, such false neighbor exchange events would bias subsequent analysis. To ensure the quality of the data that form the basis of this study, we manually checked every computationally detected event by looking at the corresponding junction in the fluorescent time-lapse images. We used the criteria that the extension of a junction, coming from a four-way vertex configuration, should be stable and the change in cell neighbors should be clearly visible in the fluorescent images. Events that did not fit these criteria were excluded from subsequent analysis. In addition, we checked that the time point for the event, as identified by the algorithm, was consistent with when a four-way vertex was reached in the fluorescent images.

Fluorescence Intensity Measurements

We concurrently imaged Myosin II and junction dynamics using transgenic fly stocks expressing both Sqh-GFP (Myosin) and Bazooka-mCherry (adherens junction marker). To correlate Myosin II intensities with junction dynamics (Figure 4), we developed code to extract the time series data for Myosin intensities on individual junctions. For each junction, we used the pixel coordinates from the segmented images to identify the junction in the fluorescent images. To include the fluorescence intensity across the width of the junction, we performed a morphological dilation to give each junction an average width of 7 pixels. This corresponds to a width of 488 nm, for the time-lapse imaging taken at 30s intervals with a resolution of 0.06974 mm/pixel. The vertices tend to be the brightest regions in the image and including them would give rise to artificial artifacts in the correlation functions - e.g. as junctions contract the vertices would make up a larger proportion of the junction resulting in an increase in the average intensity per pixel. The vertex itself also belongs to more than one junction, and therefore they were excluded from the analysis.

For each junction, we sum over the intensity of pixels within the region covered by the morphological dilation. There is a slight bleaching of the tissue over time, leading to a gradual decrease in intensity. We remove the trend associated with bleaching in the following way: for each time frame, we sum the total intensity for all pixels within the dilated junctions (I_{tot}) and calculate the total number of pixels (p_{tot}). For each junction, the total intensity I_j, is normalized by I_{tot}/p_{tot}, such that the average intensity per pixel is equal to 1 for every time frame $\sum_j I_j / \sum_j p_j = 1$. For each junction, we calculate the 'normalized average intensity' by taking the total intensity for a junction, normalizing it as described, then dividing by the number of pixels in the junction. Qualitatively, dividing by the number of pixels gives the same results as dividing by junction length.

Developmental Cell, Volume *43*

Supplemental Information

Myosin II Controls Junction Fluctuations

to Guide Epithelial Tissue Ordering

Scott Curran, Charlotte Strandkvist, Jasper Bathmann, Marc de Gennes, Alexandre Kabla, Guillaume Salbreux, and Buzz Baum

Supplemental Inventory

Supplemental Figures

Figure S1: Related to Figure 1.

Figure S2: Related to Figure 2.

Figure S3: Related to Figure 4.

Figure S4: Related to Figure 5.

Figure S5: Related to Figure 6.

Figure S6: Related to Figure 7.

Figure S7: Related to Figure 7.

Supplemental Tables

Table S1: Related to Figure 2A-D and Figure S2.

Supplemental Movies

Movie S1: Related to Figure 1.

Movie S2: Related to Figure 1.

Movie S3: Related to Figure 5.

Movie S4: Related to Figure 6.

Supplemental Theory

Methods S1: Related to STAR Methods

Supplementary Figure 1

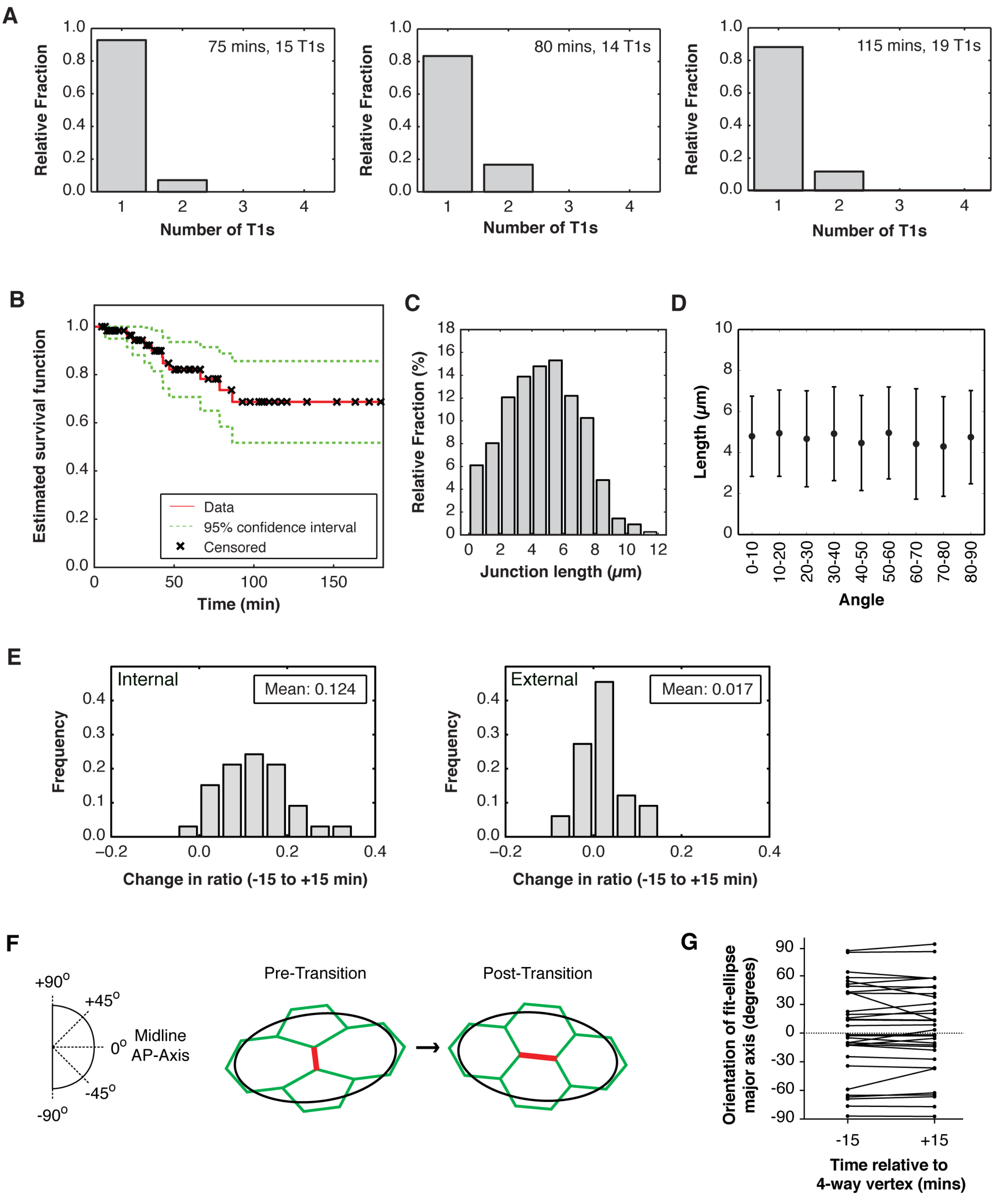

Figure S1. Related to Figure 1. Neighbour exchange events are reversible

(A), Bar charts showing the proportion of junctions that undergo unidirectional (one), bidirectional (two), and multidirectional (three or more) neighbour exchange events. n = 3 nota (not including nota from Figure 1C) over 75, 80, and 115 mins, respectively, labelled with DE-cadherin-GFP and imaged at 30 s intervals. **(B)**, Kaplan-Meier survival curve showing the probability that a neighbour exchange configuration persists for a given length of time. Events are 'censored' if the reverse transition has not occurred by the end of the movie.

The 95% confidence intervals are estimated using Greenwood's Formula. The probability of a configuration persisting for at least 150 min, along with the 95% confidence interval = 0.687 [0.5175, 0.8567]. **(C)**, Histogram of junction lengths in a single frame at 12h AP. n = 771 junctions / 3 nota. Average junction length = 4.69 ± 2.315µm. **(D)**, Junction lengths in a single frame at 12 h AP versus angle, with respect to the AP midline (0°). n = 771 junctions / 3 nota. **(E)**, Related to Figure 1H. Change in aspect ratio from t = -15 mins to t = +15 mins. A paired t-test was used to compare the aspect ratios at t = -15 mins and t= + 15 mins. Internally, the aspect ratio changes during a T1 event ($p < 0.0001$). Externally there is no change in aspect ratio ($p = 0.0502$). n = 33 exchange events from 4 flies. **(F)**, Diagram of a neighbour exchange event. The red junction represents the transition junction that is lost and gained. An ellipse (black) is fit to the four cells involved in the transition, and the feret angle of the ellipse is measured with respect to the midline (0°). **(G)**, The feret angle of fit ellipses are plotted at t=-15, and t=+15 mins, with paired results connected by a line. n = 33 exchange events from 4 flies.

Supplementary Figure 2

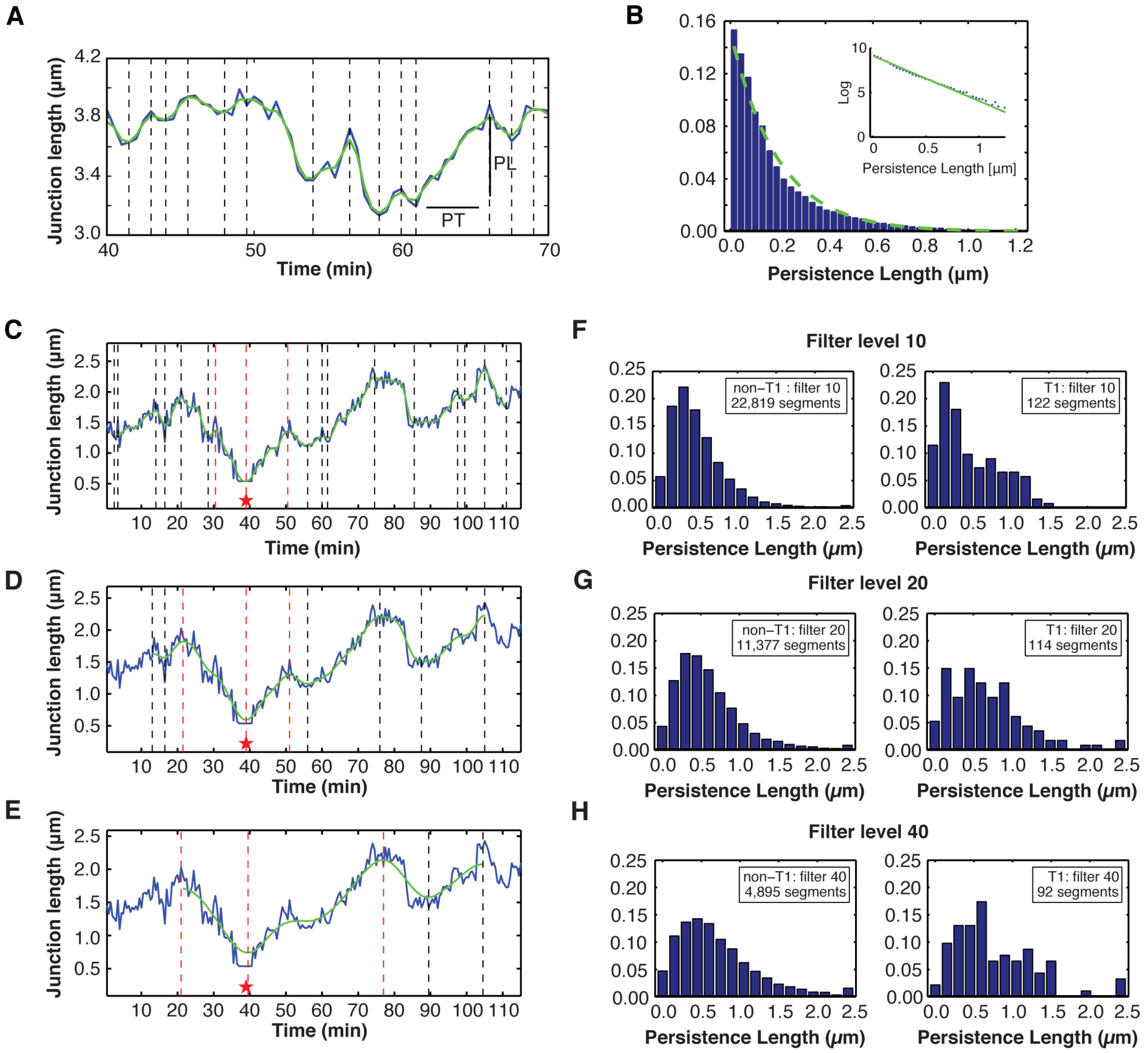

Figure S2. Related to Figure 2. Persistence length analyses results are consistent for a range of filter settings

(A), Time series line plot for a junction fluctuating in length. The data have been filtered using a moving average Hanning window, with a filter setting of 5, and split into segments where the junction is contracting or expanding. The persistence time (PT) and persistence length (PL) are defined as the duration of a segment and the change in junction length, respectively. **(B)**, Histogram of persistence lengths for a filter setting of 5. n = 60737 segments, 4 nota (green line). Best fit for an exponential function of the form $1/\tau\ exp(-t/\tau)$. The parameter is τ = 0.197 mm, with confidence bounds [0.1958 0.1989]. Inset, Number of segments in each bin of the histogram plotted on semi-log along with the exponential fit. **(C-E)**, Line plots of the length of a representative junction (blue) over time, for a junction that undergoes a neighbour exchange event at 40 min (marked by a red star). The time series has been split into segments where the junction length is monotonically increasing or decreasing. This was achieved by filtering the data using a moving average Hanning window

(green) - if the first derivative changes sign or is zero with opposite signs on either side of that point, a segment boundary is placed. Using a higher setting for the filter, results in fewer segments. The filter settings used are (**C**) 10, (**D**) 20 and (**E**) 40. The analysis is done using the raw data - the filter is only used to establish the position of the segment boundaries. The segments on either side of a T1 event (marked with red dotted lines) are defined as T1-segments. **(F-H)**, Persistence length distributions for (left) non-T1 and (right) T1 segments. The filter settings used are (**F**) 10, (**G**) 20, (**H**) 40, as shown in **C-E**. The number of segments included in each histogram are stated in the top right of each graph. Higher levels of filtering result in longer, and therefore fewer segments. The first and last segment of each time series is excluded from the analysis, regardless of whether a T1 event occurs (therefore the number of T1 segments included can vary with filter level). The distributions for T1 and non-T1 segments are compared used a two-sample Kolmogorov-Smirnov test. The p-values are listed in Table S1 and in every case the statistical test, at the 0.05 significance level, supports the null hypothesis that the samples are drawn from the same underlying distribution.

Supplementary Figure 3

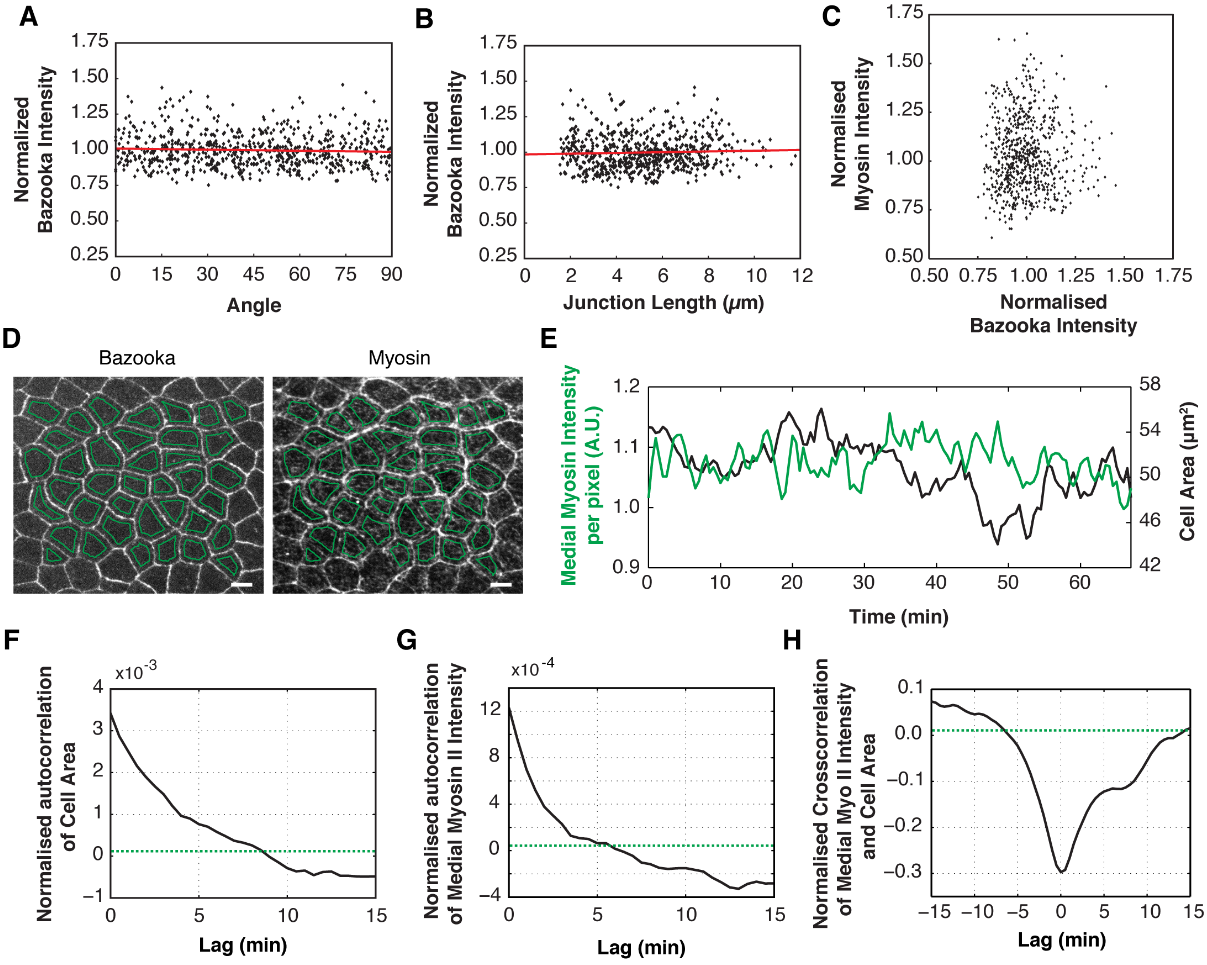

Figure S3. Related to Figure 4. Myosin and Bazooka intensities are uncorrelated and do not exhibit tissue-wide polarity. Cell areas and medial Myosin intensity do not display pulsatile behaviour and changes in medial Myosin intensity do not precede changes in cell area.

(A), Normalized junction intensities of Baz-mCh versus junction angle, as measured with respect to the AP midline (0°). Slope (with 95% confidence bounds) = -0.0003 (-0.00061 0.00011). Spearman's Rank = -0.048. n = 688 junctions / 3 nota. **(B)**, Normalized junction intensities of Baz-mCh versus junction length. Slope (with 95% confidence bounds) = 0.003 (-0.0019 0.0070). Spearman's Rank = 0.058. n = 688 junctions / 3 nota. **(C)**, Normalised (to mean intensity) paired junctional intensities of Baz and Myo-II show no correlation. Spearman's Rank = 0.024. n = 688 junctions / 3 nota. **(D)**, Apical surface projection of a live nota imaged with Bazooka-mCherry (left panel) and Spaghetti-Squash-GFP (right panel). Scale bar = 5μm. The medial region included in the analysis is outlined in green. **(E)**, A line plot showing a representative example of medial Myo-II-GFP (green) and cell area (blue) plotted as a function of time. Myo-II-GFP intensity is normalised to mean tissue intensity and the line plot shows the average Myo-II-GFP intensity per pixel. **(F)**, Mean autocorrelation for cell area variation of individual cells $\overline{\Delta A(t)\Delta A(t+\Delta t)}/(\bar{A})^2)$ as a function of lag time Δt. The autocorrelation is normalised by the time-averaged cell area A for each cell. n = 115 cells / 3 nota. **(G)**, Mean autocorrelation for medial Myo-II-GFP intensity in individual cells $\overline{\Delta I(t)\Delta I(t+\Delta t)}/(\bar{I})^2)$ as a function of lag time Δt. The autocorrelation is normalised by the time-averaged medial intensity for each cell. n = 115 cells / 3 nota. **(H)**,

Mean normalised crosscorrelation for medial Myosin intensity and cell area $\overline{\Delta I(t)\Delta A(t+\Delta t)}/\sigma^I\sigma^A$ as a function of lag time Δt, with σ^I and σ^A the intensity and cell area S.D. The minimum occurs at zero lag. The normalised crosscorrelation is calculated for each cell and then averaged over all cells in the analysis. n = 115 junctions / 3 nota, imaged at 30 s intervals for 60 mins.

Supplementary Figure 4

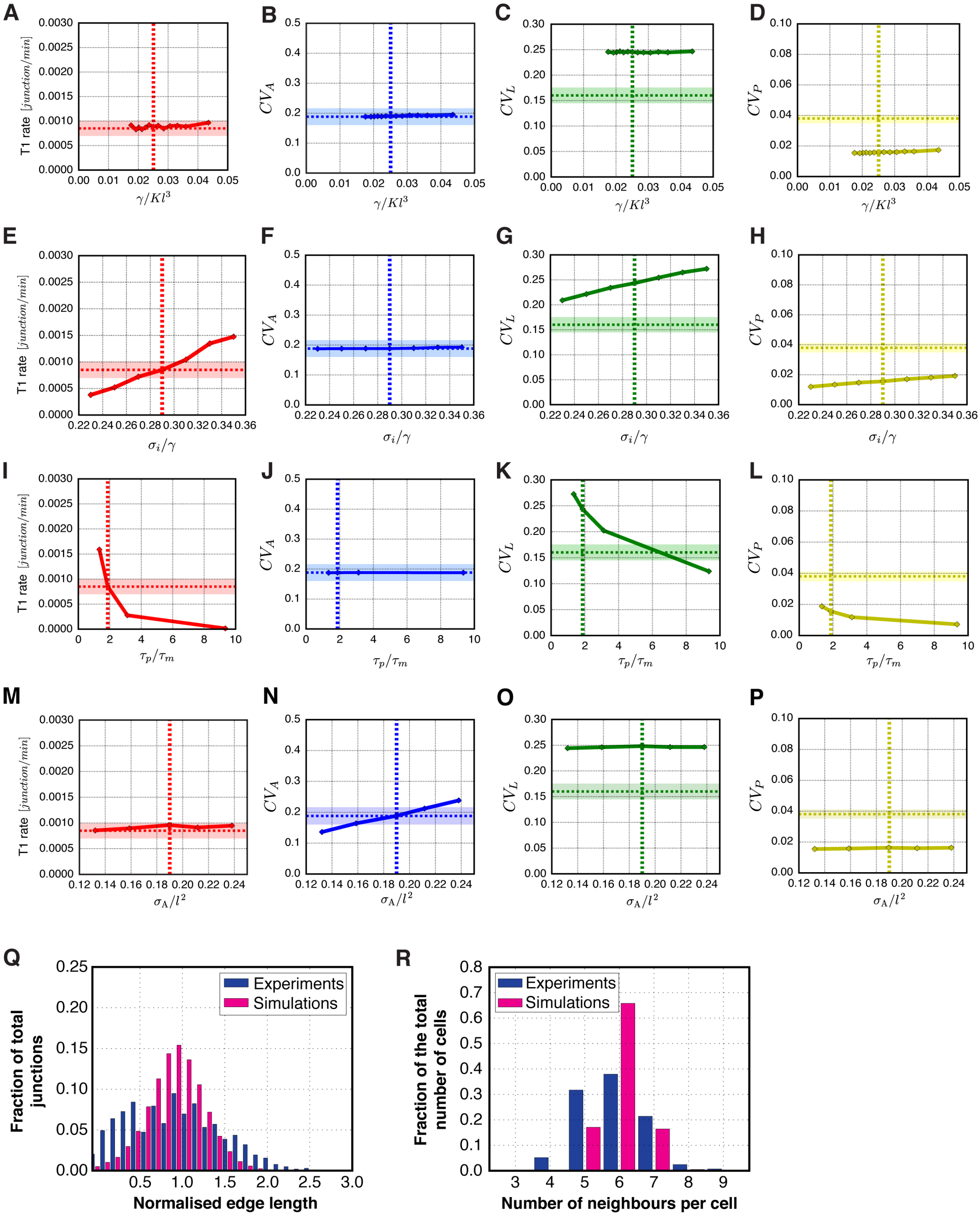

Figure S4. Related to Figure 5. 2D vertex model fitting parameters

(A-P) Plots showing the dependency of tissue properties on the fitting parameters. **(A, E, I, M)** T1 transition rate, **(B, F, J, N)** extrinsic area fluctuations (CV_A), **(C, G, K, O)** junction length intrinsic fluctuations (CV_L), and **(D, H, L, P)** relative perimeter fluctuations (CV_P), (dependency on the dimensionless model parameters: **(A-D)** dimensionless ratio of mean line tension over cell bulk elastic modulus (γ/Kl^3), **(E-H)** normalized intrinsic

noise intensity (σ_i/γ), **(I-L)** characteristic packing time (τ_p/τ_m) and **(M-P)** normalized standard deviation of preferred cell areas (σ_A/l^2). Dotted horizontal lines: experimental measurement, with shaded area indicating standard deviation. Dotted vertical lines: parameters used in wild-type simulations. **(Q)** Junction length distribution of simulations with wild type parameter settings to data obtained from experiments. **(R)** Polygon distribution of simulations with wild type parameter settings and data obtained from experiments.

Supplementary Figure 5

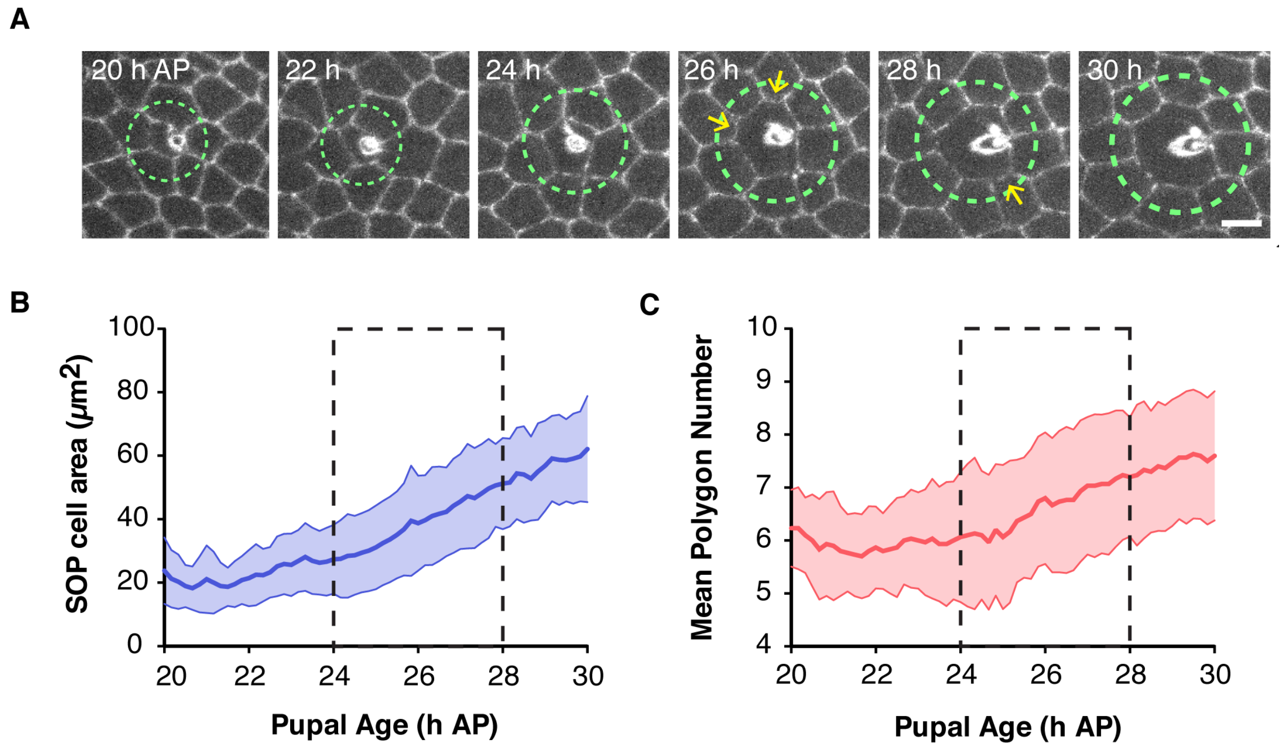

Figure S5. Related to Figure 6. Increasing neighbour exchange rates observed between 24 and 28 h AP are due to sensory organ precursor cell growth.

(A) Time-lapse montage of a representative SOP cell (circled by green dotted line), labeled with DE-cadherin-GFP, imaged from 20 to 30 h AP. Yellow arrows, label junctions that have been gained via neighbour exchange events. Scale bar, 5 μm. **(B-C)** Line plots showing the mean (with S.D) **(B)** SOP cell area and **(C)** the mean number of neighbours (polygon number) for SOP cells, from 20 – 30 h AP. Dotted box from 24-28 h AP indicates the time at which the T1 transition rate, as shown in Fig 6G, increases. n = 30 SOP cells / 3 nota.

Supplementary Figure 6

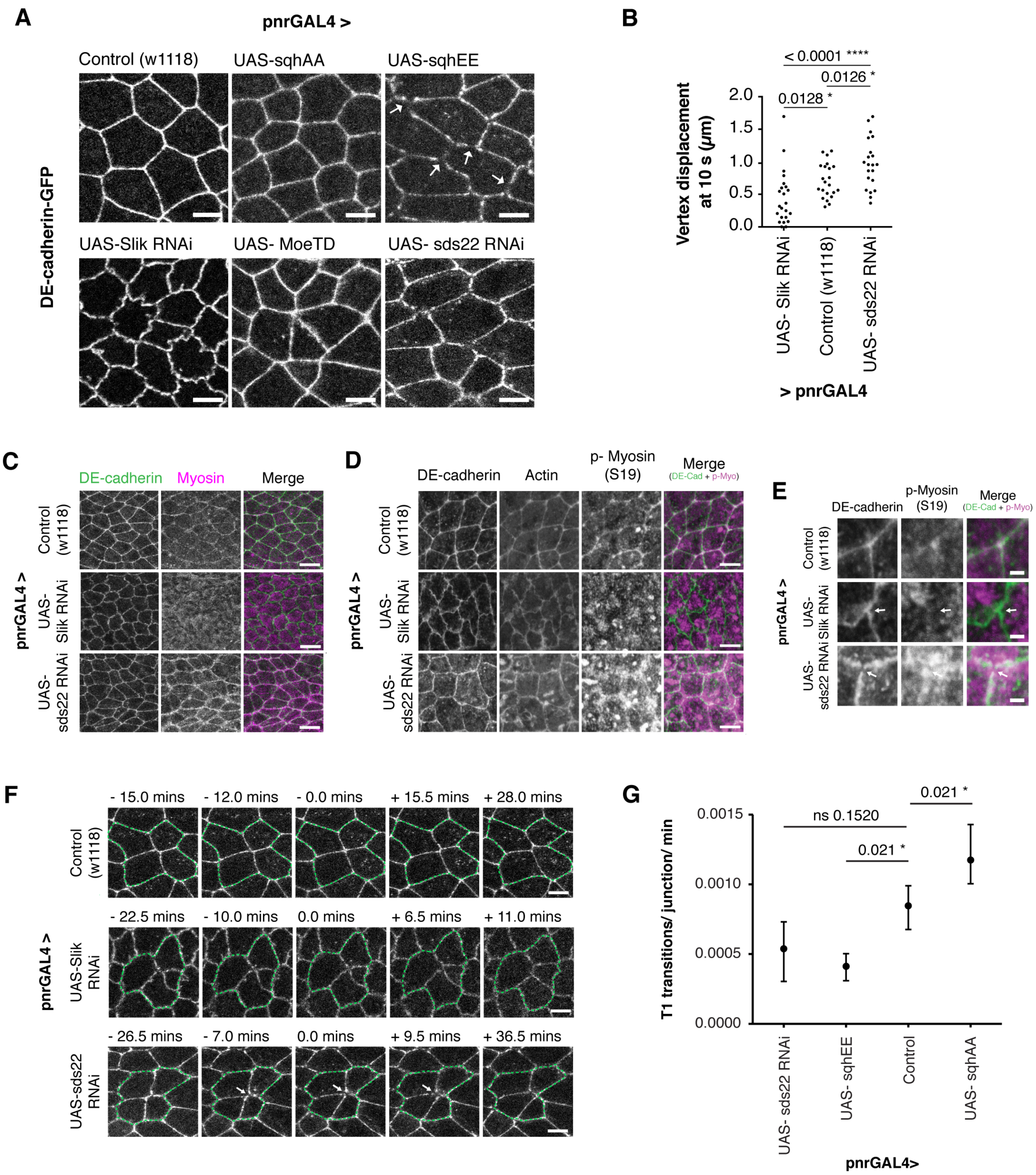

Figure S6. Related to Figure 7. Decreased Moesin activity causes a loss of junctional Myosin II and increases the medial pool. Phospho-null and phospho-active Myosin increase and decrease neighbour exchange rates, respectively.

(A) Apical surface projections of DE-cadherin-GFP labelled nota for control, UAS-sqhAA (Phospho-null Myosin II), UAS-sqhEE (Phospho-active Myosin II), UAS-Slik RNAi (decreased Moesin activity – note wiggly junction phenotype caused by pulling of medial Myosin on the junction), UAS-Moesin-TD (increased Moesin activity) and UAS-sds22 (increased Myosin and Moesin activity) driven by pnr-GAL4. Scale bar = 5μm. **(B)** Quantification of total vertex displacement at 10 s after laser dissection of single junctions in 12-13.5 h AP

pupae expressing Slik RNAi and sds22 RNAi. Dots indicate individual experiments, line represents median. 5-7 flies/condition. P-values calculated from unpaired t-tests. **(C)** Maximum surface projection images of nota live-imaged with ubi-DE-cadherin-GFP and Spaghetti-Squash-mCherry (MRLC) labelling total Myosin levels for control, Slik RNAi (note lack of Myosin on junctions) and sds22 RNAi (increased junctional Myosin). **(D)** Fixed-stain images for DE-cadherin-GFP (anti-GFP), F-actin (Phalloidin) and p-Myosin II (S19) for control, reduced Moesin activity (Slik RNAi) and increased Myosin and Moesin activity (sds22 RNAi). Scale bar = 5μm. **(E)** Increased zoom of vertices in **D**. Arrows for Slik RNAi highlight DE-cadherin junction, and absence of p-Myosin. Arrows for sds22 RNAI label junction break at 3-way vertex. Scale bar = 1μm. **(F)** Apical surface maximum projection montages of DE-cadherin-GFP labelled nota showing representative neighbour exchange events for control, sds22 RNAi and Slik RNAi. White arrows label junction breaks at the vertex during the sds22 RNAi transition. Slik RNAi nota can still undergo neighbour exchange in the absence of junctional Myosin – akin to Rok RNAi. **(G)** Quantification of normalised T1 transition rates for altered levels of Myosin activity. Dot indicates mean, tails show the data range. n = 3-4 flies / condition. P-values calculated from Kolmogorov-Smirnov tests.

Supplementary Figure 7

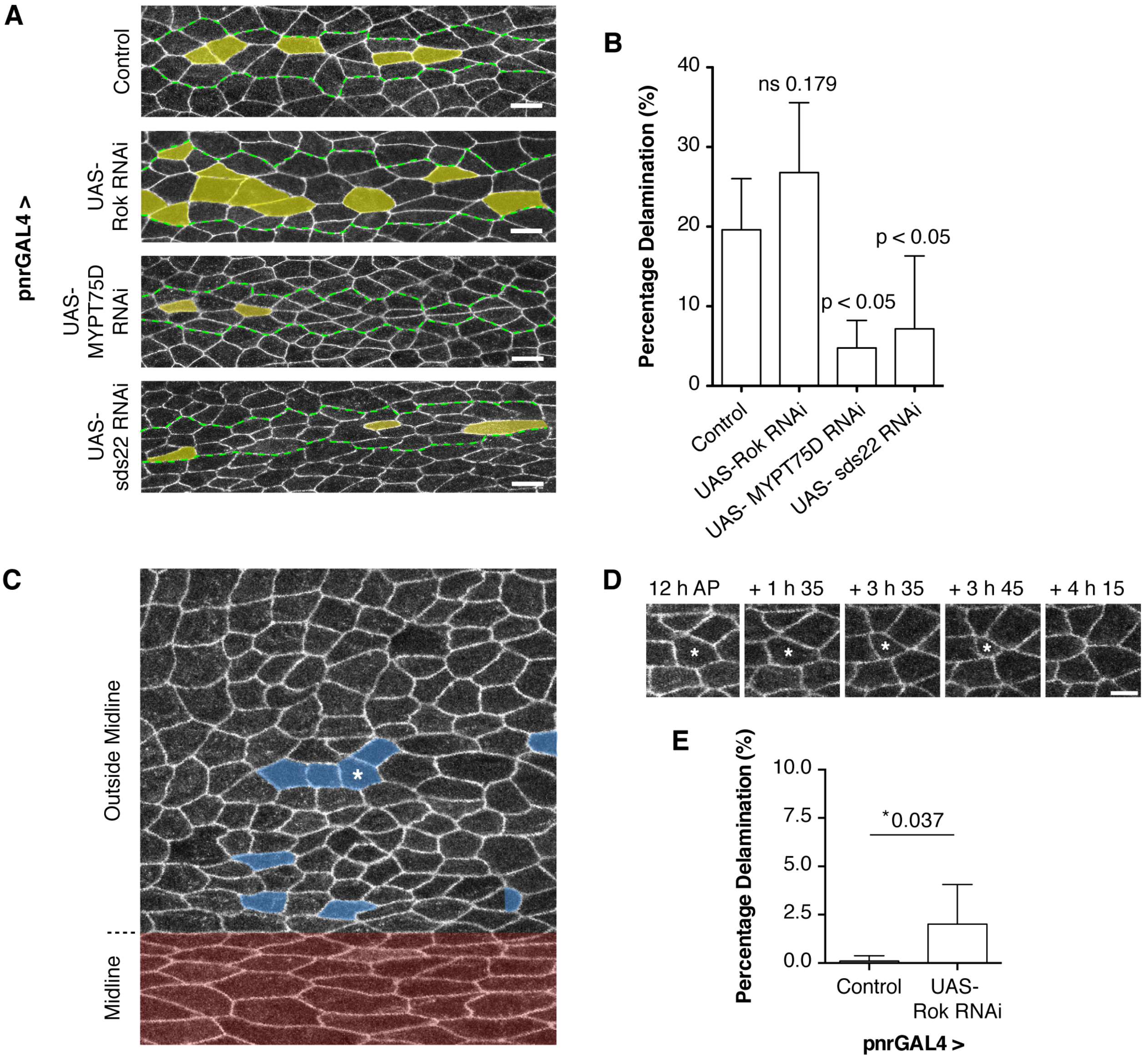

Figure S7. Related to Figure 7. A tissue-wide reduction in Myosin activity increases midline delamination levels and causes cells to delaminate outside of the midline.

(A) Apical surface maximum intensity projections of representative nota, labelled with ubi-E-cadherin-GFP, for altered Myosin II activity, showing the midline (dotted green line) with cells that delaminate (yellow) up to 18 h AP. Scale bar = 5μm. **(B)** Bar chart of percentage midline delamination for decreased (Rok RNAi) and increased (MYPT75-D RNAi and sds22 RNAi) Myosin II activity. Bar values indicate mean. P-values calculated from unpaired t-tests vs control. N = 4-6 nota per condition. **(C)** A representative notum with cells labelled outside of the midline that undergo basal delamination (blue). Midline region shaded red. Half blue cell indicates a daughter cell that delaminates after division. **(D)** Time-lapse montage of a cell (star labelled in **C**) that progressively loses junctions, and area, through time before extrusion. **(E)** Delamination levels were measured outside of the midline up to 18 h AP for control and Rok RNAi. Delamination levels were measured as a percentage of the total number of outer midline cells at 12 h AP. N = 6-7 flies with 110-237 (mean = 156) cells at 12 h AP. p-value calculated from a Mann-Whitney statistical test.

Supplementary Tables

Filter Level	P-value	Hypothesis
10	0.5142	H_0
15	0.2727	H_0
20	0.5294	H_0
25	0.2569	H_0
30	0.1081	H_0
35	0.1881	H_0
40	0.2572	H_0

Table S1. Related to Figure 2A-D and Figure S2.

The persistence length distributions for T1 and non-T1 segments are compared using a two-sample Kolmogorov-Smirnov test. The null hypothesis, H_0, is that the samples are drawn from the same underlying distribution. The analysis was carried out for seven different levels of filtering, between 10 and 40, and in every case the statistical test supports the null hypothesis at the 0.05 significance level. Note that, since we are testing a set of statistical inferences simultaneously, the appropriate significance level for the individual hypothesis tests is lower than the significance level for the set as a whole. Using the Bonferroni correction, we would get $\hat{}a = a/k$ = 0.05/7 = 0.007. P-values are all above 0.05.

Supplementary Movie Legends

Movie S1. Related to Figure 1.

This movie shows a region outside of the midline, of a wild-type developing pupal notum, visualised with DE-cadherin-GFP. The movie covers the period 12-13.5 h AP, prior to the onset of cell division and delamination, during which time cells undergo neighbour exchange (indicated in Figure 1), caused by fluctuations in junction length (indicated in Figure 2). Time interval between frames is 30 s and the video length is 1 h 30 mins. Scale bar, 10 μm.

Movie S2. Related to Figure 1.

This movie shows a four-cell cluster undergoing a uni-directional neighbour exchange event (indicated in Figure 1), visualised with DE-Cadherin-GFP. This is an event that occurs in Movie S1. Successive yellow arrows throughout the movie label the junction that is lost, the four-way vertex generated through junction loss, and the subsequent newly formed junction. Note neighbour exchange events also occur on the edge of the cluster throughout the movie. Time interval between frames is 30 s and the video length is 1 h 30 mins covering 12 - 13.5 h AP. Scale bar, 10 μm.

Movie S3. Related to Figure 5.

This movie shows three simulations of the 2D vertex model at low (0.8), control (1.0) and high (1.2) mean line tensions (γ/γ_0) (indicated in Figure 5). The edge colour, of each interface, corresponds to the level of line tension relative to it's mean (blue = low, red = high). Virtual time interval between frames is 15 s, with the video length 83 min 15 s.

Movie S4. Related to Figure 6.

This movie shows an example of a control laser ablation experiment (indicated in Figure 6B and 7C). Junctions outside of the midline are visualised with DE-cadherin-GFP between 12 and 13.5 h AP. The time interval between frames is 1 s with the laser ablation occurring at t = 0, indicated by a yellow star. The video length runs from 16 s pre-ablation to 120 s post-ablation. Scale bar, 5 μm.
